# Supplementary material for: Gene body DNA hydroxymethylation restricts the magnitude of transcriptional changes during aging
Source: Nat Commun. 2024 Jul 28;15:6357. doi: 10.1038/s41467-024-50725-y (PMC11284234; doi:10.1038/s41467-024-50725-y)
Supplement: Supplementary file 1 — Supplementary Information [file 41467_2024_50725_MOESM1_ESM.pdf]

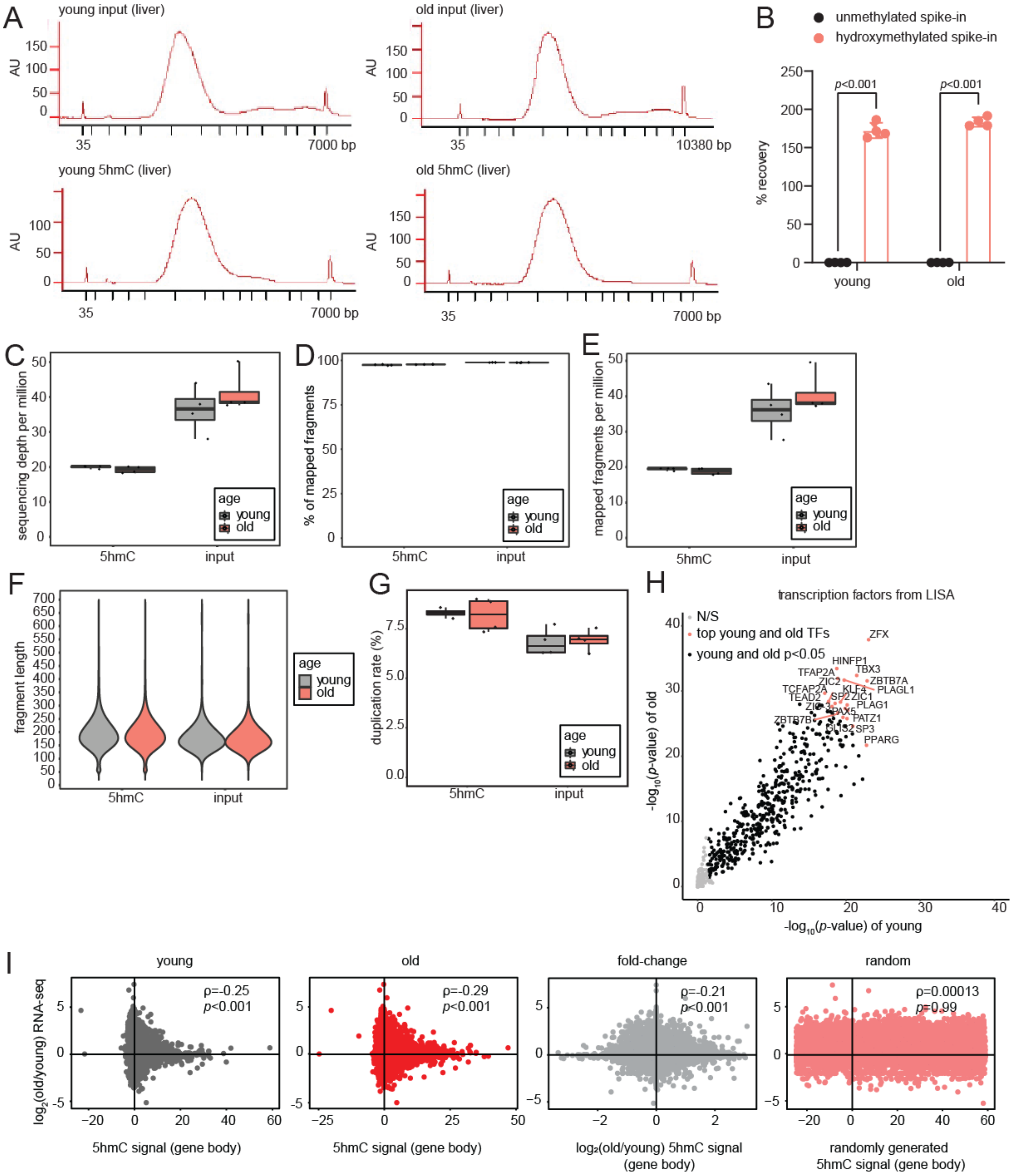
A
young input (liver)
old input (liver)
young 5hmC (liver)
old 5hmC (liver)
AU
35
7000 bp
10380 bp
B
unmethylated spike-in
hydroxymethylated spike-in
% recovery
p<0.001
young
old
C
sequencing depth per million
D
% of mapped fragments
E
mapped fragments per million
age
young
old
5hmC
input
F
fragment length
G
duplication rate (%)
H
transcription factors from LISA
N/S
top young and old TFs
young and old p<0.05
-log10(p-value) of old
-log10(p-value) of young
ZFX
HINFP1
TBX3
TFAP2A
ZIC2
ZBTB7A
TCFAP2A
KLF4
PLAGL1
TEAD2
SP2
ZIC1
PLAG1
PAX5
ZBTB7B
PATZ1
GLIS2
SP3
PPARG
I
young
old
fold-change
random
ρ=-0.25
p<0.001
ρ=-0.29
ρ=-0.21
ρ=0.00013
p=0.99
log2(old/young) RNA-seq
5hmC signal (gene body)
log2(old/young) 5hmC signal (gene body)
randomly generated 5hmC signal (gene body)

Supp. Figure 1: Quality control metrics of mouse liver hMeDIP-seq libraries

(**A**) (Left) Representative Bio-analyzer electropherogram of liver hMeDIP-seq library from young input and 5hmC samples (n=4). (Right), same as left but for old liver samples (n=4). AU represents arbitrary fluorescence units. (**B**) qPCR analysis for exogenous unmethylated and hydroxymethylated DNA control spike-ins included in hMeDIP-seq for young and old (n=4 each) samples. Data are presented as mean ± SD; statistical significance was assessed using two-way ANOVA with Šídák's multiple comparisons post-hoc test. Figure panels (C-G) present QC data from 5hmC and input libraries of young and old (n=4 each) mouse liver samples, including (**C**) sequencing depth, (**D**) percent of alignment rates of 5hmC and input libraries to the GRCm38/mm10 genome, (**E**) the number of fragments mapped to the GRCm38/mm10 genome, (**F**) the length of mapped fragments extracted from SAM file, and (**G**) duplication rates. For (C-G), statistical significance was assessed using two-sided unpaired Welch's t-test. (**H**) Lisa MOTIF analysis using the top 500 genes in young and old (ranked by gene body 5hmC signal). The top 20 young and old TFs (sorted by *p*-value) are labeled. (**I**) Spearman-rank correlation between young (panel 1) and old (panel 2) gene body 5hmC signal and mRNA FC (old vs young). Correlation between old vs young (n=4 each) mean gene body 5hmC signal and mRNA FC between old and young (n=3 each), panel 3. Correlation between random gene body 5hmC values and mRNA FC (old vs young, panel 4). ρ = Spearman's correlation coefficient. *p*-values were derived from Spearman's rank correlation. For all box plots (C-G), the horizontal line within each box represents the 50th, while the bounds of the box depict the 25th and 75th percentile of the data. The whiskers extend to the minima (the smallest value within 1.5 times the IQR below the first quartile, excluding outliers) and the maxima (the largest value within 1.5 times the IQR above the third quartile, excluding outliers). Source data are provided as a Source Data file.

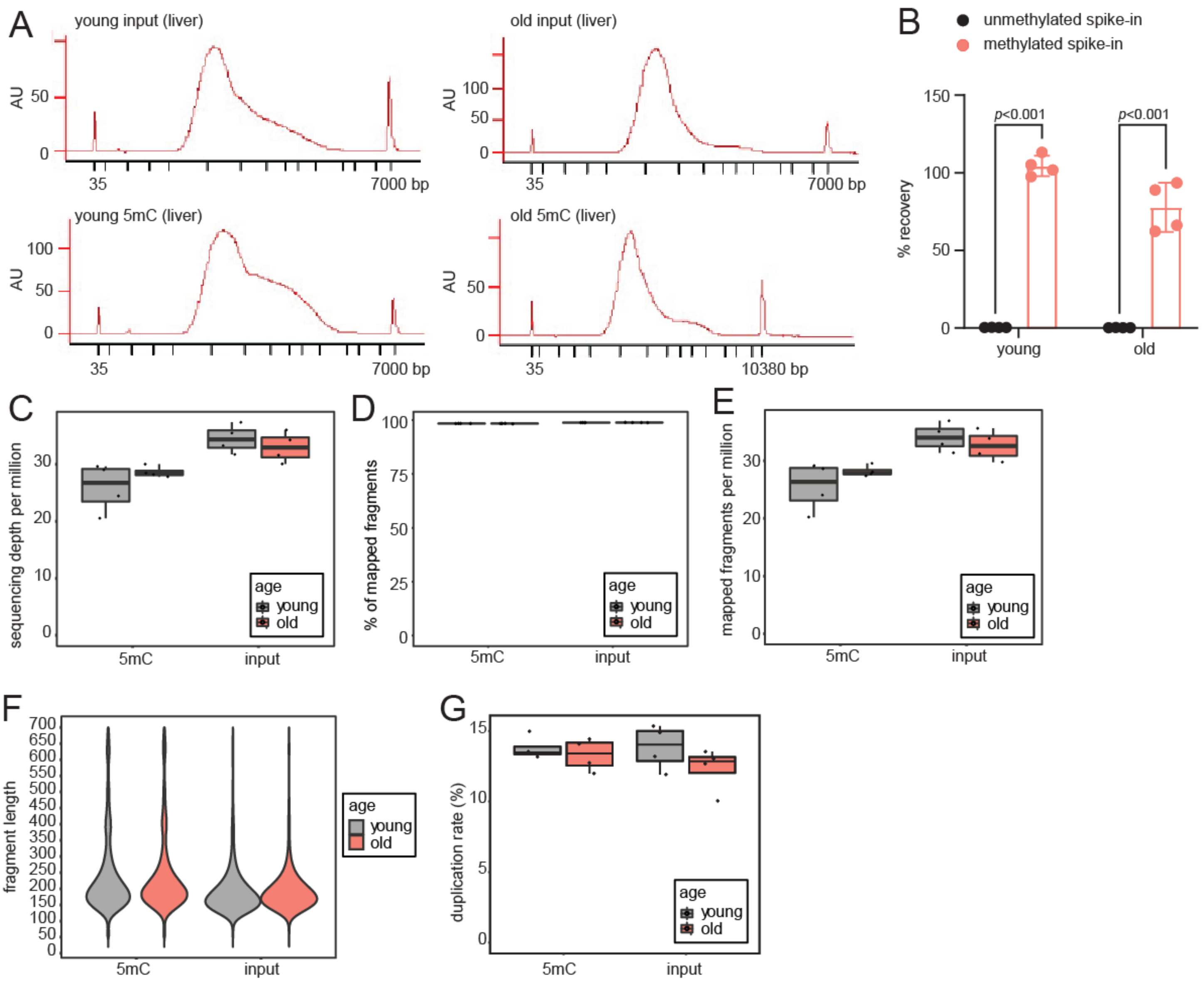
A
young input (liver)
old input (liver)
young 5mC (liver)
old 5mC (liver)
AU
35
7000 bp
10380 bp
B
unmethylated spike-in
methylated spike-in
% recovery
p<0.001
young
old
C
sequencing depth per million
D
% of mapped fragments
E
mapped fragments per million
F
fragment length
G
duplication rate (%)
age
young
old
5mC
input

Supp. Figure 2: Quality control metrics of mouse liver MeDIP-seq libraries

(**A**) (Left) Representative Bio-analyzer electropherogram of liver MeDIP-seq library from young input and 5mC samples (n=4). (Right), same as left but for old liver samples (n=4). AU represents arbitrary fluorescence units. (**B**) qPCR analysis for exogenous unmethylated and hydroxymethylated DNA control spike-ins included in MeDIP-seq for young and old (n=4 each) mouse liver samples. Data are presented as mean ± SD; statistical significance was assessed using a two-way ANOVA with Šídák's multiple comparisons post-hoc test. Figure panels (C-G) present QC data from 5mC and input libraries of young and old (n=4 each) mouse liver samples, including (**C**) sequencing depth, (**D**) percent of alignment rates of 5mC and input libraries to the GRCm38/mm10 genome, (**E**) the number of fragments mapped to the GRCm38/mm10 genome, (**F**) the length of mapped fragments extracted from SAM file, and (**G**) duplication rates. For (C-G), statistical significance was assessed using two-sided unpaired Welch's t-test. For all box plots (C-G), the horizontal line within each box represents the 50th, while the bounds of the box depict the 25th and 75th percentile of the data. The whiskers extend to the minima (the smallest value within 1.5 times the IQR below the first quartile, excluding outliers) and the maxima (the largest value within 1.5 times the IQR above the third quartile, excluding outliers). Source data are provided as a Source Data file.

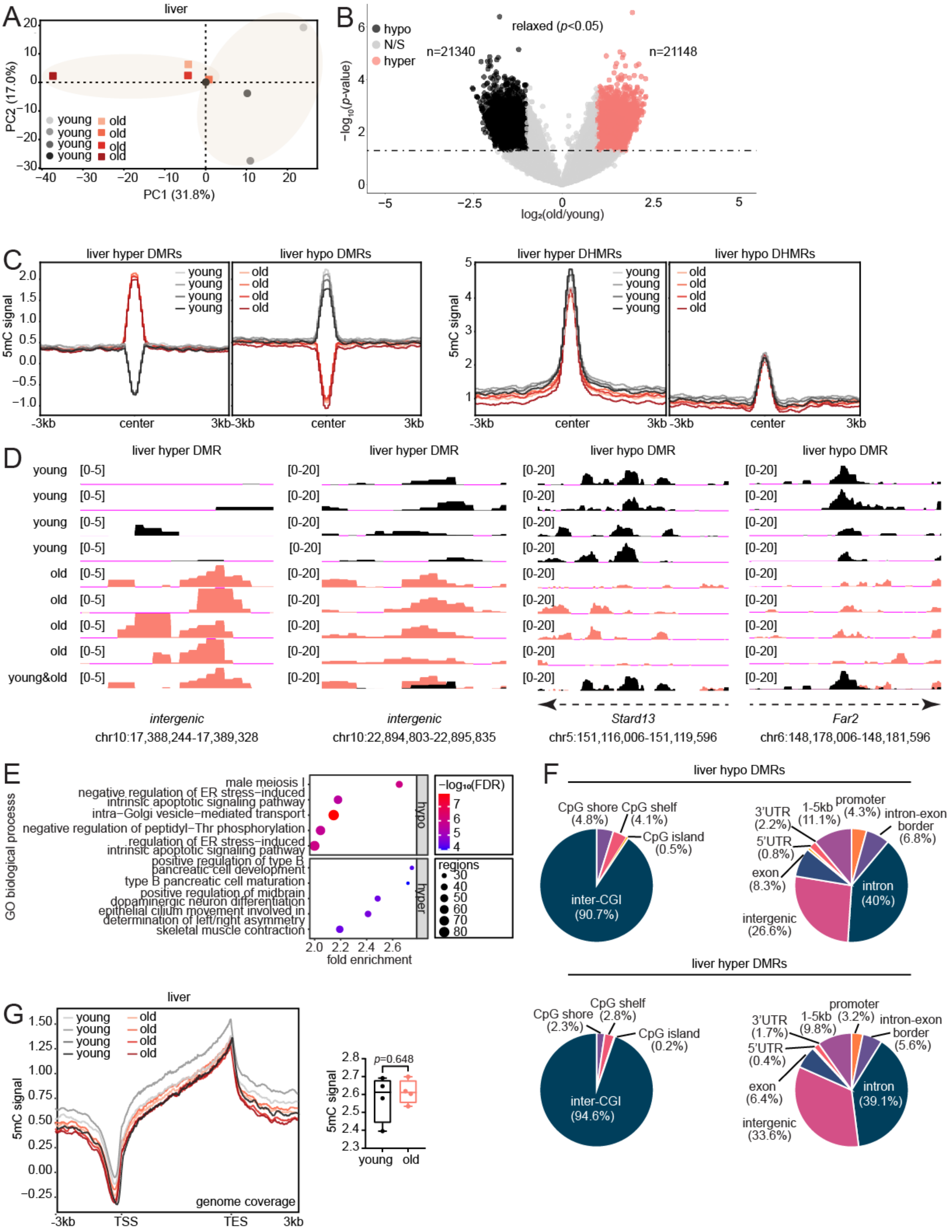
A
liver
PC1 (31.8%)
PC2 (17.0%)
young
old
B
hypo
N/S
hyper
relaxed (p<0.05)
n=21340
n=21148
−log10(p-value)
log2(old/young)
C
liver hyper DMRs
liver hypo DMRs
liver hyper DHMRs
liver hypo DHMRs
5mC signal
-3kb
center
3kb
D
liver hyper DMR
liver hypo DMR
young&old
intergenic
chr10:17,388,244-17,389,328
chr10:22,894,803-22,895,835
Stard13
chr5:151,116,006-151,119,596
Far2
chr6:148,178,006-148,181,596
E
GO biological processs
male meiosis I
negative regulation of ER stress−induced intrinsic apoptotic signaling pathway
intra−Golgi vesicle−mediated transport
negative regulation of peptidyl−Thr phosphorylation
regulation of ER stress−induced intrinsic apoptotic signaling pathway
positive regulation of type B pancreatic cell development
type B pancreatic cell maturation
positive regulation of midbrain dopaminergic neuron differentiation
epithelial cilium movement involved in determination of left/right asymmetry
skeletal muscle contraction
fold enrichment
−log10(FDR)
regions
hypo
hyper
F
liver hypo DMRs
CpG shore (4.8%)
CpG shelf (4.1%)
CpG island (0.5%)
inter-CGI (90.7%)
promoter (4.3%)
1-5kb (11.1%)
3'UTR (2.2%)
5'UTR (0.8%)
exon (8.3%)
intergenic (26.6%)
intron (40%)
intron-exon border (6.8%)
liver hyper DMRs
CpG shore (2.3%)
CpG shelf (2.8%)
CpG island (0.2%)
inter-CGI (94.6%)
promoter (3.2%)
1-5kb (9.8%)
3'UTR (1.7%)
5'UTR (0.4%)
exon (6.4%)
intergenic (33.6%)
intron (39.1%)
intron-exon border (5.6%)
G
liver
genome coverage
-3kb
TSS
TES
3kb
p=0.648
young
old

Supp. Figure 3: Age-related differences in 5hmC occur without detectable differences in 5mC

(**A**) PCA plot obtained using input subtracted 5mC bigWig files of young and old (n=4 each) mouse liver. (**B**) Volcano plot of differentially methylated regions (DMRs) between old and young (n=4 each) mouse liver; identified by QSEA with $p < 0.05$. Hypo DMRs (FC ≤ -2) are regions with less enrichment in the old and hyper DMRs (FC ≥ 2) are regions with higher enrichment in the old. (**C**) Metaplots of young and old (n=4 each) 5mC signal at the DMRs identified by QSEA. (**D**) Example genome browser tracks for liver hyper DMRs (two intergenic regions) and hypo DMRs (*Stard13* and *Far2*). (**E**) GO terms associated with the DMRs from (B) using GREAT. The top 5 biological process terms with FDR < 0.05 are shown. (**F**) Pie charts showing CpG and genic/intergenic annotations of the DMRs from (B). (**G**) Metaplot of young and old (n=4 each) mouse liver 5mC signal over the gene bodies of all mm10 genes; signal quantifications are shown on the side. Statistical significance was assessed using two-sided unpaired Welch's t-test. Source data are provided as a Source Data file.

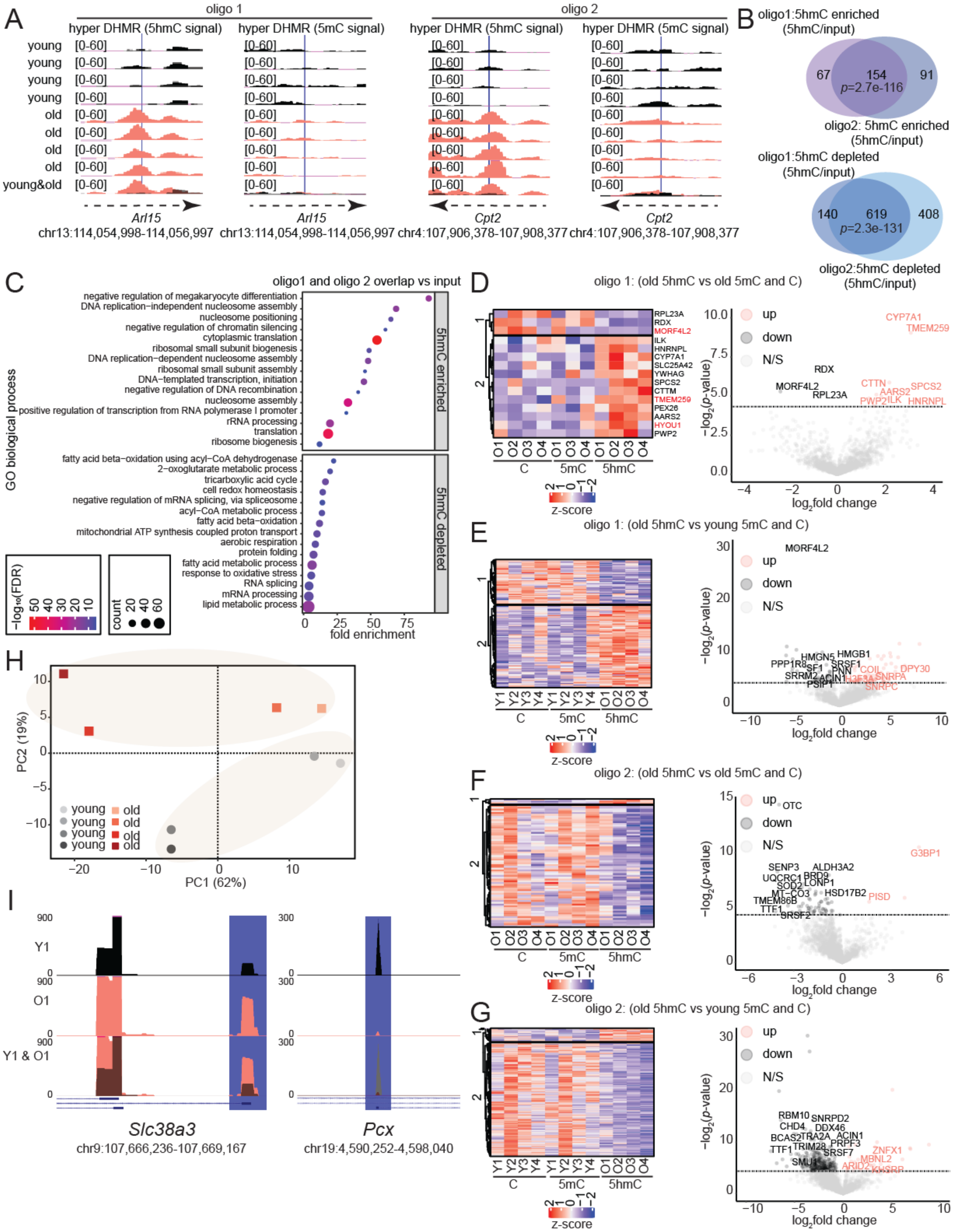
A
oligo 1
oligo 2
hyper DHMR (5hmC signal)
hyper DHMR (5mC signal)
young
old
young&old
[0-60]
Arl15
Cpt2
chr13:114,054,998-114,056,997
chr4:107,906,378-107,908,377
B
oligo1:5hmC enriched (5hmC/input)
67
154
p=2.7e-116
91
oligo2: 5hmC enriched (5hmC/input)
oligo1:5hmC depleted (5hmC/input)
140
619
p=2.3e-131
408
oligo2:5hmC depleted (5hmC/input)
C
oligo1 and oligo 2 overlap vs input
GO biological process
negative regulation of megakaryocyte differentiation
DNA replication−independent nucleosome assembly
nucleosome positioning
negative regulation of chromatin silencing
cytoplasmic translation
ribosomal small subunit biogenesis
DNA replication−dependent nucleosome assembly
ribosomal small subunit assembly
DNA−templated transcription, initiation
negative regulation of DNA recombination
nucleosome assembly
positive regulation of transcription from RNA polymerase I promoter
rRNA processing
translation
ribosome biogenesis
fatty acid beta−oxidation using acyl−CoA dehydrogenase
2−oxoglutarate metabolic process
tricarboxylic acid cycle
cell redox homeostasis
negative regulation of mRNA splicing, via spliceosome
acyl−CoA metabolic process
fatty acid beta−oxidation
mitochondrial ATP synthesis coupled proton transport
aerobic respiration
protein folding
fatty acid metabolic process
response to oxidative stress
RNA splicing
mRNA processing
lipid metabolic process
5hmC enriched
5hmC depleted
−log10(FDR)
count
fold enrichment
D
oligo 1: (old 5hmC vs old 5mC and C)
RPL23A
RDX
MORF4L2
ILK
HNRNPL
CYP7A1
SLC25A42
YWHAG
SPCS2
CTTM
TMEM259
PEX26
AARS2
HYOU1
PWP2
C
5mC
5hmC
z-score
up
down
N/S
−log2(p-value)
log2fold change
E
oligo 1: (old 5hmC vs young 5mC and C)
MORF4L2
HMGN5
HMGB1
PPP1R8
SRSF1
SF1
PNN
SRRM2
ACIN1
PSIP1
COIL
SNRPA
SNRPC
DPY30
F
oligo 2: (old 5hmC vs old 5mC and C)
OTC
G3BP1
SENP3
ALDH3A2
UQCRC1
BRD9
SOD2
LONP1
MT-CO3
HSD17B2
TMEM86B
PISD
TTF1
SRSF2
G
oligo 2: (old 5hmC vs young 5mC and C)
RBM10
SNRPD2
CHD4
DDX46
BCAS2
TRA2A
ACIN1
TTF1
TRIM28
PRPF3
SRSF7
SMU1
ZNFX1
MBNL2
ARID2
KHSRP
H
PC1 (62%)
PC2 (19%)
young
old
I
Y1
O1
Y1 & O1
Slc38a3
chr9:107,666,236-107,669,167
Pcx
chr19:4,590,252-4,598,040

Supp. Figure 4: Supplementary for alternative splicing mediates 5hmC's transcriptionally restrictive function through decreased binding of splicing factors

(**A**) Genome browser views of young and old (n=4 each) 5hmC and 5mC signal at endogenous regions used to design oligo 1 (left) and oligo 2 (right). (**B**) Overlap between oligo 1 and oligo 2 for 5hmC-enriched proteins (top) and input-enriched proteins (bottom) when comparing 5hmC vs input. (**C**) GO analysis using DAVID for proteins overlapping between oligo 1 and oligo 2 in (B). (**D**) Heatmap (left) and volcano plot (right) of differential proteins ($p < 0.05$) in oligo 1 for the comparison of input-subtracted old 5hmC vs old 5mC and C. (**E**) Heatmap (left) and volcano plot (right) of differential proteins ($p < 0.05$) in oligo 1 for the comparison of input-subtracted old 5hmC vs young 5mC and C. (**F**) Heatmap (left) and volcano plot (right) of differential proteins ($p < 0.05$) in oligo 2 for the comparison of input-subtracted old 5hmC vs old 5mC and C. (**G**) Heatmap (left) and volcano plot (right) of differential proteins ($p < 0.05$) in oligo 2 for the comparison of input-subtracted old 5hmC vs young 5mC and C. (**H**) PCA plot of dRNA-seq data based on normalized transcript counts for young and old (n=4 each) mouse liver samples. (**I**) Examples of alternative splicing events in genes with high 5hmC. Shaded area shows a differentially used exon. Source data are provided as a Source Data file.

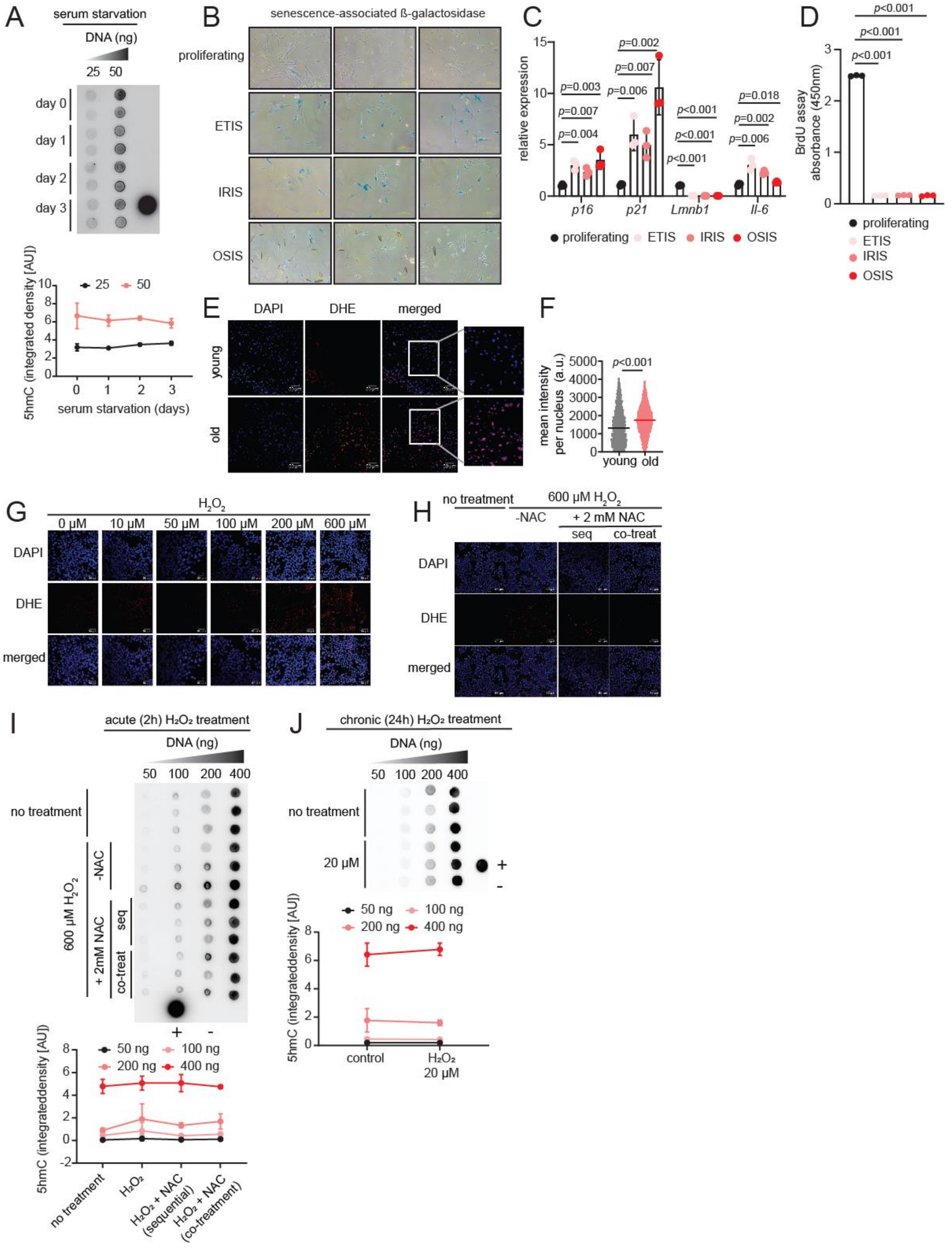
A
serum starvation
DNA (ng)
25 50
day 0
day 1
day 2
day 3
5hmC (integrated density [AU])
25 50
serum starvation (days)
B
senescence-associated ß-galactosidase
proliferating
ETIS
IRIS
OSIS
C
relative expression
p16 p21 Lmnb1 Il-6
p=0.003
p=0.007
p=0.004
p=0.002
p=0.007
p=0.006
p<0.001
p<0.001
p<0.001
p=0.018
p=0.002
p=0.006
proliferating ETIS IRIS OSIS
D
BrdU assay absorbance (450nm)
p<0.001
p<0.001
p<0.001
proliferating
ETIS
IRIS
OSIS
E
DAPI DHE merged
young
old
F
mean intensity per nucleus (a.u.)
p<0.001
young old
G
H2O2
0 µM 10 µM 50 µM 100 µM 200 µM 600 µM
DAPI
DHE
merged
H
no treatment
600 µM H2O2
-NAC
+ 2 mM NAC
seq co-treat
DAPI
DHE
merged
I
acute (2h) H2O2 treatment
DNA (ng)
50 100 200 400
no treatment
600 µM H2O2
-NAC
+ 2mM NAC
seq
co-treat
+ -
5hmC (integrateddensity [AU])
50 ng 100 ng
200 ng 400 ng
no treatment
H2O2
H2O2 + NAC (sequential)
H2O2 + NAC (co-treatment)
J
chronic (24h) H2O2 treatment
DNA (ng)
50 100 200 400
no treatment
20 µM
+
-
50 ng 100 ng
200 ng 400 ng
5hmC (integrateddensity [AU])
control
H2O2 20 µM

Supp. Figure 5: Supplementary for quiescence and senescence drive the increase of 5hmC with age and impact cellular function

(**A**) Dot blot for 5hmC signal using gDNA from serum starvation induced quiescent HepG2 cells; n=2 independent cell cultures sourced from a single vial. + control is 25 ng of young mouse hippocampus gDNA, – control is water. Quantifications are depicted below. Statistical significance was assessed using two-way ANOVA with Tukey's multiple comparisons post-hoc test. **(B)** Representative images of SA-β-gal staining in proliferating and ETIS, IRIS, and OSIS cells. (**C**) qPCR analysis of *p16*, *p21*, *Lmnb1*, and *Il-6* in proliferating and ETIS, IRIS, and OSIS WI-38 cells. For all groups, n=3 independent cell cultures sourced from a single vial; statistical significance was assessed using multiple two-sided unpaired t-test with FDR correction (Benjamini, Krieger, and Yekutieli). (**D**) BrdU assay of proliferating and ETIS, IRIS, and OSIS WI-38 cells. Statistical significance was assessed using multiple two-sided unpaired t-test with FDR correction (Benjamini, Krieger, and Yekutieli). (**E**) Representative images of DHE staining of young and old (n=1 each, male) mouse liver sections. (**F**) DHE mean intensity per nucleus from (E), using data from 5 fields of view for both young and old liver tissue sections. Horizontal bars represent median; statistical significance was assessed using two-sided unpaired Welch's t-test. (**G**) Representative images of DHE staining of HepG2 cells treated with indicated concentrations of H_2O_2 for 2 h. (**H**) Representative images of DHE staining of HepG2 cells treated with 600 µM H_2O_2 for 2 h without NAC, sequential 24 h treatment with NAC after 2 h H_2O_2 treatment, or 2 h co-treatment with NAC followed by 24 h treatment with NAC only. (**I**) 5hmC dot blot of HepG2 cells from (H). Quantifications are depicted below. (**J**) 5hmC dot blot of HepG2 cells treated with H_2O_2 for 24 h. Quantifications are depicted below. For both (I-J), statistical significance was assessed using a two-way ANOVA with Tukey's multiple comparisons post-hoc test. Unless noted, all data are presented as mean ± SD. For (A, I, J), AU represents arbitrary fluorescence units. Source data are provided as a Source Data file.

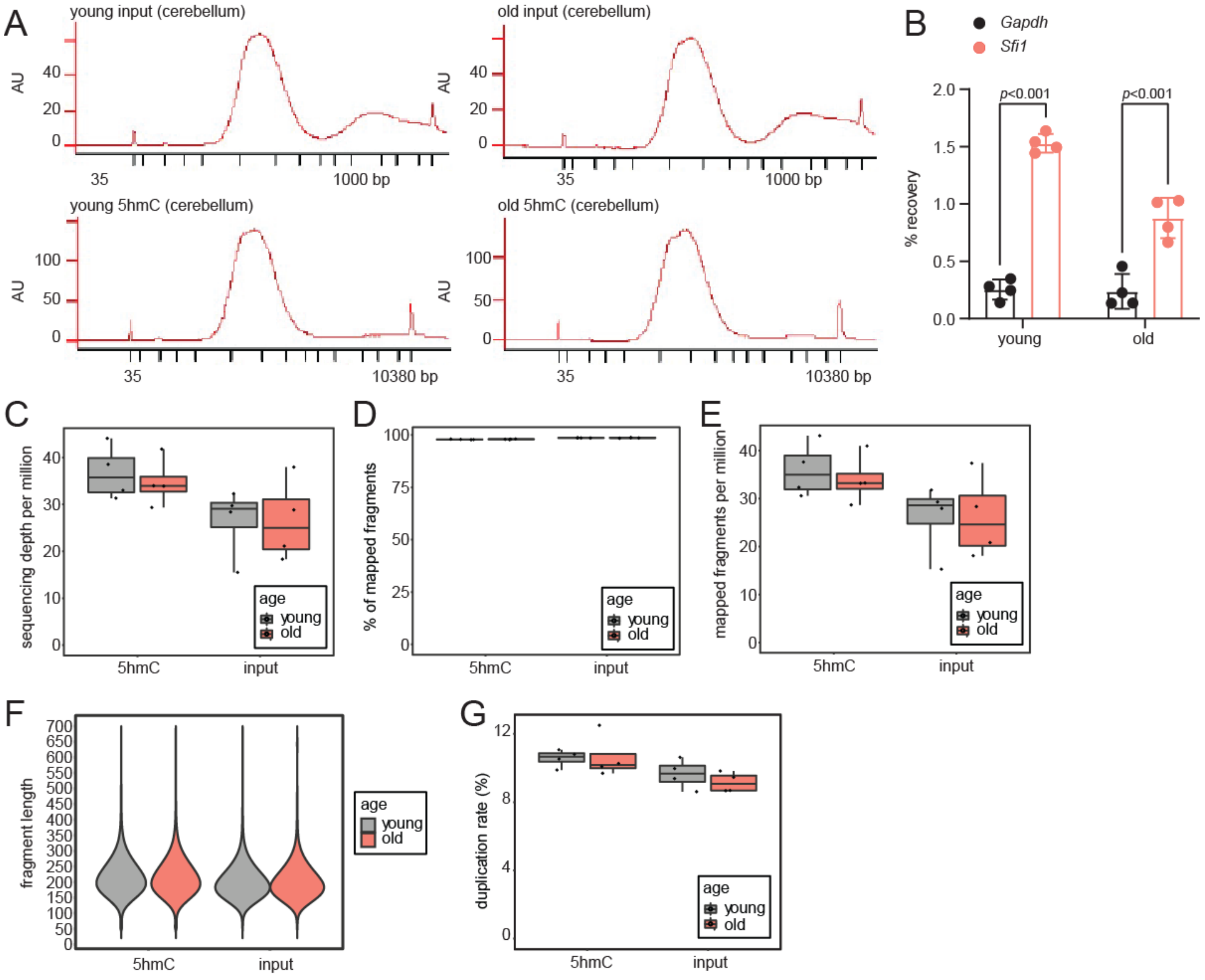
A
young input (cerebellum)
old input (cerebellum)
AU
35
1000 bp
young 5hmC (cerebellum)
old 5hmC (cerebellum)
10380 bp
B
Gapdh
Sfi1
% recovery
p<0.001
young
old
C
sequencing depth per million
age
young
old
5hmC
input
D
% of mapped fragments
E
mapped fragments per million
F
fragment length
G
duplication rate (%)

Supp. Figure 6: Quality control metrics of mouse cerebellum hMeDIP-seq libraries

(**A**) (Left) Representative Bio-analyzer electropherogram of cerebellum hMeDIP-seq library from young input and 5hmC samples (n=4 each). (Right), same as left but for old cerebellum samples (n=4 each). AU represents arbitrary fluorescence units. (**B**) qPCR analysis of endogenous controls, *Gapdh* (negative control) and *Sfi1* (positive control) for young and old (n=4 each) mouse cerebellum samples. Data are presented as mean ± SD; statistical significance was assessed using two-way ANOVA with Šídák's multiple comparisons post-hoc test. Figure panels (C-G) present QC data of 5hmC and input libraries from young and old cerebellum samples, including (**C**) sequencing depth, (**D**) percent of alignment rates of 5hmC and input libraries to the GRCm38/mm10 genome, (**E**) the number of fragments mapped to the GRCm38/mm10 genome, (**F**) the length of mapped fragments extracted from the SAM file, and (**G**) duplication rates. For (C-G), statistical significance was assessed using two-sided unpaired Welch’s t-test. For all box plots (C-G), the horizontal line within each box represents the 50th, while the bounds of the box depict the 25th and 75th percentile of the data. The whiskers extend to the minima (the smallest value within 1.5 times the IQR below the first quartile, excluding outliers) and the maxima (the largest value within 1.5 times the IQR above the third quartile, excluding outliers). Source data are provided as a Source Data file.

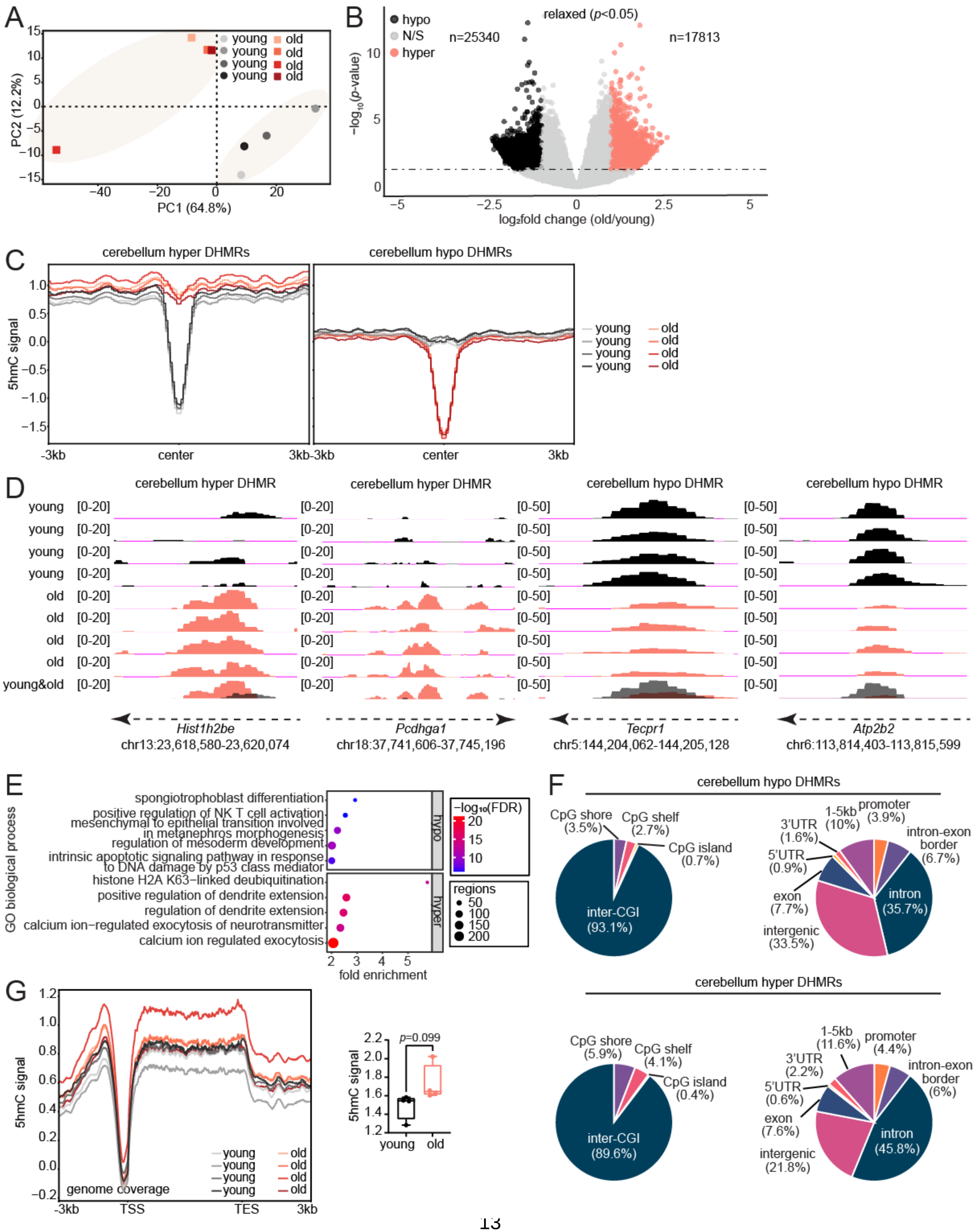
A
PC1 (64.8%)
PC2 (12.2%)
young
old
B
hypo
N/S
hyper
relaxed (p<0.05)
n=25340
n=17813
−log10(p-value)
log2fold change (old/young)
C
cerebellum hyper DHMRs
cerebellum hypo DHMRs
5hmC signal
-3kb
center
3kb
D
cerebellum hyper DHMR
cerebellum hypo DHMR
young&old
Hist1h2be
chr13:23,618,580-23,620,074
Pcdhga1
chr18:37,741,606-37,745,196
Tecpr1
chr5:144,204,062-144,205,128
Atp2b2
chr6:113,814,403-113,815,599
E
GO biological process
spongiotrophoblast differentiation
positive regulation of NK T cell activation
mesenchymal to epithelial transition involved in metanephros morphogenesis
regulation of mesoderm development
intrinsic apoptotic signaling pathway in response to DNA damage by p53 class mediator
histone H2A K63−linked deubiquitination
positive regulation of dendrite extension
regulation of dendrite extension
calcium ion−regulated exocytosis of neurotransmitter
calcium ion regulated exocytosis
hypo
hyper
−log10(FDR)
regions
fold enrichment
F
cerebellum hypo DHMRs
CpG shore (3.5%)
CpG shelf (2.7%)
CpG island (0.7%)
inter-CGI (93.1%)
promoter (3.9%)
1-5kb (10%)
3'UTR (1.6%)
5'UTR (0.9%)
exon (7.7%)
intergenic (33.5%)
intron (35.7%)
intron-exon border (6.7%)
cerebellum hyper DHMRs
CpG shore (5.9%)
CpG shelf (4.1%)
CpG island (0.4%)
inter-CGI (89.6%)
1-5kb (11.6%)
promoter (4.4%)
3'UTR (2.2%)
5'UTR (0.6%)
exon (7.6%)
intergenic (21.8%)
intron (45.8%)
intron-exon border (6%)
G
5hmC signal
genome coverage
-3kb
TSS
TES
3kb
p=0.099
young
old

Supp. Figure 7: 5hmC's transcriptionally restrictive function extends to mouse cerebellum

(**A**) PCA plot obtained using input subtracted 5hmC bigWig files of young and old (n=4 each) mouse liver. (**B**) Volcano plot of DHMRs between old and young (n=4 each) mouse cerebellum; identified by QSEA with $p < 0.05$. Hypo DHMRs (FC ≤ -2) are regions with less enrichment in the old and hyper DHMRs (FC ≥ 2) are regions with higher enrichment in the old. (**C**) Metaplots of young and old (n=4 each) 5hmC signal at the DHMRs in (B). (**D**) Example genome browser tracks for cerebellum hyper DHMRs (*Hist1h2be* and *Pcdhga1*) and hypo DHMRs (*Tecpr1* and *Atp2b2*). (**E**) GO terms associated with the DHMRs from (B) using GREAT. The top 5 biological process terms with FDR < 0.05 are shown. (**F**) Pie charts showing CpG and genic/intergenic annotations of the DHMRs from (B). (**G)** Metaplot of young and old (n=4 each) 5hmC signal over the gene bodies of all mm10 genes; 5hmC signal quantifications are shown on the side. Statistical significance was assessed using two-sided unpaired Welch's t-test. Source data are provided as a Source Data file.

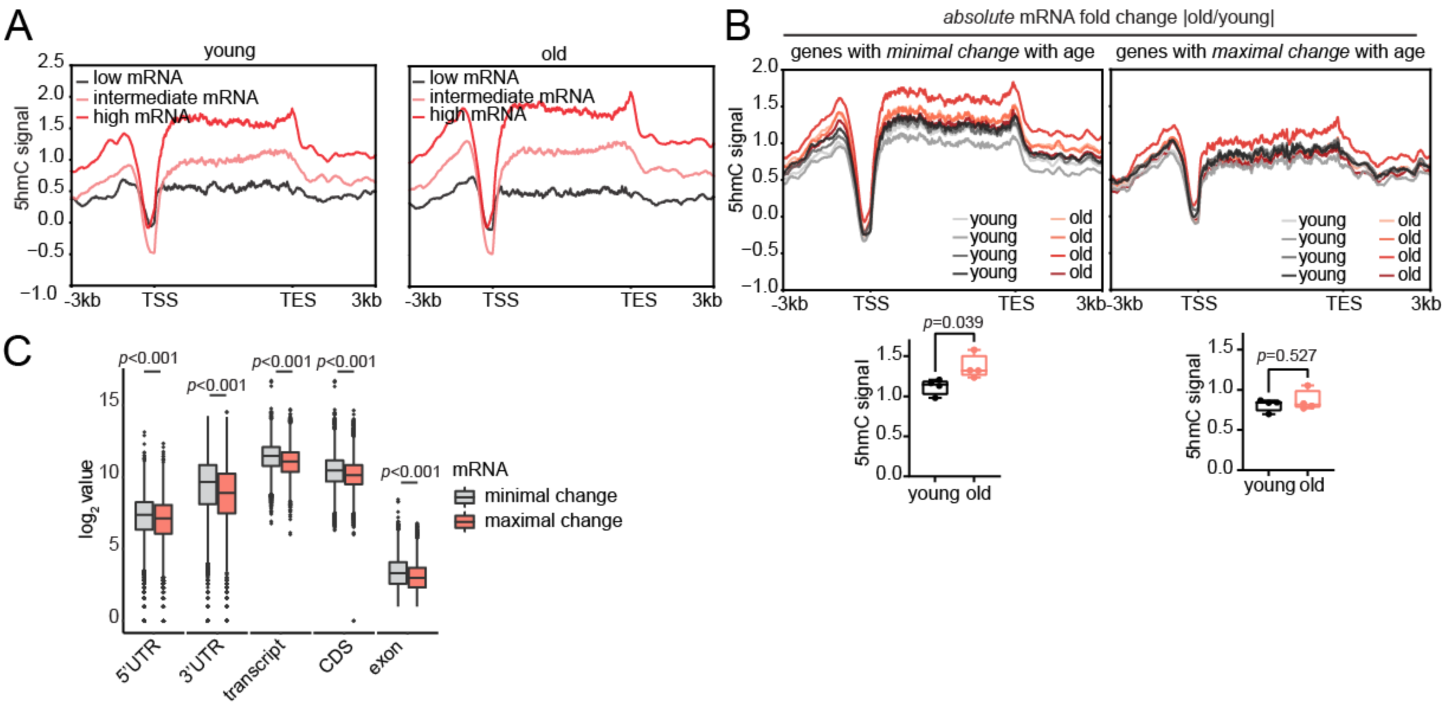
A
young
old
low mRNA
intermediate mRNA
high mRNA
5hmC signal
-3kb
TSS
TES
3kb
B
absolute mRNA fold change |old/young|
genes with minimal change with age
genes with maximal change with age
young
old
p=0.039
p=0.527
C
p<0.001
log2 value
mRNA
minimal change
maximal change
5'UTR
3'UTR
transcript
CDS
exon

Supp. Figure 8: Gene body 5hmC restricts the magnitude of transcriptional changes during cerebellum aging

(**A**) Metaplots of young and old (n=4 each) merged 5hmC signal over the gene bodies with low (n=5900), intermediate (n=5900), and high (n=5900) average mRNA counts for young (left) and old (right) samples (n=4 each). (**B**) Metaplots of young and old (n=4 each) cerebellum 5hmC signal over gene bodies with minimal (left) and maximal (right) expression change between old and young (n=4 each). Quantifications are depicted below; statistical significance was assessed using two-sided unpaired Welch's t-test. (**C**) Box plots showing the distribution of various genic features for the genes with minimal and maximal expression changes between old and young (n=4 each) mice. Statistical significance was assessed using two-sided unpaired Welch's t-test. Source data are provided as a Source Data file.

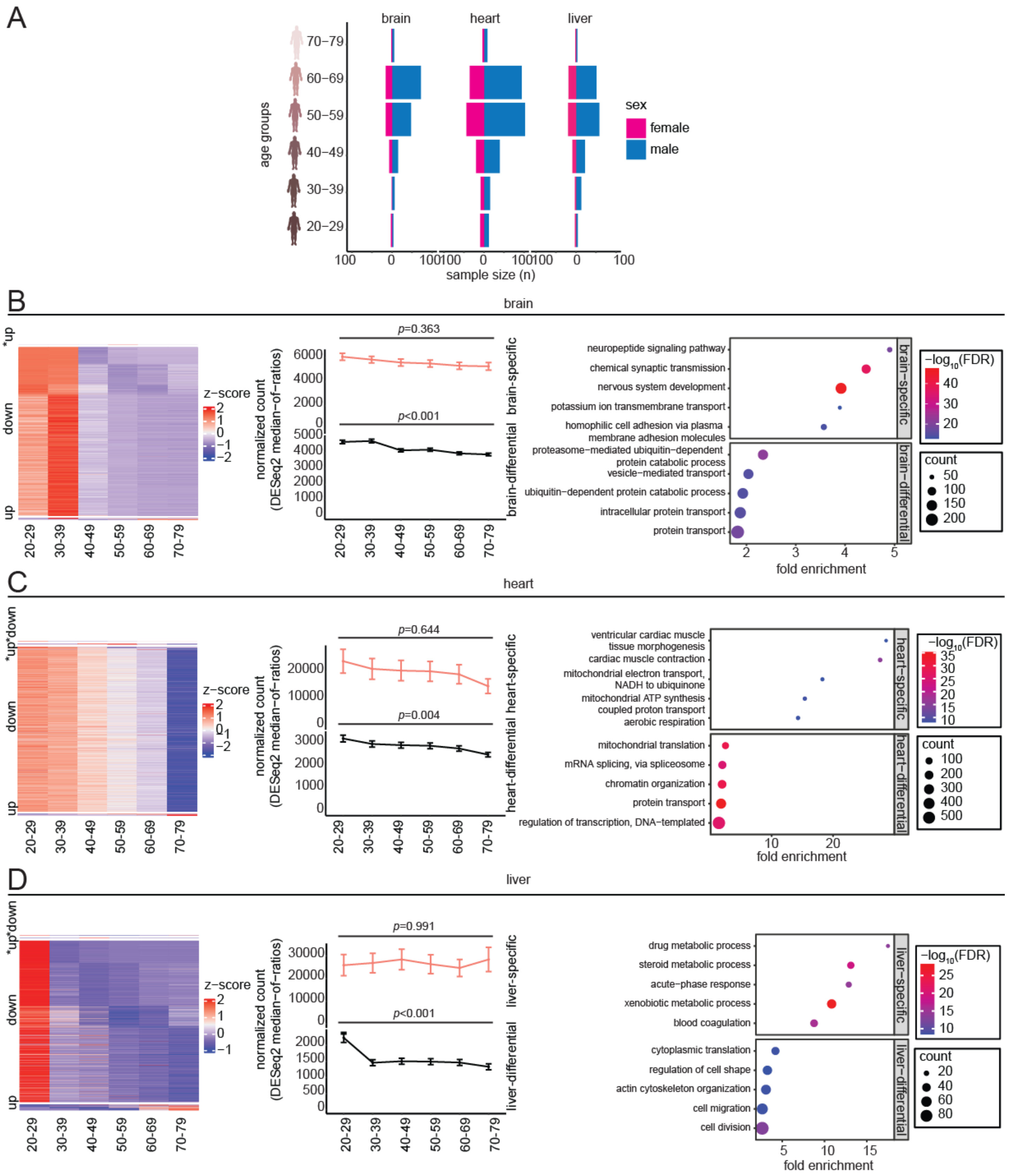

A
brain
heart
liver
70−79
60−69
50−59
40−49
30−39
20−29
age groups
sex
female
male
sample size (n)
B
brain
p=0.363
p<0.001
normalized count (DESeq2 median−of−ratios)
brain-specific
brain-differential
z−score
neuropeptide signaling pathway
chemical synaptic transmission
nervous system development
potassium ion transmembrane transport
homophilic cell adhesion via plasma membrane adhesion molecules
proteasome−mediated ubiquitin−dependent protein catabolic process
vesicle−mediated transport
ubiquitin−dependent protein catabolic process
intracellular protein transport
protein transport
−log10(FDR)
count
fold enrichment
C
heart
p=0.644
p=0.004
heart-specific
heart-differential
ventricular cardiac muscle tissue morphogenesis
cardiac muscle contraction
mitochondrial electron transport, NADH to ubiquinone
mitochondrial ATP synthesis coupled proton transport
aerobic respiration
mitochondrial translation
mRNA splicing, via spliceosome
chromatin organization
protein transport
regulation of transcription, DNA−templated
D
liver
p=0.991
p<0.001
liver-specific
liver-differential
drug metabolic process
steroid metabolic process
acute−phase response
xenobiotic metabolic process
blood coagulation
cytoplasmic translation
regulation of cell shape
actin cytoskeleton organization
cell migration
cell division

Supp. Figure 9: Supplementary for human tissues also show 5hmC-mediated transcriptional restriction

(A) Distribution of sex and age groups among GTEx donors analyzed in this study. (**B**) (Left) Heatmap output from ImpulseDE2 with monotonous and transiently changing genes with age in the brain. (Middle) normalized mRNA count of brain-specific and brain-differential genes from (left). (Right) GO plots associated with the brain-specific and brain-differential genes. (**C**) (Left) Heatmap output from ImpulseDE2 with monotonous and transiently changing genes with age in the heart. (Middle) normalized mRNA count of heart-specific and heart-differential genes from (left). (Right) GO plots associated with the heart-specific and heart-differential genes. (**D**) (Left) Heatmap output from ImpulseDE2 with monotonous and transiently changing genes with age in the liver. (Middle) normalized mRNA count of liver-specific and liver-differential genes from (left). (Right) GO plots associated with the liver-specific and liver-differential genes. Data are presented as mean ± SEM. Statistical significance was assessed using one-way ANOVA test (without post-hoc comparisons). An asterisk (*) indicates transiently downregulated (*down) or upregulated (*up) genes. Terms without asterisks denote monotonously downregulated (down) or upregulated (up) genes. Source data are provided as a Source Data file.

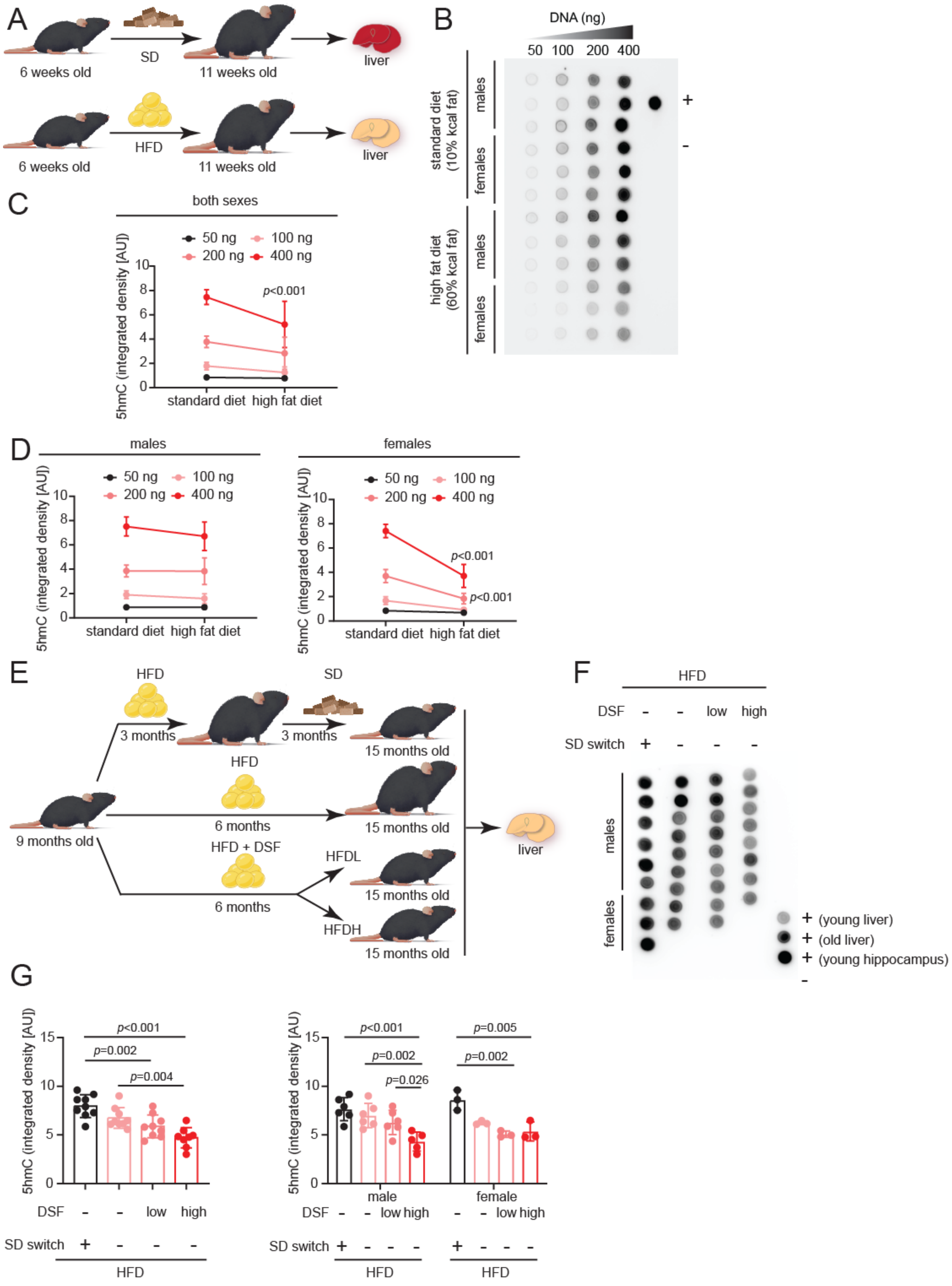
A
6 weeks old
SD
11 weeks old
liver
HFD
B
DNA (ng)
50 100 200 400
standard diet (10% kcal fat)
high fat diet (60% kcal fat)
males
females
+
-
C
both sexes
50 ng
100 ng
200 ng
400 ng
5hmC (integrated density [AU])
p<0.001
standard diet
high fat diet
D
males
females
p<0.001
p<0.001
E
3 months
15 months old
9 months old
6 months
HFD + DSF
HFDL
HFDH
F
DSF
low
high
SD switch
(young liver)
(old liver)
(young hippocampus)
G
p=0.002
p=0.004
male
female
p=0.026
p=0.005

Supp. Figure 10: 5hmC is downregulated in response to high-fat diet and disulfiram

(**A**) Schematic of diet regimen for standard and HFD. (**B**) Dot blot for 5hmC signal in gDNA isolated from mice liver fed on either a SD or HFD (n=6 each). + control is 200 ng of young mouse hippocampus gDNA, – control is water. (**C**) 5hmC quantification from dot blot (B) stratified by type of diet. (**D**) Same as (C) except stratified by type of diet and sex. For (C-D), data are presented as mean ± SD; statistical significance was assessed using two-way ANOVA with Šídák's multiple comparisons post-hoc test. AU represents arbitrary fluorescence units. (**E**) Schematic of diet regimen for HFD and DSF drug treatment. (**F**) Dot blot for 5hmC signal in gDNA isolated from mice liver according to the procedure outlined in (E); 3-month HFD, 3-month SD (n=9), 6-month HFD (n=9), 3-month HFD with low DSF (n=9), and 3-month HFD with high DSF (n=8). (**G**) (Left), quantification of 5hmC signal from (F) stratified by type of diet; statistical significance was assessed using one-way ANOVA with Tukey's multiple comparisons post-hoc test. (Right), same as (left) except stratified by type of diet and sex. Statistical significance was assessed using two-way ANOVA with Tukey's multiple comparisons post-hoc test. For (G, both panels), data are presented as mean ± SD. AU represents arbitrary fluorescence units. Source data are provided as a Source Data file. Illustration credit: Endosymbiont GmbH.
