# Supplementary material for: Gene body DNA hydroxymethylation restricts the magnitude of transcriptional changes during aging
Source: Nat Commun. 2024 Jul 28;15:6357. doi: 10.1038/s41467-024-50725-y (PMC11284234; doi:10.1038/s41467-024-50725-y)
Supplement: Supplementary file 3 — Description of Additional Supplementary Files [file 41467_2024_50725_MOESM3_ESM.pdf]

Description of Additional Supplementary Files

Supp. Dataset 1: Animal information

Sheet 1: DNA mass spectrometry

Sheet 2: Dot blot

Sheet 3: Immunofluorescence microscopy

Sheet 4: Liver hMeDIP and MeDIP

Sheet 5: RNA-seq reported elsewhere 48

Sheet 6: Oligonucleotide mass spectrometry

Sheet 7: Dihydroethidium fluorescence

Sheet 8: direct RNA-seq

Sheet 9: Cerebellum hMeDIP and RNA-seq

Sheet 10: High-fat diet mice obtained from Jackson Labs

Sheet 11: HFD and DSF mice from Bernier et al. 2020

Supp. Dataset 2: DNA mass spectrometry quantification from young and old tissues

Supp. Dataset 3: Liver differentially hydroxymethylated and methylated regions

Sheet 1: Hypo DHMRs (old vs young). Regions identified by QSEA with FC ≤ -2 and FDR < 0.05 in old vs young comparisons. Sheet contains p-values and p-values adjusted for multiple comparisons.

Sheet 2: Hyper DHMRs (old vs young). Regions identified by QSEA with FC ≥ 2 and FDR < 0.05 in old vs

young comparisons. Sheet contains p-values and p-values adjusted for multiple comparisons.

Sheet 3: Hypo DMRs (old vs young). Regions identified by QSEA with FC ≤ -2 and p-value < 0.05 in old vs young comparisons. Sheet contains p-values and p-values adjusted for multiple comparisons.

Sheet 4: Hyper DMRs (old vs young). Regions identified by QSEA with FC ≥ 2 and p-value < 0.05 in old vs young comparisons. Sheet contains p-values and p-values adjusted for multiple comparisons.

Supp. Dataset 4: Liver RNA-seq gene rankings

Sheet 1: Young - low mRNA group (DESeq2 normalized counts)

Sheet 2: Young – intermediate mRNA group (DESeq2 normalized counts)

Sheet 3: Young – high mRNA group (DESeq2 normalized counts)

Sheet 4: Old - low mRNA group (DESeq2 normalized counts)

Sheet 5: Old – intermediate mRNA group (DESeq2 normalized counts)

Sheet 6: Old – high mRNA group (DESeq2 normalized counts)

Sheet 7: Low mRNA FC (old vs young, i.e., downregulated genes). 6340 genes with the lowest FC obtained from DESeq2 differential analysis, ranked by mRNA FC between old and young mice. The sheet includes FC, p-values, and p-values adjusted for multiple comparisons for each gene.

Sheet 8: High mRNA FC (old vs young, i.e., upregulated genes). 6339 genes with the highest FC obtained from DESeq2 differential analysis, ranked by mRNA FC between old and young mice. The sheet includes FC, p-values, and p-values adjusted for multiple comparisons for each gene.

Sheet 9: Low mRNA absolute FC (old vs young, i.e., minimal change genes). 6340 genes with the lowest absolute FC obtained from DESeq2 differential analysis, ranked by absolute mRNA FC between old and young mice. The sheet includes FC, absolute FC, p-values, and p-values adjusted for multiple comparisons for each gene.

Sheet 10: High mRNA absolute FC (old vs young, i.e., maximal change genes). 6339 genes with the highest absolute FC obtained from DESeq2 differential analysis, ranked by absolute mRNA FC between old and young mice. The sheet includes FC, absolute FC, p-values, and p-values adjusted for multiple comparisons for each gene.

Supp. Dataset 5: Oligo mass spectrometry protein quantification

Sheet 1: Raw data

Sheet 2: Processed data

Sheet 3: Simplified data

Supp. Dataset 6: Oligo mass spectrometry differentially enriched proteins

Sheet 1: Oligo 1 - differentially enriched proteins (old 5hmC vs old input)

Sheet 2: Oligo 2 - differentially enriched proteins (old 5hmC vs old input)

Sheet 3: Oligo 1 - differentially enriched proteins (old 5hmC vs old 5mC and C)

Sheet 4: Oligo 1 - differentially enriched proteins (old 5hmC vs young 5mC and C)

Sheet 5: Oligo 2 - differentially enriched proteins (old 5hmC vs old 5mC and C)

Sheet 6: Oligo 2 - differentially enriched proteins (old 5hmC vs young 5mC and C)

Supp. Dataset 7: Alternative splicing results from rMATS

Sheet 1: Skipped exon alternative events – inclusion difference (old vs young)

Sheet 2: Alternative 5’ splice site events – inclusion difference (old vs young)

Sheet 3: Alternative 3’ splice site events – inclusion difference (old vs young)

Sheet 4: Mutually exclusive exon events – inclusion difference (old vs young)

Sheet 5: Retained intron events – inclusion difference (old vs young)

Supp. Dataset 8: Differential isoform usage from direct RNA-seq. Sheet contains results from differential isoform usage analysis performed with DRIMSeq, including gene, p-values, and p-values adjusted for multiple comparisons.

Supp. Dataset 9: Transcript length data using Nanoplen. Differential transcript length analysis using Nanoplen (https://github.com/maragkakislab/nanoplen); sheet contains FC, p-values, q-values, and mean lengths.

Supp. Dataset 10: Cerebellum differentially hydroxymethylated regions and RNA-seq gene rankings

Sheet 1: Hypo DHMRs (old vs young). Regions identified by QSEA with FC $\leq$ -2 and p-value < 0.05 in old vs young comparisons. Sheet contains p-values and p-values adjusted for multiple comparisons.

Sheet 2: Hyper DHMRs (old vs young). Regions identified by QSEA with FC $\geq$ 2 and p-value < 0.05 in old vs young comparisons. Sheet contains p-values and p-values adjusted for multiple comparisons.

Sheet 3: Young - low mRNA group (DESeq2 normalized counts)

Sheet 4: Young – intermediate mRNA group (DESeq2 normalized counts)

Sheet 5: Young – high mRNA group (DESeq2 normalized counts)

Sheet 6: Old - low mRNA group (DESeq2 normalized counts)

Sheet 7: Old – intermediate mRNA group (DESeq2 normalized counts)

Sheet 8: Old – high mRNA group (DESeq2 normalized counts)

Sheet 9: low mRNA absolute FC (old vs young, i.e., minimal change genes). 5900 genes with the lowest absolute FC obtained from DESeq2 differential analysis, ranked by absolute mRNA FC between old and young mice. The sheet includes FC, absolute FC, p-values, and p-values adjusted for multiple comparisons for each gene.

Sheet 10: high mRNA absolute FC (old vs young, i.e., maximal change genes). 5900 genes with the highest absolute FC obtained from DESeq2 differential analysis, ranked by absolute mRNA FC between old and young mice. The sheet includes FC, absolute FC, p-values, and p-values adjusted for multiple comparisons for each gene.

Supp. Dataset 11: GTEx and ImpulseDE2 results

Sheet 1: List of age-related differential genes in brain identified from ImpulseDE2

Sheet 2: List of age-related differential genes in heart identified from ImpulseDE2

Sheet 3: List of age-related differential genes in liver identified from ImpulseDE2

Sheet 4: List of brain-differential and brain-specific genes

Sheet 5: List of heart-differential and heart-specific genes

Sheet 6: List of liver-differential and liver-specific genes

Supp. Dataset 12: Antibodies and DNA oligo sequence

Sheet 1: Antibodies

Sheet 2: DNA oligo sequence used for oligo mass spectrometry
