# Supplementary material for: Gene body DNA hydroxymethylation restricts the magnitude of transcriptional changes during aging
Source: Nat Commun. 2024 Jul 28;15:6357. doi: 10.1038/s41467-024-50725-y (PMC11284234; doi:10.1038/s41467-024-50725-y)
Supplement: Supplementary file 16 — Reporting Summary [file 41467_2024_50725_MOESM16_ESM.pdf]

nature portfolio

Corresponding author(s): Payel Sen

Last updated by author(s): Jun 21, 2024

Reporting Summary

Nature Portfolio wishes to improve the reproducibility of the work that we publish. This form provides structure for consistency and transparency in reporting. For further information on Nature Portfolio policies, see our Editorial Policies and the Editorial Policy Checklist.

Statistics

For all statistical analyses, confirm that the following items are present in the figure legend, table legend, main text, or Methods section.

n/a	Confirmed	
☐	☒	The exact sample size (n) for each experimental group/condition, given as a discrete number and unit of measurement
☐	☒	A statement on whether measurements were taken from distinct samples or whether the same sample was measured repeatedly
☐	☒	The statistical test(s) used AND whether they are one- or two-sided<br>*Only common tests should be described solely by name; describe more complex techniques in the Methods section.*
☐	☒	A description of all covariates tested
☐	☒	A description of any assumptions or corrections, such as tests of normality and adjustment for multiple comparisons
☐	☒	A full description of the statistical parameters including central tendency (e.g. means) or other basic estimates (e.g. regression coefficient) AND variation (e.g. standard deviation) or associated estimates of uncertainty (e.g. confidence intervals)
☐	☒	For null hypothesis testing, the test statistic (e.g. F, t, r) with confidence intervals, effect sizes, degrees of freedom and P value noted<br>*Give P values as exact values whenever suitable.*
☒	☐	For Bayesian analysis, information on the choice of priors and Markov chain Monte Carlo settings
☒	☐	For hierarchical and complex designs, identification of the appropriate level for tests and full reporting of outcomes
☐	☒	Estimates of effect sizes (e.g. Cohen's d, Pearson's r), indicating how they were calculated

Our web collection on statistics for biologists contains articles on many of the points above.

Software and code

Policy information about availability of computer code

Data collection

hMeDIP-seq (liver and cerebellum) and cerebellum RNA-seq samples were sequenced on a NextSeq 2000 platform (Illumina) following manufacturer's protocol. Direct RNA-seq was sequenced on a MinION device (Oxford Nanopore Technologies). Mass spectrometry for cytosine modification samples were run on a nano-liquid chromatography coupled online with tandem mass spectrometry (nLC-MS/MS). Mass spectrometry for DNA oligo pulldown samples were loaded onto a Dionex RSLC Ultimate 300 (Thermo Scientific), coupled online with an Orbitrap Fusion Lumos (Thermo Scientific).

Data analysis

Code used in this study are available via GitHub (https://github.com/PSenlab/Occean_2024) and published in Zenodo (https://zenodo.org/doi/10.5281/zenodo.12167052).
Analysis of hMeDIP and MeDIP-seq (Yang, N. et al., Mol Cell (2023), PMID: 37116496) data:
- Genome alignment: bowtie/2-2.4.4 (mouse reference genome, assembly GRCm38/mm10)
- Generating bigWig files: deeptools/3.5.0
- Genomic annotation: annotatr/1.16.0
- Differential enrichment analysis: QSEA/1.26.0
- AUC calculation: bwtool/1.0
- Motif analysis: LISA (http://lisa.cistrome.org/)
- Gene ontology: GREAT/4.0.4
- Graphical representation: ggplot2/3.3.6 (scatterplots, boxplots, pie charts, and dot plots); GraphPad Prism/9 (barplots); deepTools/3.5.0 (PCA and metaprofile plots); ComplexHeatmap/3.16 (heatmaps).

Analysis of RNA-seq data (this study and Yang, N. et al., Mol Cell (2023), PMID: 37116496) data:
- Genome alignment: STAR/2.7.5b (mouse reference genome, assembly GRCm38/mm10)

- Obtaining gene RPKM counts: Rsubread/2.6.4
- Count normalization and differential gene expression: DESeq2/1.30.1
- Alternative splicing analysis: rmats/4.1.1

Analysis of nanopore direct RNA-seq
- Genome alignment: minimap2/2.24 (mouse reference genome, assembly GRCm38/mm10); basecalled reads (mouse reference transcriptome, Ensembl version 92)
- Identifying and quantifying novel transcripts: FLAIR/v1.7.0
- Differential analysis: DESeq2 (differential expression) and DRIMSeq (differential isoform usage)
- Poly(A) tail length analysis: nanopolish polya package (Workman, R.E. et al., Nat Methods (2019), PMID: 31740818)
- Identifying transcripts with systematic whole-molecule or poly(A) length changes across experimental conditions: in-house scripts using linear mixed models (https://github.com/maragkakislab/nanoplen).

Analysis of mass-spectrometry (cytosine modification)
- Ion chromatogram extraction of C, 5mC and 5hmC for quantification: Skyline software

Analysis of mass-spectrometry (DNA oligo pulldown)
- Search of proteome raw files: Proteome Discoverer software (v2.4, Thermo Scientific) using SEQUEST search engine and the SwissProt mouse database
- Gene ontology: DAVID/6.8
- Hypergeometric test: EVenn (Chen, T. et al., J Genet Genomics (2021), PMID: 34452851)
- Heatmaps: ComplexHeatmap/3.16

Analysis of T24 vitamin C hMeDIP and RNA-seq (Peng, D. et al., Clin Epigenet (2018), PMID: 30005692):
- Genome alignment: bowtie/2-2.4.4 (human reference genome, assembly hg19)
- Generating bigWig files: deeptools/3.5.0
- AUC calculation: bwtool/1.0
- Graphical representation: ggplot2/3.3.6 (boxplots,); GraphPad Prism/9 (barplots); deepTools/3.5.0 (metaprofile plots)

Analysis of publicly available human data:
- Genotype-Tissue Expression (GTEx) RNA-seq analysis: ImpulseDE2/0.99/10
- hmC-CATCH-seq and 5hmC-Seal metaprofile plots: deepTools/3.5.0: deepTools/3.5.0
- Gene ontology: DAVID/6.8
- Heatmaps: ComplexHeatmap/3.16

For manuscripts utilizing custom algorithms or software that are central to the research but not yet described in published literature, software must be made available to editors and reviewers. We strongly encourage code deposition in a community repository (e.g. GitHub). See the Nature Portfolio guidelines for submitting code & software for further information.

Data

Policy information about availability of data

All manuscripts must include a data availability statement. This statement should provide the following information, where applicable:

- Accession codes, unique identifiers, or web links for publicly available datasets
- A description of any restrictions on data availability
- For clinical datasets or third party data, please ensure that the statement adheres to our policy

Liver and cerebellum hMeDIP, cerebellum RNA-seq, and liver direct RNA-seq datasets have been submitted to the Gene Expression Omnibus portal (GEO: GSE221124). Liver RNA-seq and MeDIP data was previously reported in Yang, N. et al., Mol Cell (2023), PMID: 37116496 and available via GEO (GSE185705 and GSE223480, respectively). Raw mass spec data were deposited at chorusproject.org/1795. This study used publicly available data, including human RNA-seq data from GTEx/V8 release (https://gtexportal.org/home/datasets) and hmC-CATCH-seq data (GEO: GSE134078). The processed 5hmC-Seal data (bigWig files) was obtained from study authors (Cui, XL. et al., Nat Commun (2020), PMID: 33268789) and raw data can be obtained from GSE144530. The vitamin C hMeDIP-seq and RNA-seq data for T24 cells were obtained directly from study authors (Peng, D et al., Clin Epigenet (2018), PMID: 30005692).

Research involving human participants, their data, or biological material

Policy information about studies with human participants or human data. See also policy information about sex, gender (identity/presentation), and sexual orientation and race, ethnicity and racism.

Reporting on sex and gender	For the ImpulseDE2 analysis of human GTEx RNA-seq data, information on donors' sex was either self-reported, obtained from family/next of kin, or medical records. Sex was included as a covariate in the analysis and distributions are shown in Supplementary Fig. 9A. Overall, there were 569 male tissue donors (151 liver, 148 brain, and 270 heart) and 224 female tissue donors (56 liver, 45 brain, and 123 heart).
Reporting on race, ethnicity, or other socially relevant groupings	For the secondary data analysis of human GTEx data, information on donors' race, ethnicity, or other socially relevant groupings was not available.
Population characteristics	For the secondary data analysis of human GTEx data, age information was available as 10-year intervals. Age distributions are shown in Supplementary Fig. 9A. The age of most donors for any tissue type was 50-59 or 60-69. Donors that experienced a slow death, defined by death after a long illness with more than 1 day of a terminal phase, were excluded.
Recruitment	This study did not involve participant recruitment.

Ethics oversight	Not relevant to this study.

Note that full information on the approval of the study protocol must also be provided in the manuscript.

Field-specific reporting

Please select the one below that is the best fit for your research. If you are not sure, read the appropriate sections before making your selection.

☒ Life sciences ☐ Behavioural & social sciences ☐ Ecological, evolutionary & environmental sciences

For a reference copy of the document with all sections, see nature.com/documents/nr-reporting-summary-flat.pdf

Life sciences study design

All studies must disclose on these points even when the disclosure is negative.

Sample size	No sample size calculation was performed for the experiments. Sample size for all experiments are depicted in manuscript (n=2-4/condition) and were chosen based on commonly used sample sizes in published aging studies (e.g., Schaum, N. et al., Nature (2020), PMID: 32669715; Maeso-Díaz, R. et al., Aging Cell (2022), PMID: 34984806).
Data exclusions	Sequencing reads overlapping ENCODE blacklisted regions were excluded. For analyses of publicly available human GTEx RNA-seq data, donors that experienced a slow death, defined by death after a long illness with more than 1 day of a terminal phase, were excluded.
Replication	Where possible, all experiments in figures depict data points for individual biological replicates. Findings drawn from liver hMeDIP and RNA-seq data were also replicated in another tissue type (mouse cerebellum; n=4/age group). Mouse data was also replicated using publicly available human data with multiple tissue types.
Randomization	For all experiments, control and test conditions were processed together or in pairs to minimize batch effects.
Blinding	Data collection and analyses were not performed blind to the conditions of the experiments. Samples for each sequencing run experiment were processed and sequenced at the same time to prevent batch effects.

Reporting for specific materials, systems and methods

We require information from authors about some types of materials, experimental systems and methods used in many studies. Here, indicate whether each material, system or method listed is relevant to your study. If you are not sure if a list item applies to your research, read the appropriate section before selecting a response.

Materials & experimental systems

n/a	Involved in the study	
☐	☒	Antibodies
☐	☒	Eukaryotic cell lines
☒	☐	Palaeontology and archaeology
☐	☒	Animals and other organisms
☒	☐	Clinical data
☒	☐	Dual use research of concern
☒	☐	Plants

Methods

n/a	Involved in the study	
☒	☐	ChIP-seq
☒	☐	Flow cytometry
☒	☐	MRI-based neuroimaging

Antibodies

Antibodies used	All antibodies used in this study are listed in Supplementary Table 12.<br>- 5hmC dot blot and immunofluorescence: 5-Hydroxymethylcytosine (5-hmC) antibody (pAb), Active Motif, cat# 39092, RRID# AB_2630381. 1:10,000 dilution (or 0.1 ug/mL) was used for dot blot; 1:500 dilution (or 2 µg/mL) was used for immunofluorescence<br>- hMeDIP-seq: 5-hydroxymethylcytosine (5-hmC) Antibody (mouse), Diagenode, cat# C15200200-50, RRID# Mab-31HMC-050. 1.2 µg of antibody was used per IP for liver; 0.6 µg of antibody was used per IP for cerebellum
Validation	- 5-Hydroxymethylcytosine (5-hmC) antibody (pAb): validated by Active Motif for use in dot blot and IF (validation data: https://www.activemotif.com/catalog/details/39791/5-hydroxymethylcytidine-antibody-pab)<br>- 5-hydroxymethylcytosine (5-hmC) Antibody (mouse): validated by Diagenode for use in hMeDIP-seq (validation data: https://www.diagenode.com/en/p/5-hmc-monoclonal-antibody-mouse-classic-50-ug-50-ul)

Eukaryotic cell lines

Policy information about cell lines and Sex and Gender in Research

Cell line source(s)	HepG2 (ATCC, human male) cells
Authentication	The cell lines were not authenticated.
Mycoplasma contamination	The cell lines were mycoplasma negative.
Commonly misidentified lines (See ICLAC register)	No misidentified cell lines were used.

Animals and other research organisms

Policy information about studies involving animals; ARRIVE guidelines recommended for reporting animal research, and Sex and Gender in Research

Laboratory animals	Male and female C57BL/6JN mice of ~2-5 months and 18-20 months were used in this study to discern age-related differences and acquired from the NIA aged rodent colony. The high-fat diet (HFD) experiment was performed in Jackson Labs and included 6 week old C57BL/6J mice given the diet for 5 weeks until they were 11 weeks old. In disulfiram (DSF) experiments, the mice were fed either a HFD (3 months) then switched to standard diet (3 months), HFD for the entire duration of the study (6 months), HFD with low dosage (100 mg/kg body weight/day) of DSF (HFD-L, 6 months), or HFD with high dosage (200 mg/kg body weight/day) of DSF (HFD-H, 6 months). Mice were sacrificed at 15 months.
Wild animals	This study did not involve wild animals.
Reporting on sex	Overall, our study included approximately equal numbers of male and female mice. Animal sex information for each experiment is provided in Supplementary Table 1. Given the low sample number (n=2 per sex per age group), we have not performed separate sex-based analysis to discern age-related differences. However, in experiments with high-fat diet (HFD) and disulfiram (DSF) (Supp. Fig. 10), where samples numbers were slightly higher, we outline sex-specific differences.
Field-collected samples	The study did not involve field-collected samples.
Ethics oversight	This study was approved by the Animal Care and Use Committee of the NIA in Baltimore, MD under Animal Study Protocol number 481-LGG-2022 (all except disulfiram experiments) and 444-TGB-2016 (disulfiram experiments).

Note that full information on the approval of the study protocol must also be provided in the manuscript.

Plants

Seed stocks	*Report on the source of all seed stocks or other plant material used. If applicable, state the seed stock centre and catalogue number. If plant specimens were collected from the field, describe the collection location, date and sampling procedures.*
Novel plant genotypes	*Describe the methods by which all novel plant genotypes were produced. This includes those generated by transgenic approaches, gene editing, chemical/radiation-based mutagenesis and hybridization. For transgenic lines, describe the transformation method, the number of independent lines analyzed and the generation upon which experiments were performed. For gene-edited lines, describe the editor used, the endogenous sequence targeted for editing, the targeting guide RNA sequence (if applicable) and how the editor was applied.*
Authentication	*Describe any authentication procedures for each seed stock used or novel genotype generated. Describe any experiments used to assess the effect of a mutation and, where applicable, how potential secondary effects (e.g. second site T-DNA insertions, mosiacism, off-target gene editing) were examined.*
