# Supplementary material for: Gene body DNA hydroxymethylation restricts the magnitude of transcriptional changes during aging
Source: Nat Commun. 2024 Jul 28;15:6357. doi: 10.1038/s41467-024-50725-y (PMC11284234; doi:10.1038/s41467-024-50725-y)
Supplement: Supplementary file 17 — Source Data [file 41467_2024_50725_MOESM17_ESM.zip › Source data_revisions_v3/Supp. Fig. 1/Fig.S1A_BioA/2100 expert_High Sensitivity DNA Assay_DEDAE00921_2021-03-22_17-05-26.pdf]

Assay Class:	High Sensitivity DNA Assay	Created:	3/22/2021 5:05:26 PM
Data Path:	C:\...gh Sensitivity DNA Assay_DEDAE00921_2021-03-22_17-05-26.xad	Modified:	3/22/2021 5:53:44 PM

Electrophoresis File Run Summary

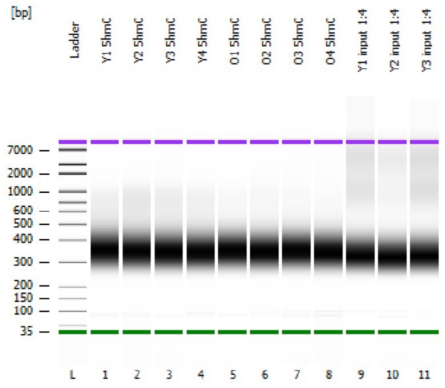

<u>Instrument Information:</u>

Instrument Name:	DEDAE00921	Firmware:	C.01.069
Serial#:	DEDAE00921	Type:	G2939B

<u>Assay Information:</u>

Assay Origin Path:	C:\Program Files (x86)\Agilent\2100 bioanalyzer\2100 expert\assays\dsDNA\High Sensitivity DNA.xsy
Assay Class:	High Sensitivity DNA Assay
Version:	1.03
Assay Comments:	Copyright © 2003-2010 Agilent Technologies

<u>Chip Information:</u>

Chip Lot #:

Reagent Kit Lot #:

Chip Comments:

Y1 5hmC

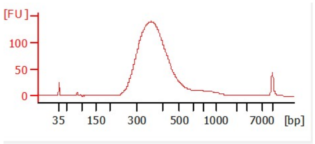

Y2 5hmC

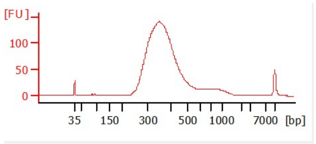

Y3 5hmC

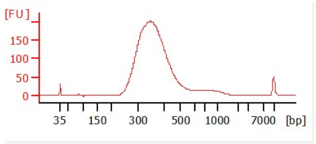

Y4 5hmC

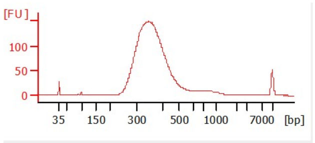

O1 5hmC

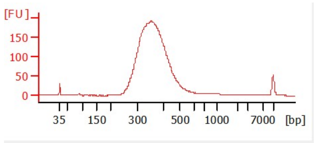

O2 5hmC

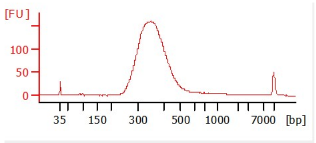

O3 5hmC

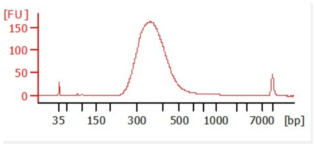

O4 5hmC

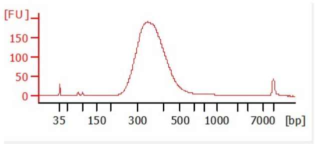

Y1 input 1:4

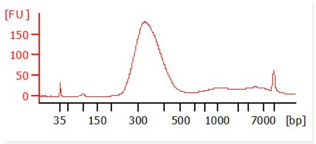

Y2 input 1:4

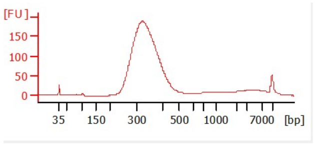

Y3 input 1:4

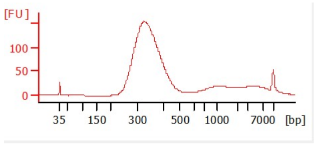

 © Copyright 2003 - 2018 Agilent, Inc.

Assay Class: High Sensitivity DNA Assay
Data Path: C:\...gh Sensitivity DNA Assay_DEDAE00921_2021-03-22_17-05-26.xad
Created: 3/22/2021 5:05:26 PM
Modified: 3/22/2021 5:53:44 PM

Electrophoresis File Run Summary (Chip Summary)

Sample Name	Sample Comment	Rest. Digest	Status	Observation	Result Label	Result Color
Y1 5hmC		☐	✓			
Y2 5hmC		☐	✓			
Y3 5hmC		☐	✓			
Y4 5hmC		☐	✓			
O1 5hmC		☐	✓			
O2 5hmC		☐	✓			
O3 5hmC		☐	✓			
O4 5hmC		☐	✓			
Y1 input 1:4		☐	✓			
Y2 input 1:4		☐	✓			
Y3 input 1:4		☐	✓			
Ladder		☐	✓			

Chip Lot # **Reagent Kit Lot #**

Chip Comments :

 © Copyright 2003 - 2018 Agilent, Inc.

Assay Class: High Sensitivity DNA Assay
Data Path: C:\...gh Sensitivity DNA Assay_DEDAE00921_2021-03-22_17-05-26.xad
Created: 3/22/2021 5:05:26 PM
Modified: 3/22/2021 5:53:44 PM

Electrophoresis Assay Details

General Analysis Settings

Number of Available Sample and Ladder Wells (Max.) : 12
Minimum Visible Range [s] : 32
Maximum Visible Range [s] : 138
Start Analysis Time Range [s] : 33
End Analysis Time Range [s] : 137.5
Ladder Concentration [pg/µl] : 1950
Uses Standard Area for Ladder Fragments
Lower Marker Concentration [pg/µl] : 125
Upper Marker Concentration [pg/µl] : 75
Used Upper Marker for Quantitation
Standard Curve Fit is Point to Point
Show Data Aligned to Lower and Upper Marker

Integrator Settings

Integration Start Time [s] : 33.05
Integration End Time [s] : 137
Slope Threshold : 0.8
Height Threshold [FU] : 5
Area Threshold : 0.1
Width Threshold [s] : 0.6
Baseline Plateau [s] : 0.5

Filter Settings

Filter Width [s] : 0.5
Polynomial Order : 4

Ladder

Ladder Peak	Size	Area
1	35	160
2	50	210
3	100	208
4	150	221
5	200	242
6	300	270
7	400	305
8	500	306
9	600	336
10	700	321
11	1000	366
12	2000	413
13	3000	411
14	7000	400
15	10380	214

© Copyright 2003 - 2018 Agilent, Inc.

Assay Class: High Sensitivity DNA Assay
Data Path: C:\...gh Sensitivity DNA Assay_DEDAE00921_2021-03-22_17-05-26.xad

Created: 3/22/2021 5:05:26 PM
Modified: 3/22/2021 5:53:44 PM

Electropherogram Summary

Ladder

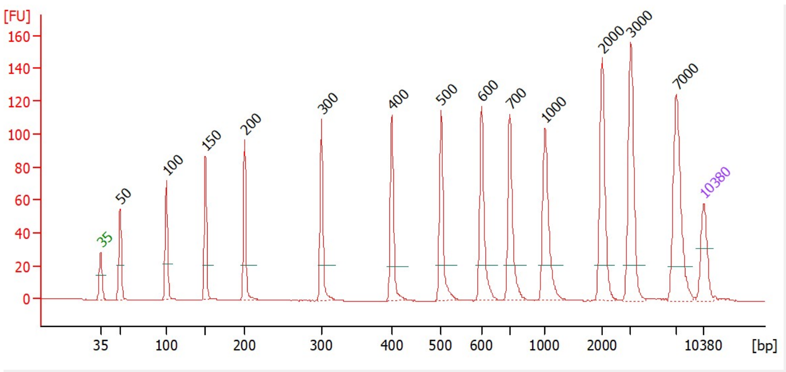

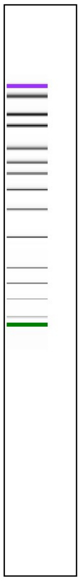

Overall Results for Ladder

Noise: 0.1

Peak table for Ladder

Peak		Size [bp]	Conc. [pg/µl]	Molarity [pmol/l]	Observations
1		35	125.00	5,411.3	Lower Marker
2		50	150.00	4,545.5	Ladder Peak
3		100	150.00	2,272.7	Ladder Peak
4		150	150.00	1,515.2	Ladder Peak
5		200	150.00	1,136.4	Ladder Peak
6		300	150.00	757.6	Ladder Peak
7		400	150.00	568.2	Ladder Peak
8		500	150.00	454.5	Ladder Peak
9		600	150.00	378.8	Ladder Peak
10		700	150.00	324.7	Ladder Peak
11		1,000	150.00	227.3	Ladder Peak
12		2,000	150.00	113.6	Ladder Peak
13		3,000	150.00	75.8	Ladder Peak
14		7,000	150.00	32.5	Ladder Peak
15		10,380	75.00	10.9	Upper Marker

 © Copyright 2003 - 2018 Agilent, Inc.

Assay Class: High Sensitivity DNA Assay
Data Path: C:\...gh Sensitivity DNA Assay_DEDAE00921_2021-03-22_17-05-26.xad
Created: 3/22/2021 5:05:26 PM
Modified: 3/22/2021 5:53:44 PM

Electropherogram Summary Continued ...

Y1 5hmC

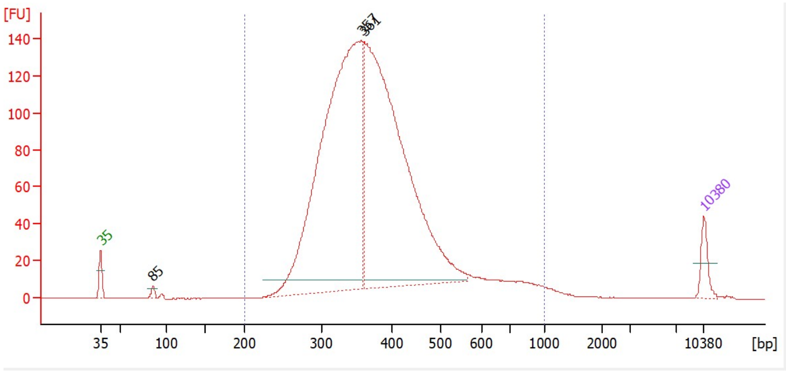

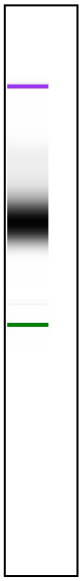

Overall Results for sample 1 : Y1 5hmC

Number of peaks found: 3
Corr. Area 1: 2,347.8
Noise: 0.1

Peak table for sample 1 : Y1 5hmC

Peak		Size [bp]	Conc. [pg/µl]	Molarity [pmol/l]	Observations
1		35	125.00	5,411.3	Lower Marker
2		85	28.57	506.4	
3		357	3,204.91	13,619.8	
4		361	3,117.21	13,075.0	
5		10,380	75.00	10.9	Upper Marker

Region table for sample 1 : Y1 5hmC

From [bp]	To [bp]	Corr. Area	% of Total	Average Size [bp]	Size distribution in CV [%]	Conc. [pg/µl]	Molarity [pmol/l]	Co lor
200	1,000	2,347.8	99	386	25.8	7,154.24	29,890.3	

© Copyright 2003 - 2018 Agilent, Inc.

Assay Class: High Sensitivity DNA Assay
Data Path: C:\...gh Sensitivity DNA Assay_DEDAE00921_2021-03-22_17-05-26.xad

Created: 3/22/2021 5:05:26 PM
Modified: 3/22/2021 5:53:44 PM

Electropherogram Summary Continued ...

Y2 5hmC

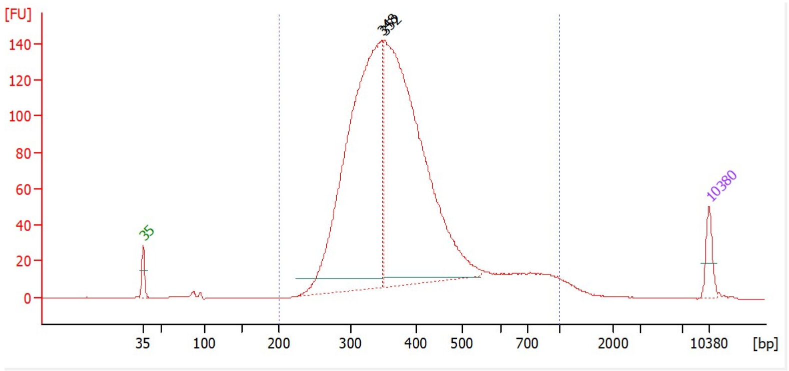

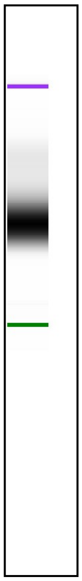

Overall Results for sample 2 : Y2 5hmC

Number of peaks found:	2	Corr. Area 1:	2,431.7
Noise:	0.1		

Peak table for sample 2 : Y2 5hmC

Peak		Size [bp]	Conc. [pg/µl]	Molarity [pmol/l]	Observations
1	◄	35	125.00	5,411.3	Lower Marker
2		348	2,713.51	11,809.7	
3		352	2,864.64	12,320.3	
4	►	10,380	75.00	10.9	Upper Marker

Region table for sample 2 : Y2 5hmC

From [bp]	To [bp]	Corr. Area	% of Total	Average Size [bp]	Size distribution in CV [%]	Conc. [pg/µl]	Molarity [pmol/l]	Co lor
200	1,000	2,431.7	98	389	29.3	6,503.90	27,370.9	

 © Copyright 2003 - 2018 Agilent, Inc.

Assay Class: High Sensitivity DNA Assay
Data Path: C:\...gh Sensitivity DNA Assay_DEDAE00921_2021-03-22_17-05-26.xad
Created: 3/22/2021 5:05:26 PM
Modified: 3/22/2021 5:53:44 PM

Electropherogram Summary Continued ...

Y3 5hmC

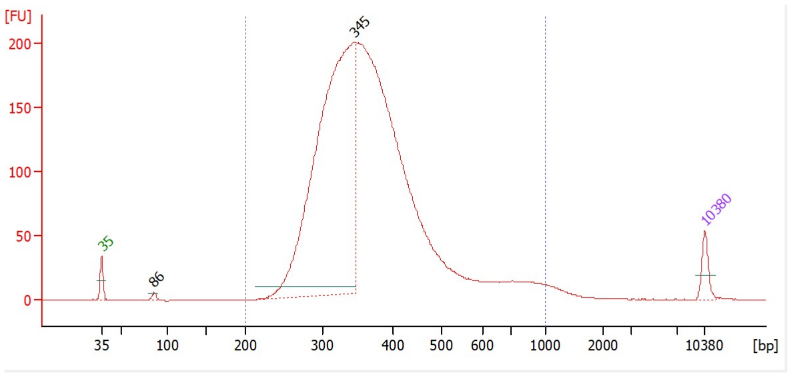

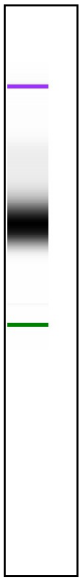

Overall Results for sample 3 : Y3 5hmC

Number of peaks found:	2	Corr. Area 1:	3,393.6
Noise:	0.1		

Peak table for sample 3 : Y3 5hmC

Peak		Size [bp]	Conc. [pg/µl]	Molarity [pmol/l]	Observations
1	◀	35	125.00	5,411.3	Lower Marker
2		86	21.59	382.4	
3		345	3,830.71	16,834.0	
4	▶	10,380	75.00	10.9	Upper Marker

Region table for sample 3 : Y3 5hmC

From [bp]	To [bp]	Corr. Area	% of Total	Average Size [bp]	Size distribution in CV [%]	Conc. [pg/µl]	Molarity [pmol/l]	Color
200	1,000	3,393.6	99	380	27.3	8,764.82	37,348.5	■

 © Copyright 2003 - 2018 Agilent, Inc.

Assay Class:	High Sensitivity DNA Assay	Created:	3/22/2021 5:05:26 PM
Data Path:	C:\...gh Sensitivity DNA Assay_DEDAE00921_2021-03-22_17-05-26.xad	Modified:	3/22/2021 5:53:44 PM

Electropherogram Summary Continued ...

Y4 5hmC

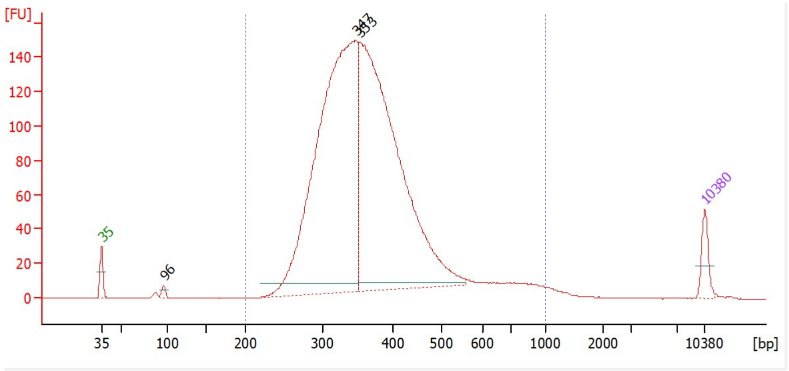

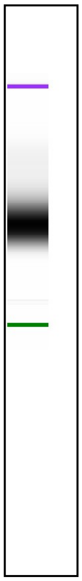

Overall Results for sample 4 : Y4 5hmC

Number of peaks found:	3	Corr. Area 1:	2,457.4
Noise:	0.1		

Peak table for sample 4 : Y4 5hmC

Peak		Size [bp]	Conc. [pg/µl]	Molarity [pmol/l]	Observations
1	◀	35	125.00	5,411.3	Lower Marker
2		96	28.00	440.5	
3		347	2,983.39	13,015.2	
4		353	2,879.82	12,369.2	
5	▶	10,380	75.00	10.9	Upper Marker

Region table for sample 4 : Y4 5hmC

From [bp]	To [bp]	Corr. Area	% of Total	Average Size [bp]	Size distribution in CV [%]	Conc. [pg/µl]	Molarity [pmol/l]	Co lor
200	1,000	2,457.4	98	376	25.9	6,462.73	27,692.3	■

 © Copyright 2003 - 2018 Agilent, Inc.

Assay Class: High Sensitivity DNA Assay
Data Path: C:\...gh Sensitivity DNA Assay_DEDAE00921_2021-03-22_17-05-26.xad
Created: 3/22/2021 5:05:26 PM
Modified: 3/22/2021 5:53:44 PM

Electropherogram Summary Continued ...

O1 5hmC

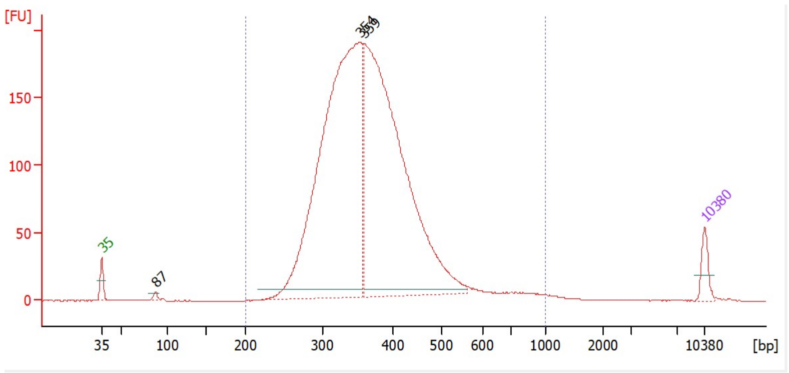

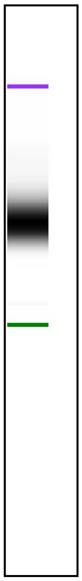

Overall Results for sample 5 : O1 5hmC

Number of peaks found:	3	Corr. Area 1:	3,019.4
Noise:	0.1		

Peak table for sample 5 : O1 5hmC

Peak		Size [bp]	Conc. [pg/µl]	Molarity [pmol/l]	Observations
1	◀	35	125.00	5,411.3	Lower Marker
2		87	24.96	433.6	
3		354	3,705.96	15,879.4	
4		359	3,540.82	14,959.7	
5	▶	10,380	75.00	10.9	Upper Marker

Region table for sample 5 : O1 5hmC

From [bp]	To [bp]	Corr. Area	% of Total	Average Size [bp]	Size distribution in CV [%]	Conc. [pg/µl]	Molarity [pmol/l]	Co lor
200	1,000	3,019.4	99	373	21.8	7,711.44	32,811.5	

 © Copyright 2003 - 2018 Agilent, Inc.

Assay Class: High Sensitivity DNA Assay
Data Path: C:\...gh Sensitivity DNA Assay_DEDAE00921_2021-03-22_17-05-26.xad

Created: 3/22/2021 5:05:26 PM
Modified: 3/22/2021 5:53:44 PM

Electropherogram Summary Continued ...

O2 5hmC

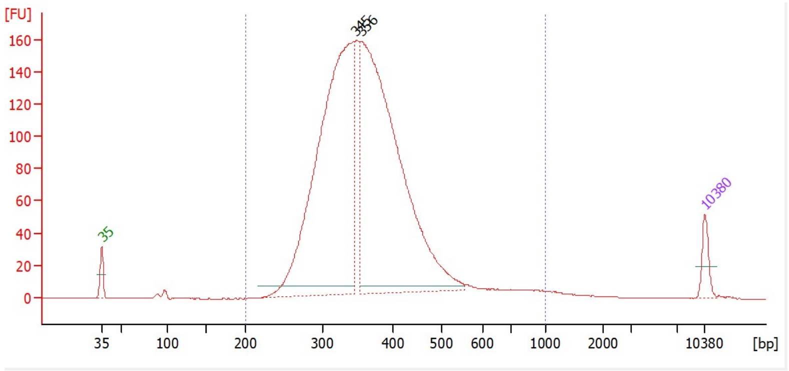

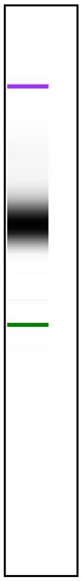

Overall Results for sample 6 : **O2 5hmC**

Number of peaks found: 2

Corr. Area 1: 2,506.5

Noise: 0.1

Peak table for sample 6 : **O2 5hmC**

Peak		Size [bp]	Conc. [pg/µl]	Molarity [pmol/l]	Observations
1	◀	35	125.00	5,411.3	Lower Marker
2		345	2,979.17	13,082.2	
3		356	3,139.53	13,370.1	
4	▶	10,380	75.00	10.9	Upper Marker

Region table for sample 6 : **O2 5hmC**

From [bp]	To [bp]	Corr. Area	% of Total	Average Size [bp]	Size distribution in CV [%]	Conc. [pg/µl]	Molarity [pmol/l]	Color
200	1,000	2,506.5	98	370	22.8	6,946.97	29,893.8	■

 © Copyright 2003 - 2018 Agilent, Inc.

Assay Class: High Sensitivity DNA Assay
Data Path: C:\...gh Sensitivity DNA Assay_DEDAE00921_2021-03-22_17-05-26.xad

Created: 3/22/2021 5:05:26 PM
Modified: 3/22/2021 5:53:44 PM

Electropherogram Summary Continued ...

O3 5hmC

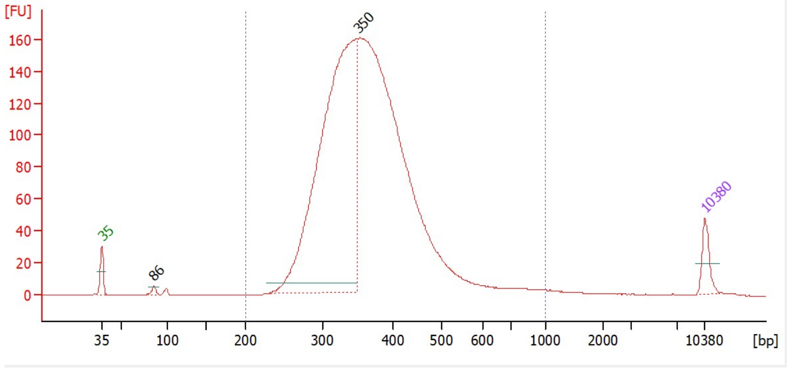

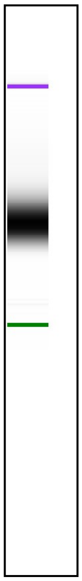

Overall Results for sample 7 : O3 5hmC

Number of peaks found:	2	Corr. Area 1:	2,530.3
Noise:	0.1		

Peak table for sample 7 : O3 5hmC

Peak		Size [bp]	Conc. [pg/µl]	Molarity [pmol/l]	Observations
1	◀	35	125.00	5,411.3	Lower Marker
2		86	22.35	395.4	
3		350	2,988.96	12,956.8	
4	▶	10,380	75.00	10.9	Upper Marker

Region table for sample 7 : O3 5hmC

From [bp]	To [bp]	Corr. Area	% of Total	Average Size [bp]	Size distribution in CV [%]	Conc. [pg/µl]	Molarity [pmol/l]	Color
200	1,000	2,530.3	99	372	21.2	6,880.74	29,299.8	

 © Copyright 2003 - 2018 Agilent, Inc.

Assay Class: High Sensitivity DNA Assay
Data Path: C:\...gh Sensitivity DNA Assay_DEDAE00921_2021-03-22_17-05-26.xad

Created: 3/22/2021 5:05:26 PM
Modified: 3/22/2021 5:53:44 PM

Electropherogram Summary Continued ...

O4 5hmC

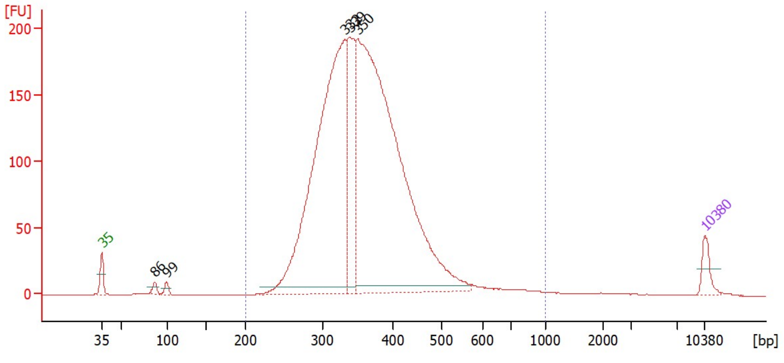

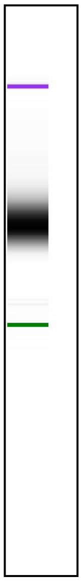

Overall Results for sample 8 : O4 5hmC

Number of peaks found: 5
Corr. Area 1: 3,021.2
Noise: 0.1

Peak table for sample 8 : O4 5hmC

Peak		Size [bp]	Conc. [pg/µl]	Molarity [pmol/l]	Observations
1	◀	35	125.00	5,411.3	Lower Marker
2		86	40.66	713.2	
3		99	36.93	562.7	
4		332	3,053.83	13,938.5	
5		339	795.55	3,557.3	
6		350	4,175.52	18,059.3	
7	▶	10,380	75.00	10.9	Upper Marker

Region table for sample 8 : O4 5hmC

From [bp]	To [bp]	Corr. Area	% of Total	Average Size [bp]	Size distribution in CV [%]	Conc. [pg/µl]	Molarity [pmol/l]	Co lor
200	1,000	3,021.2	98	365	20.6	8,139.42	35,272.9	■

© Copyright 2003 - 2018 Agilent, Inc.

Assay Class: High Sensitivity DNA Assay
Data Path: C:\...gh Sensitivity DNA Assay_DEDAE00921_2021-03-22_17-05-26.xad

Created: 3/22/2021 5:05:26 PM
Modified: 3/22/2021 5:53:44 PM

Electropherogram Summary Continued ...

Y1 input 1:4

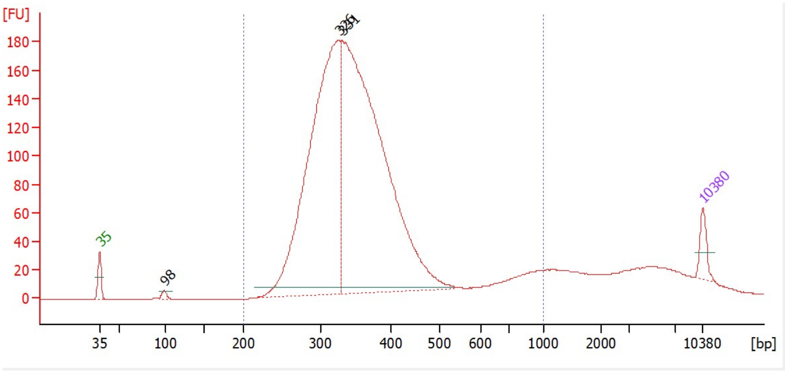

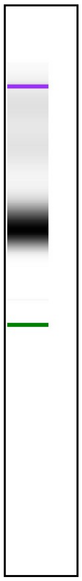

Overall Results for sample 9 : Y1 input 1:4

Number of peaks found: 3

Corr. Area 1: 2,624.1

Noise: 0.1

Peak table for sample 9 : Y1 input 1:4

Peak		Size [bp]	Conc. [pg/µl]	Molarity [pmol/l]	Observations
1	◀	35	125.00	5,411.3	Lower Marker
2		98	28.82	443.9	
3		326	3,035.12	14,111.3	
4		331	4,036.40	18,474.6	
5	▶	10,380	75.00	10.9	Upper Marker

Region table for sample 9 : Y1 input 1:4

From [bp]	To [bp]	Corr. Area	% of Total	Average Size [bp]	Size distribution in CV [%]	Conc. [pg/µl]	Molarity [pmol/l]	Co lor
200	1,000	2,624.1	90	367	30.1	7,351.66	32,536.8	

© Copyright 2003 - 2018 Agilent, Inc.

Assay Class: High Sensitivity DNA Assay
Data Path: C:\...gh Sensitivity DNA Assay_DEDAE00921_2021-03-22_17-05-26.xad

Created: 3/22/2021 5:05:26 PM
Modified: 3/22/2021 5:53:44 PM

Electropherogram Summary Continued ...

Y2 input 1:4

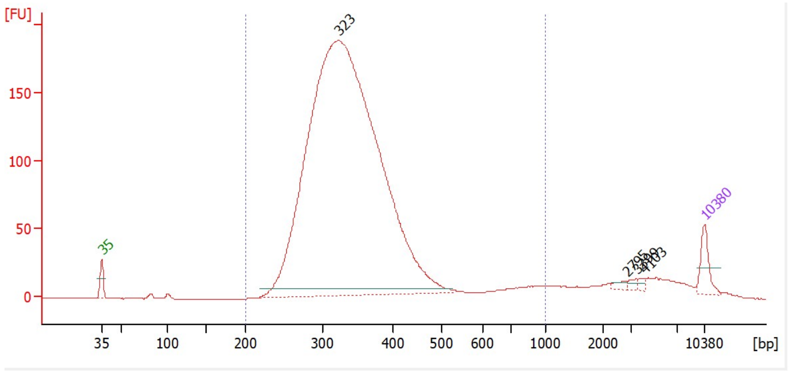

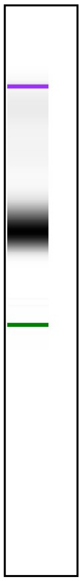

Overall Results for sample 10 : Y2 input 1:4

Number of peaks found: 4

Corr. Area 1: 2,698.3

Noise: 0.2

Peak table for sample 10 : Y2 input 1:4

Peak		Size [bp]	Conc. [pg/µl]	Molarity [pmol/l]	Observations
1	◀	35	125.00	5,411.3	Lower Marker
2		323	5,881.16	27,594.3	
3		2,795	14.45	7.8	
4		3,399	12.80	5.7	
5		4,103	11.34	4.2	
6	▶	10,380	75.00	10.9	Upper Marker

Region table for sample 10 : Y2 input 1:4

From [bp]	To [bp]	Corr. Area	% of Total	Average Size [bp]	Size distribution in CV [%]	Conc. [pg/µl]	Molarity [pmol/l]	Color
200	1,000	2,698.3	94	349	25.5	5,994.20	27,382.1	

© Copyright 2003 - 2018 Agilent, Inc.

Assay Class:	High Sensitivity DNA Assay	Created:	3/22/2021 5:05:26 PM
Data Path:	C:\...gh Sensitivity DNA Assay_DEDAE00921_2021-03-22_17-05-26.xad	Modified:	3/22/2021 5:53:44 PM

Electropherogram Summary Continued ...

Y3 input 1:4

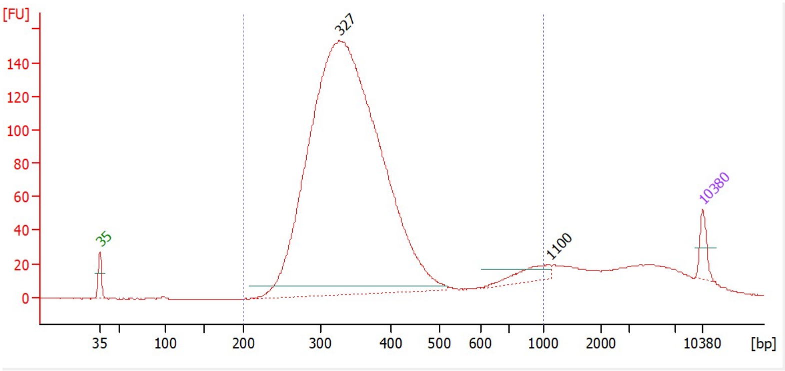

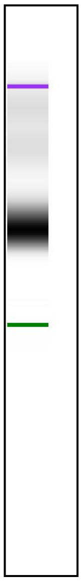

Overall Results for sample 11 : Y3 input 1:4

Number of peaks found:	2	Corr. Area 1:	2,226.3
Noise:	0.1		

Peak table for sample 11 : Y3 input 1:4

Peak	Size [bp]	Conc. [pg/µl]	Molarity [pmol/l]	Observations
1	35	125.00	5,411.3	Lower Marker
2	327	7,264.24	33,690.3	
3	1,100	117.18	161.4	
4	10,380	75.00	10.9	Upper Marker

Region table for sample 11 : Y3 input 1:4

From [bp]	To [bp]	Corr. Area	% of Total	Average Size [bp]	Size distribution in CV [%]	Conc. [pg/µl]	Molarity [pmol/l]	Co lor
200	1,000	2,226.3	89	369	32.6	7,576.58	33,644.3	

 © Copyright 2003 - 2018 Agilent, Inc.

Assay Class:	High Sensitivity DNA Assay	Created:	3/22/2021 5:05:26 PM
Data Path:	C:\...gh Sensitivity DNA Assay_DEDAE00921_2021-03-22_17-05-26.xad	Modified:	3/22/2021 5:53:44 PM

Gel Image

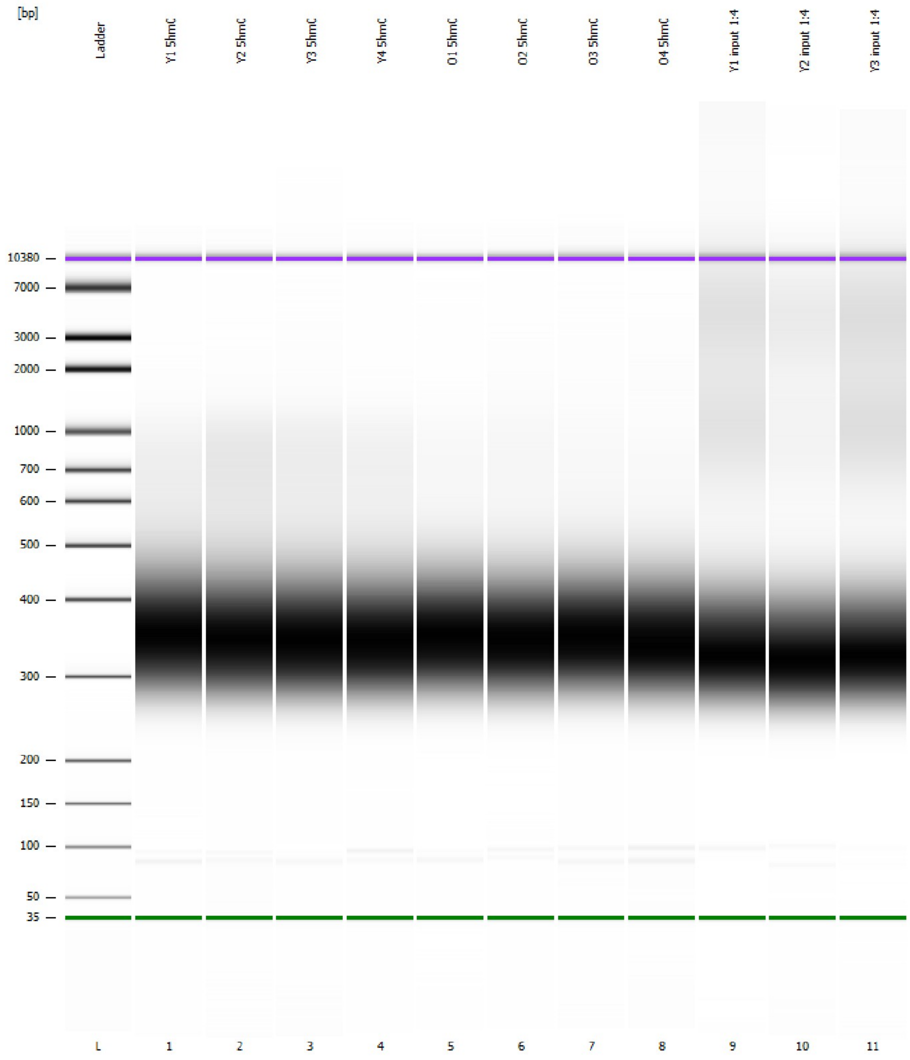

 © Copyright 2003 - 2018 Agilent, Inc.

Assay Class: High Sensitivity DNA Assay
Data Path: C:\...gh Sensitivity DNA Assay_DEDAE00921_2021-03-22_17-05-26.xad
Created: 3/22/2021 5:05:26 PM
Modified: 3/22/2021 5:53:44 PM

Run Logbook

Description	Number	Source	Category	Sub Category	Time	Time Zone	User	Host
Run ended on port 3 (Number of wells acquired: 12)		Instrument	Run		3/22/2021 5:46:44 PM	(GMT --04:00) Eastern Standard Time	senlab	NIALGG-02162892
Run started on port 3 (File: C:\Program Files (x86)\Agilent\2100 bioanalyzer\2100 expert\Data\2021-03-22\2100 expert_High Sensitivity DNA Assay_DEDAE00921_2021-03-22_17-05-26.xad)		Instrument	Run		3/22/2021 5:05:32 PM	(GMT --04:00) Eastern Standard Time	senlab	NIALGG-02162892
Product Number : G2939B		Instrument	Run		3/22/2021 5:05:32 PM	(GMT --04:00) Eastern Standard Time	senlab	NIALGG-02162892
Name :		Instrument	Run		3/22/2021 5:05:32 PM	(GMT --04:00) Eastern Standard Time	senlab	NIALGG-02162892
Vendor : Agilent Technologies		Instrument	Run		3/22/2021 5:05:32 PM	(GMT --04:00) Eastern Standard Time	senlab	NIALGG-02162892
Serial# : DEDAE00921		Instrument	Run		3/22/2021 5:05:32 PM	(GMT --04:00) Eastern Standard Time	senlab	NIALGG-02162892
Firmware : C.01.069		Instrument	Run		3/22/2021 5:05:32 PM	(GMT --04:00) Eastern Standard Time	senlab	NIALGG-02162892
Cartridge : Electrode		Instrument	Run		3/22/2021 5:05:32 PM	(GMT --04:00) Eastern Standard Time	senlab	NIALGG-02162892

© Copyright 2003 - 2018 Agilent, Inc.
