# Supplementary material for: Gene body DNA hydroxymethylation restricts the magnitude of transcriptional changes during aging
Source: Nat Commun. 2024 Jul 28;15:6357. doi: 10.1038/s41467-024-50725-y (PMC11284234; doi:10.1038/s41467-024-50725-y)
Supplement: Supplementary file 17 — Source Data [file 41467_2024_50725_MOESM17_ESM.zip › Source data_revisions_v3/Supp. Fig. 1/Fig.S1A_BioA/2100 expert_High Sensitivity DNA Assay_DEDAE00921_2021-03-22_18-16-00.pdf]

Assay Class: High Sensitivity DNA Assay
Data Path: C:\...gh Sensitivity DNA Assay_DEDAE00921_2021-03-22_18-16-00.xad

Created: 3/22/2021 6:15:59 PM
Modified: 3/22/2021 6:57:10 PM

Electrophoresis File Run Summary

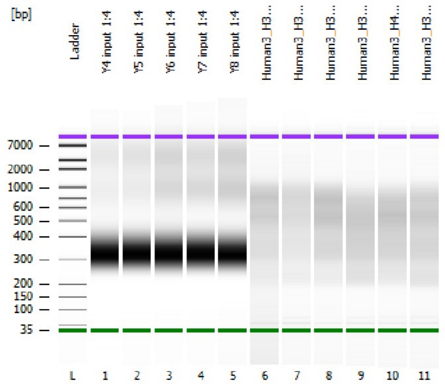

Instrument Information:

Instrument Name: DEDAE00921
Firmware: C.01.069
Serial#: DEDAE00921
Type: G2939B

Assay Information:

Assay Origin Path: C:\Program Files (x86)\Agilent\2100 bioanalyzer\2100 expert\assays\dsDNA\High Sensitivity DNA.xsy

Assay Class: High Sensitivity DNA Assay

Version: 1.03

Assay Comments: Copyright © 2003-2010 Agilent Technologies

Chip Information:

Chip Lot #:

Reagent Kit Lot #:

Chip Comments:

Y4 input 1:4

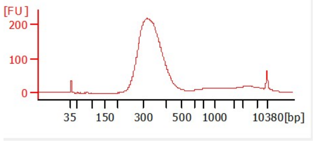

Y5 input 1:4

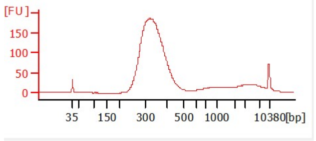

Y6 input 1:4

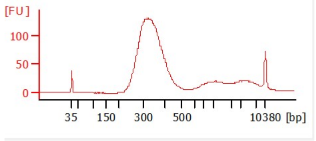

Y7 input 1:4

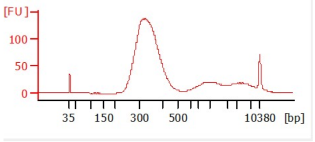

Y8 input 1:4

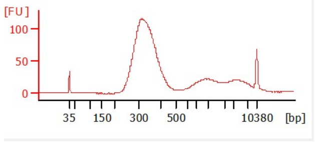

Human3_H3K9me3 1:11

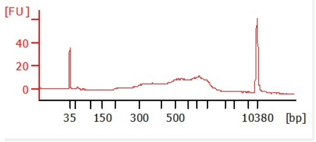

Human3_H3K4me3 1:6

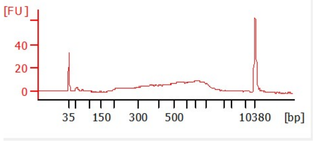

Human3_H3K4me1 1:16

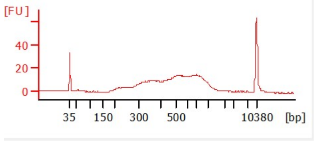

Human3_H3K36me3 1:10

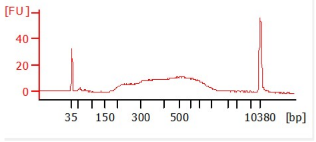

Human3_H4K20me2 1:14

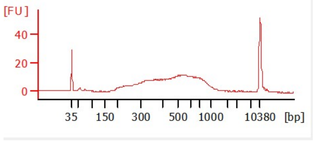

Human3_H3 1:13

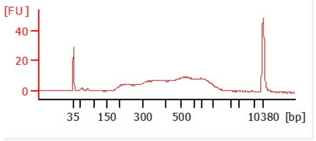

 © Copyright 2003 - 2018 Agilent, Inc.

Assay Class: High Sensitivity DNA Assay
Data Path: C:\...gh Sensitivity DNA Assay_DEDAE00921_2021-03-22_18-16-00.xad

Created: 3/22/2021 6:15:59 PM
Modified: 3/22/2021 6:57:10 PM

Electrophoresis File Run Summary (Chip Summary)

Sample Name	Sample Comment	Rest. Digest	Status	Observation	Result Label	Result Color
Y4 input 1:4		☐	✓			
Y5 input 1:4		☐	✓			
Y6 input 1:4		☐	✓			
Y7 input 1:4		☐	✓			
Y8 input 1:4		☐	✓			
Human3_H3K9me3 1:11		☐	✓			
Human3_H3K4me3 1:6		☐	✓			
Human3_H3K4me1 1:16		☐	✓			
Human3_H3K36me3 1:10		☐	✓			
Human3_H4K20me2 1:14		☐	✓			
Human3_H3 1:13		☐	✓			
Ladder		☐	✓			

Chip Lot # **Reagent Kit Lot #**

Chip Comments :

© Copyright 2003 - 2018 Agilent, Inc.

Assay Class: High Sensitivity DNA Assay
Data Path: C:\...gh Sensitivity DNA Assay_DEDAE00921_2021-03-22_18-16-00.xad
Created: 3/22/2021 6:15:59 PM
Modified: 3/22/2021 6:57:10 PM

Electrophoresis Assay Details

General Analysis Settings

Number of Available Sample and Ladder Wells (Max.) : 12
Minimum Visible Range [s] : 32
Maximum Visible Range [s] : 138
Start Analysis Time Range [s] : 33
End Analysis Time Range [s] : 137.5
Ladder Concentration [pg/µl] : 1950
Uses Standard Area for Ladder Fragments
Lower Marker Concentration [pg/µl] : 125
Upper Marker Concentration [pg/µl] : 75
Used Upper Marker for Quantitation
Standard Curve Fit is Point to Point
Show Data Aligned to Lower and Upper Marker

Integrator Settings

Integration Start Time [s] : 33.05
Integration End Time [s] : 137
Slope Threshold : 0.8
Height Threshold [FU] : 5
Area Threshold : 0.1
Width Threshold [s] : 0.6
Baseline Plateau [s] : 0.5

Filter Settings

Filter Width [s] : 0.5
Polynomial Order : 4

Ladder

Ladder Peak	Size	Area
1	35	160
2	50	210
3	100	208
4	150	221
5	200	242
6	300	270
7	400	305
8	500	306
9	600	336
10	700	321
11	1000	366
12	2000	413
13	3000	411
14	7000	400
15	10380	214

 © Copyright 2003 - 2018 Agilent, Inc.

Assay Class: High Sensitivity DNA Assay
Data Path: C:\...gh Sensitivity DNA Assay_DEDAE00921_2021-03-22_18-16-00.xad

Created: 3/22/2021 6:15:59 PM
Modified: 3/22/2021 6:57:10 PM

Electropherogram Summary

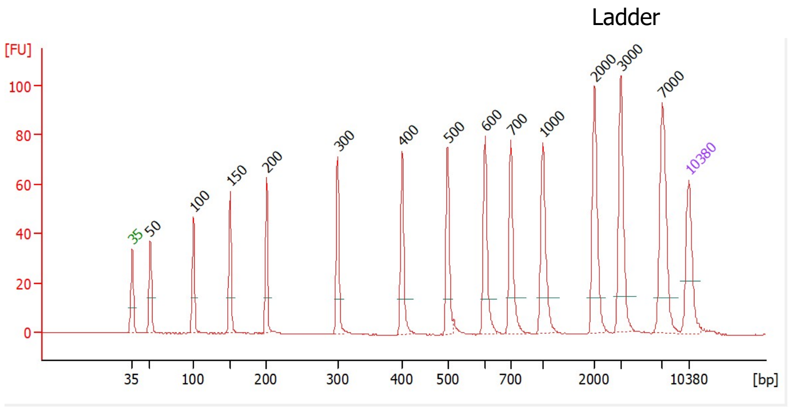

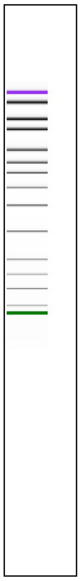

Overall Results for Ladder

Noise: 0.1

Peak table for Ladder

Peak		Size [bp]	Conc. [pg/µl]	Molarity [pmol/l]	Observations
1	◀	35	125.00	5,411.3	Lower Marker
2	∟	50	150.00	4,545.5	Ladder Peak
3	∟	100	150.00	2,272.7	Ladder Peak
4	∟	150	150.00	1,515.2	Ladder Peak
5	∟	200	150.00	1,136.4	Ladder Peak
6	∟	300	150.00	757.6	Ladder Peak
7	∟	400	150.00	568.2	Ladder Peak
8	∟	500	150.00	454.5	Ladder Peak
9	∟	600	150.00	378.8	Ladder Peak
10	∟	700	150.00	324.7	Ladder Peak
11	∟	1,000	150.00	227.3	Ladder Peak
12	∟	2,000	150.00	113.6	Ladder Peak
13	∟	3,000	150.00	75.8	Ladder Peak
14	∟	7,000	150.00	32.5	Ladder Peak
15	▶	10,380	75.00	10.9	Upper Marker

© Copyright 2003 - 2018 Agilent, Inc.

Assay Class:	High Sensitivity DNA Assay	Created:	3/22/2021 6:15:59 PM
Data Path:	C:\...gh Sensitivity DNA Assay_DEDAE00921_2021-03-22_18-16-00.xad	Modified:	3/22/2021 6:57:10 PM

Electropherogram Summary Continued ...

Y4 input 1:4

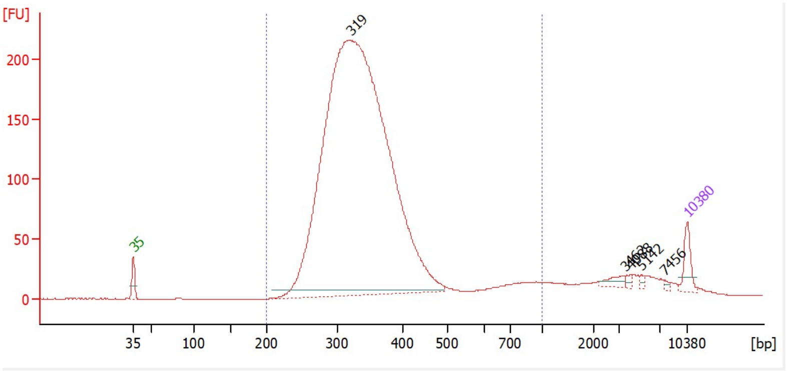

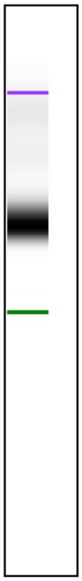

Overall Results for sample 1 : Y4 input 1:4

Number of peaks found:	5	Corr. Area 1:	3,213.0
Noise:	0.1		

Peak table for sample 1 : Y4 input 1:4

Peak		Size [bp]	Conc. [pg/µl]	Molarity [pmol/l]	Observations
1	◀	35	125.00	5,411.3	Lower Marker
2		319	6,948.66	33,002.4	
3		3,462	35.43	15.5	
4		4,088	12.93	4.8	
5		5,142	9.21	2.7	
6		7,456	9.19	1.9	
7	▶	10,380	75.00	10.9	Upper Marker

Region table for sample 1 : Y4 input 1:4

From [bp]	To [bp]	Corr. Area	% of Total	Average Size [bp]	Size distribution in CV [%]	Conc. [pg/µl]	Molarity [pmol/l]	Color
200	1,000	3,213.0	93	351	27.5	7,087.10	32,403.0	

 © Copyright 2003 - 2018 Agilent, Inc.

Assay Class: High Sensitivity DNA Assay
Data Path: C:\...gh Sensitivity DNA Assay_DEDAE00921_2021-03-22_18-16-00.xad
Created: 3/22/2021 6:15:59 PM
Modified: 3/22/2021 6:57:10 PM

Electropherogram Summary Continued ...

Y5 input 1:4

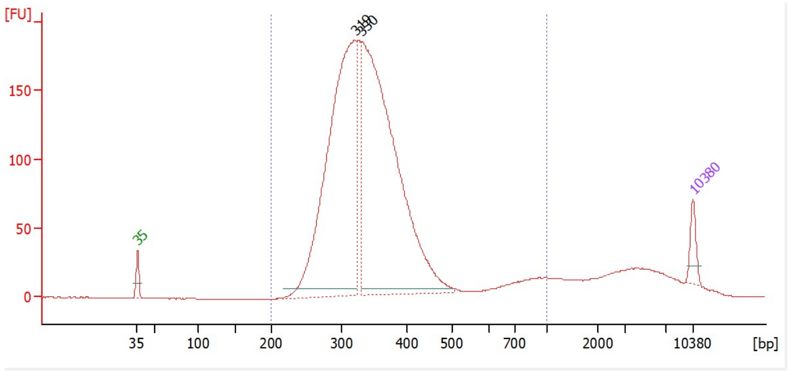

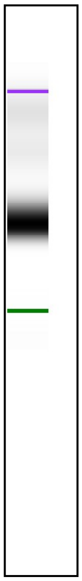

Overall Results for sample 2 : Y5 input 1:4

Number of peaks found:	2	Corr. Area 1:	2,780.7
Noise:	0.2		

Peak table for sample 2 : Y5 input 1:4

Peak		Size [bp]	Conc. [pg/µl]	Molarity [pmol/l]	Observations
1	◀	35	125.00	5,411.3	Lower Marker
2		319	2,900.71	13,789.7	
3		330	3,389.63	15,572.6	
4	▶	10,380	75.00	10.9	Upper Marker

Region table for sample 2 : Y5 input 1:4

From [bp]	To [bp]	Corr. Area	% of Total	Average Size [bp]	Size distribution in CV [%]	Conc. [pg/µl]	Molarity [pmol/l]	Co lor
200	1,000	2,780.7	91	356	29.2	6,876.12	31,184.7	■

 © Copyright 2003 - 2018 Agilent, Inc.

Assay Class:	High Sensitivity DNA Assay	Created:	3/22/2021 6:15:59 PM
Data Path:	C:\...gh Sensitivity DNA Assay_DEDAE00921_2021-03-22_18-16-00.xad	Modified:	3/22/2021 6:57:10 PM

Electropherogram Summary Continued ...

Y6 input 1:4

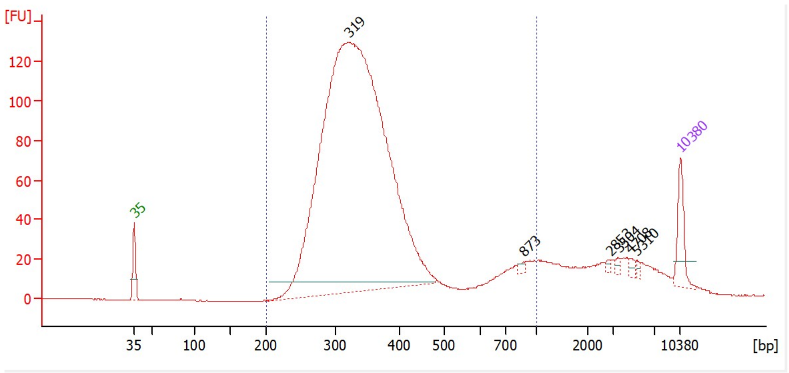

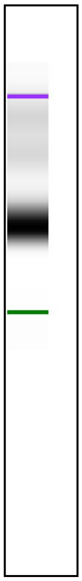

Overall Results for sample 3 : Y6 input 1:4

Number of peaks found:	6	Corr. Area 1:	2,060.8
Noise:	0.2		

Peak table for sample 3 : Y6 input 1:4

Peak		Size [bp]	Conc. [pg/µl]	Molarity [pmol/l]	Observations
1	◀	35	125.00	5,411.3	Lower Marker
2		319	3,927.41	18,641.1	
3		873	9.84	17.1	
4		2,853	6.43	3.4	
5		3,504	7.33	3.2	
6		4,708	9.56	3.1	
7		5,310	7.00	2.0	
8	▶	10,380	75.00	10.9	Upper Marker

Region table for sample 3 : Y6 input 1:4

From [bp]	To [bp]	Corr. Area	% of Total	Average Size [bp]	Size distribution in CV [%]	Conc. [pg/µl]	Molarity [pmol/l]	Color
200	1,000	2,060.8	88	369	34.9	4,262.49	19,154.8	■

© Copyright 2003 - 2018 Agilent, Inc.

Assay Class:	High Sensitivity DNA Assay	Created:	3/22/2021 6:15:59 PM
Data Path:	C:\...gh Sensitivity DNA Assay_DEDAE00921_2021-03-22_18-16-00.xad	Modified:	3/22/2021 6:57:10 PM

Electropherogram Summary Continued ...

Y7 input 1:4

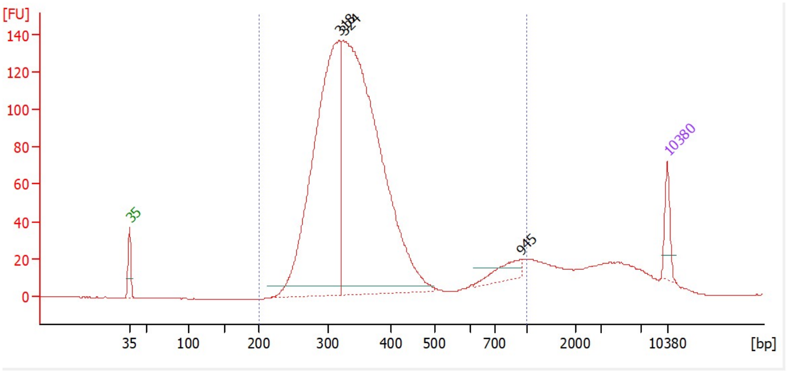

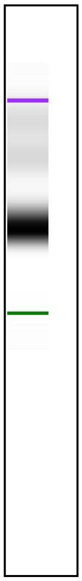

Overall Results for sample 4 : **<u>Y7 input 1:4</u>**

Number of peaks found:	3	Corr. Area 1:	2,094.9
Noise:	0.2		

Peak table for sample 4 : **Y7 input 1:4**

Peak		Size [bp]	Conc. [pg/µl]	Molarity [pmol/l]	Observations
1	◀	35	125.00	5,411.3	Lower Marker
2		318	2,075.90	9,897.1	
3		324	2,919.60	13,638.9	
4		945	90.47	145.0	
5	▶	10,380	75.00	10.9	Upper Marker

Region table for sample 4 : **Y7 input 1:4**

From [bp]	To [bp]	Corr. Area	% of Total	Average Size [bp]	Size distribution in CV [%]	Conc. [pg/µl]	Molarity [pmol/l]	Co lor
200	1,000	2,094.9	88	370	35.0	5,164.89	23,123.7	■

 © Copyright 2003 - 2018 Agilent, Inc.

Assay Class: High Sensitivity DNA Assay
Data Path: C:\...gh Sensitivity DNA Assay_DEDAE00921_2021-03-22_18-16-00.xad
Created: 3/22/2021 6:15:59 PM
Modified: 3/22/2021 6:57:10 PM

Electropherogram Summary Continued ...

Y8 input 1:4

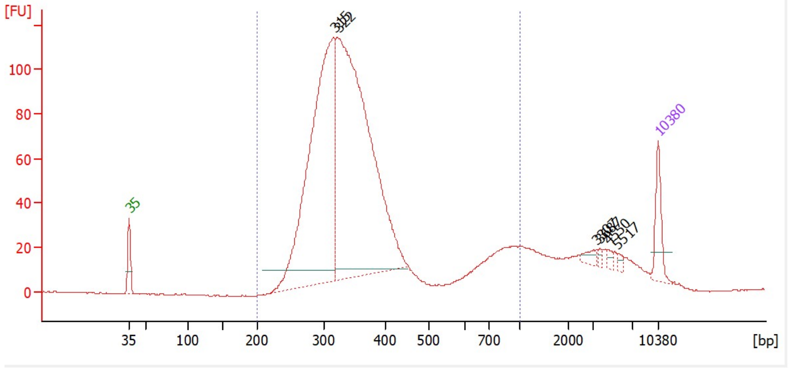

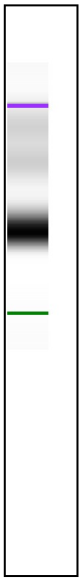

Overall Results for sample 5 : **Y8 input 1:4**

Number of peaks found: 6
Corr. Area 1: 1,710.8
Noise: 0.3

Peak table for sample 5 : **Y8 input 1:4**

Peak		Size [bp]	Conc. [pg/µl]	Molarity [pmol/l]	Observations
1	◀	35	125.00	5,411.3	Lower Marker
2		315	1,503.22	7,228.2	
3		322	1,935.43	9,114.1	
4		3,307	19.09	8.7	
5		3,687	7.13	2.9	
6		4,550	9.92	3.3	
7		5,517	10.62	2.9	
8	▶	10,380	75.00	10.9	Upper Marker

Region table for sample 5 : **Y8 input 1:4**

From [bp]	To [bp]	Corr. Area	% of Total	Average Size [bp]	Size distribution in CV [%]	Conc. [pg/µl]	Molarity [pmol/l]	Color
200	1,000	1,710.8	86	378	38.3	3,978.94	17,765.3	■

© Copyright 2003 - 2018 Agilent, Inc.

Assay Class: High Sensitivity DNA Assay
Data Path: C:\...gh Sensitivity DNA Assay_DEDAE00921_2021-03-22_18-16-00.xad

Created: 3/22/2021 6:15:59 PM
Modified: 3/22/2021 6:57:10 PM

Electropherogram Summary Continued ...

Human3_H3K9me3 1:11

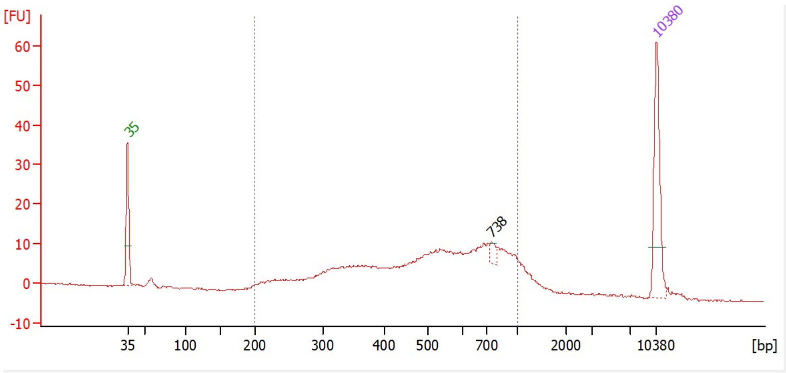

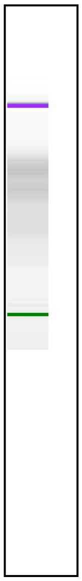

Overall Results for sample 6 : Human3_H3K9me3 1:11

Number of peaks found:	1	Corr. Area 1:	308.3
Noise:	0.1		

Peak table for sample 6 : Human3_H3K9me3 1:11

Peak		Size [bp]	Conc. [pg/µl]	Molarity [pmol/l]	Observations
1	◀	35	125.00	5,411.3	Lower Marker
2		738	10.43	21.4	
3	▶	10,380	75.00	10.9	Upper Marker

Region table for sample 6 : Human3_H3K9me3 1:11

From [bp]	To [bp]	Corr. Area	% of Total	Average Size [bp]	Size distribution in CV [%]	Conc. [pg/µl]	Molarity [pmol/l]	Color
200	1,000	308.3	90	539	35.5	628.90	2,164.8	■

© Copyright 2003 - 2018 Agilent, Inc.

Assay Class:	High Sensitivity DNA Assay	Created:	3/22/2021 6:15:59 PM
Data Path:	C:\...gh Sensitivity DNA Assay_DEDAE00921_2021-03-22_18-16-00.xad	Modified:	3/22/2021 6:57:10 PM

Electropherogram Summary Continued ...

Human3_H3K4me3 1:6

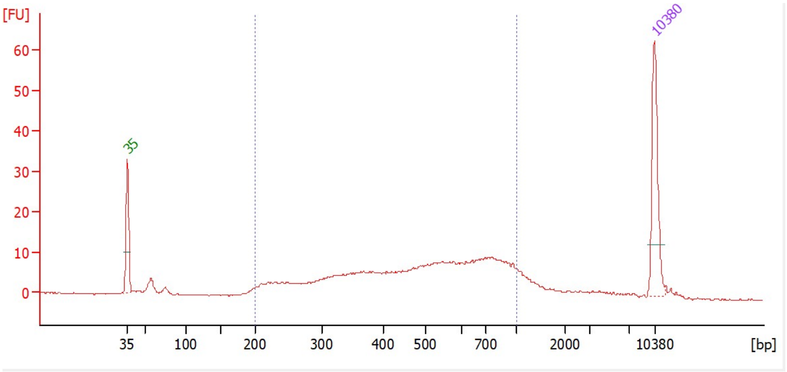

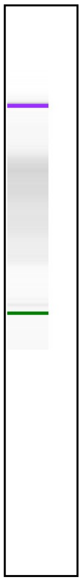

Overall Results for sample 7 : Human3_H3K4me3 1:6

Number of peaks found:	0	Corr. Area 1:	269.1
Noise:	0.1		

Peak table for sample 7 : Human3_H3K4me3 1:6

Peak		Size [bp]	Conc. [pg/µl]	Molarity [pmol/l]	Observations
1	◀	35	125.00	5,411.3	Lower Marker
2	▶	10,380	75.00	10.9	Upper Marker

Region table for sample 7 : Human3_H3K4me3 1:6

From [bp]	To [bp]	Corr. Area	% of Total	Average Size [bp]	Size distribution in CV [%]	Conc. [pg/µl]	Molarity [pmol/l]	Color
200	1,000	269.1	83	519	37.6	550.69	2,011.8	■

 © Copyright 2003 - 2018 Agilent, Inc.

Assay Class:	High Sensitivity DNA Assay	Created:	3/22/2021 6:15:59 PM
Data Path:	C:\...gh Sensitivity DNA Assay_DEDAE00921_2021-03-22_18-16-00.xad	Modified:	3/22/2021 6:57:10 PM

Electropherogram Summary Continued ...

Human3_H3K4me1 1:16

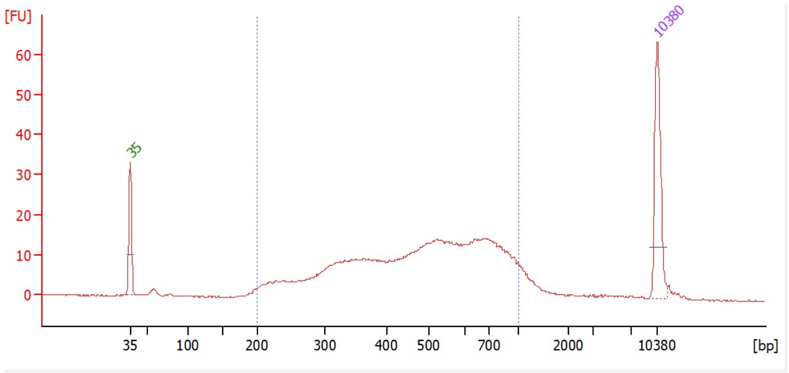

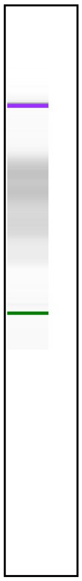

Overall Results for sample 8 : **Human3_H3K4me1 1:16**

Number of peaks found:	0	Corr. Area 1:	424.7
Noise:	0.1		

Peak table for sample 8 : **Human3_H3K4me1 1:16**

Peak		Size [bp]	Conc. [pg/µl]	Molarity [pmol/l]	Observations
1	◀	35	125.00	5,411.3	Lower Marker
2	▶	10,380	75.00	10.9	Upper Marker

Region table for sample 8 : **Human3_H3K4me1 1:16**

From [bp]	To [bp]	Corr. Area	% of Total	Average Size [bp]	Size distribution in CV [%]	Conc. [pg/µl]	Molarity [pmol/l]	Co lor
200	1,000	424.7	91	513	35.8	835.54	3,013.0	■

 © Copyright 2003 - 2018 Agilent, Inc.

Assay Class: High Sensitivity DNA Assay
Data Path: C:\...gh Sensitivity DNA Assay_DEDAE00921_2021-03-22_18-16-00.xad

Created: 3/22/2021 6:15:59 PM
Modified: 3/22/2021 6:57:10 PM

Electropherogram Summary Continued ...

Human3_H3K36me3 1:10

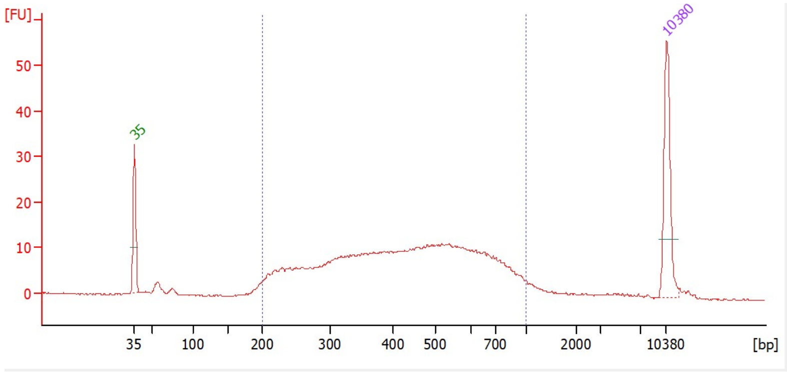

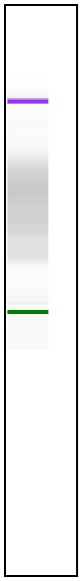

Overall Results for sample 9 : Human3 H3K36me3 1:10

Number of peaks found:	0	Corr. Area 1:	386.5
Noise:	0.1		

Peak table for sample 9 : Human3 H3K36me3 1:10

Peak		Size [bp]	Conc. [pg/µl]	Molarity [pmol/l]	Observations
1	◀	35	125.00	5,411.3	Lower Marker
2	▶	10,380	75.00	10.9	Upper Marker

Region table for sample 9 : Human3 H3K36me3 1:10

From [bp]	To [bp]	Corr. Area	% of Total	Average Size [bp]	Size distribution in CV [%]	Conc. [pg/µl]	Molarity [pmol/l]	Co lor
200	1,000	386.5	92	461	36.6	852.37	3,401.7	■

 © Copyright 2003 - 2018 Agilent, Inc.

Assay Class: High Sensitivity DNA Assay
Data Path: C:\...gh Sensitivity DNA Assay_DEDAE00921_2021-03-22_18-16-00.xad

Created: 3/22/2021 6:15:59 PM
Modified: 3/22/2021 6:57:10 PM

Electropherogram Summary Continued ...

Human3_H4K20me2 1:14

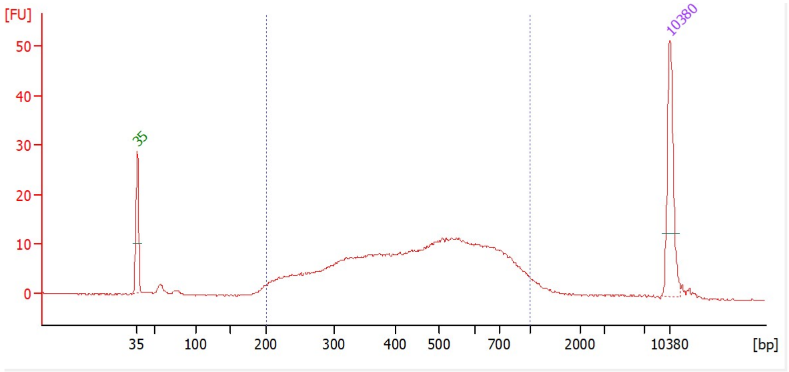

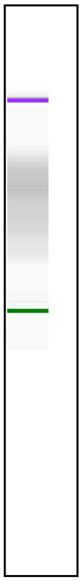

Overall Results for sample 10 : Human3_H4K20me2 1:14

Number of peaks found:	0	Corr. Area 1:	352.9
Noise:	0.1		

Peak table for sample 10 : Human3_H4K20me2 1:14

Peak		Size [bp]	Conc. [pg/µl]	Molarity [pmol/l]	Observations
1	◀	35	125.00	5,411.3	Lower Marker
2	▶	10,380	75.00	10.9	Upper Marker

Region table for sample 10 : Human3_H4K20me2 1:14

From [bp]	To [bp]	Corr. Area	% of Total	Average Size [bp]	Size distribution in CV [%]	Conc. [pg/µl]	Molarity [pmol/l]	Color
200	1,000	352.9	92	483	35.7	849.74	3,222.2	■

© Copyright 2003 - 2018 Agilent, Inc.

Assay Class: High Sensitivity DNA Assay
Data Path: C:\...gh Sensitivity DNA Assay_DEDAE00921_2021-03-22_18-16-00.xad

Created: 3/22/2021 6:15:59 PM
Modified: 3/22/2021 6:57:10 PM

Electropherogram Summary Continued ...

Human3_H3 1:13

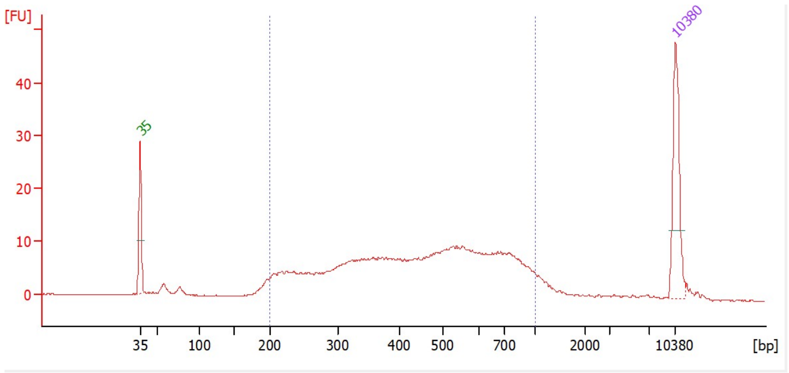

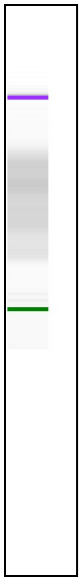

Overall Results for sample 11 : Human3_H3 1:13

Number of peaks found: 0 Corr. Area 1: 312.5

Noise: 0.1

Peak table for sample 11 : Human3_H3 1:13

Peak		Size [bp]	Conc. [pg/µl]	Molarity [pmol/l]	Observations
1	◀	35	125.00	5,411.3	Lower Marker
2	▶	10,380	75.00	10.9	Upper Marker

Region table for sample 11 : Human3_H3 1:13

From [bp]	To [bp]	Corr. Area	% of Total	Average Size [bp]	Size distribution in CV [%]	Conc. [pg/µl]	Molarity [pmol/l]	Co lor
200	1,000	312.5	89	481	38.2	819.91	3,208.2	■

 © Copyright 2003 - 2018 Agilent, Inc.

Assay Class: High Sensitivity DNA Assay
Data Path: C:\...gh Sensitivity DNA Assay_DEDAE00921_2021-03-22_18-16-00.xad

Created: 3/22/2021 6:15:59 PM
Modified: 3/22/2021 6:57:10 PM

Gel Image

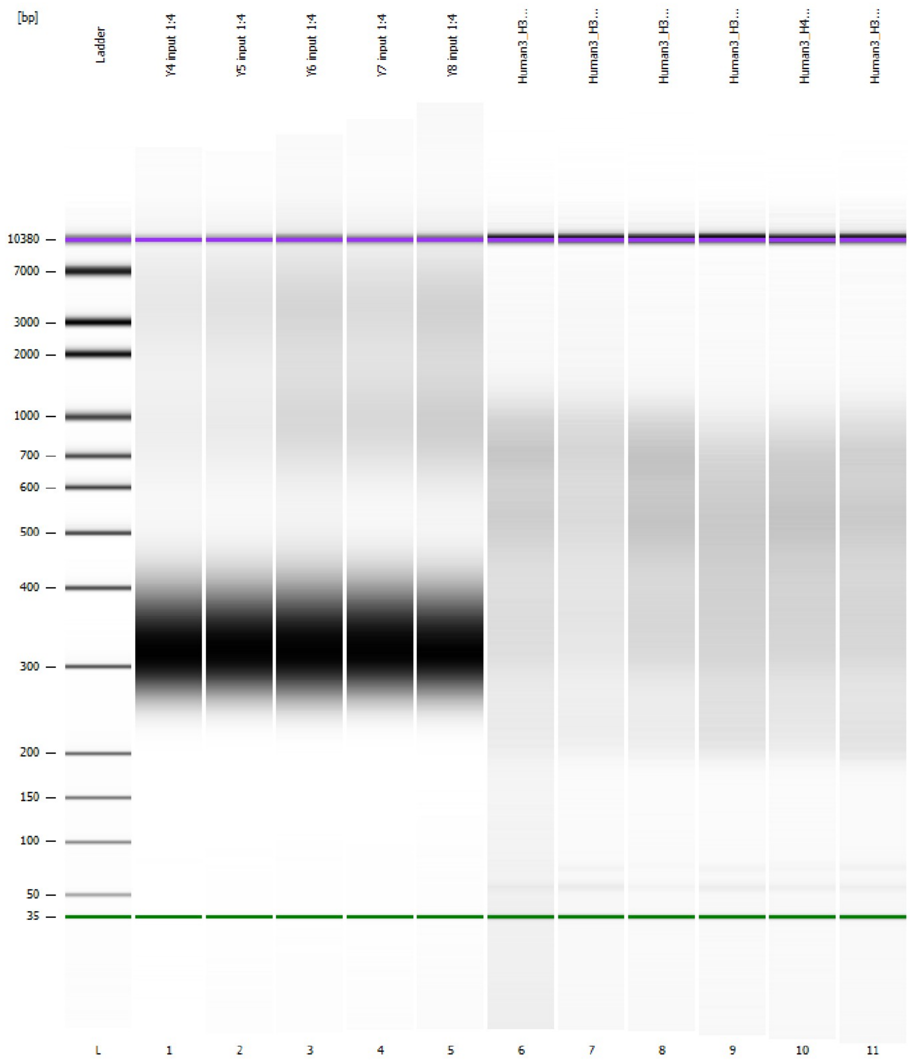

© Copyright 2003 - 2018 Agilent, Inc.

Assay Class: High Sensitivity DNA Assay
Data Path: C:\...gh Sensitivity DNA Assay_DEDAE00921_2021-03-22_18-16-00.xad
Created: 3/22/2021 6:15:59 PM
Modified: 3/22/2021 6:57:10 PM

Run Logbook

Description	Number	Source	Category	Sub Category	Time	Time Zone	User	Host
Run ended on port 3 (Number of wells acquired: 12)		Instrument	Run		3/22/2021 6:56:22 PM	(GMT --04:00) Eastern Standard Time	senlab	NIALGG-02162892
Run started on port 3 (File: C:\Program Files (x86)\Agilent\2100 bioanalyzer\2100 expert\Data\2021-03-22\2100 expert_High Sensitivity DNA Assay_DEDAE00921_2021-03-22_18-16-00.xad)		Instrument	Run		3/22/2021 6:15:59 PM	(GMT --04:00) Eastern Standard Time	senlab	NIALGG-02162892
Product Number : G2939B		Instrument	Run		3/22/2021 6:15:59 PM	(GMT --04:00) Eastern Standard Time	senlab	NIALGG-02162892
Name :		Instrument	Run		3/22/2021 6:15:59 PM	(GMT --04:00) Eastern Standard Time	senlab	NIALGG-02162892
Vendor : Agilent Technologies		Instrument	Run		3/22/2021 6:15:59 PM	(GMT --04:00) Eastern Standard Time	senlab	NIALGG-02162892
Serial# : DEDAE00921		Instrument	Run		3/22/2021 6:15:59 PM	(GMT --04:00) Eastern Standard Time	senlab	NIALGG-02162892
Firmware : C.01.069		Instrument	Run		3/22/2021 6:15:59 PM	(GMT --04:00) Eastern Standard Time	senlab	NIALGG-02162892
Cartridge : Electrode		Instrument	Run		3/22/2021 6:15:59 PM	(GMT --04:00) Eastern Standard Time	senlab	NIALGG-02162892

© Copyright 2003 - 2018 Agilent, Inc.
