# Supplementary material for: Gene body DNA hydroxymethylation restricts the magnitude of transcriptional changes during aging
Source: Nat Commun. 2024 Jul 28;15:6357. doi: 10.1038/s41467-024-50725-y (PMC11284234; doi:10.1038/s41467-024-50725-y)
Supplement: Supplementary file 17 — Source Data [file 41467_2024_50725_MOESM17_ESM.zip › Source data_revisions_v3/Supp. Fig. 2/Fig.S2A_BioA/2100 expert_High Sensitivity DNA Assay_DEDAE00921_2022-09-01_12-23-18.pdf]

Assay Class: High Sensitivity DNA Assay
Data Path: C:\...gh Sensitivity DNA Assay_DEDAE00921_2022-09-01_12-23-18.xad

Created: 9/1/2022 12:23:17 PM
Modified: 9/1/2022 1:04:38 PM

Electrophoresis File Run Summary

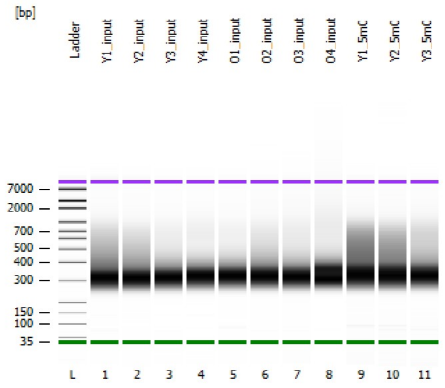

Instrument Information:

Instrument Name:	DEDAE00921	Firmware:	C.01.069
Serial#:	DEDAE00921	Type:	G2939B

Assay Information:

Assay Origin Path:	C:\Program Files (x86)\Agilent\2100 bioanalyzer\2100 expert\assays\dsDNA\High Sensitivity DNA.xsy
Assay Class:	High Sensitivity DNA Assay
Version:	1.03
Assay Comments:	Copyright © 2003-2010 Agilent Technologies

Chip Information:

Chip Lot #:

Reagent Kit Lot #:

Chip Comments:

Y1_input

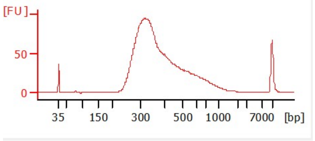

Y2_input

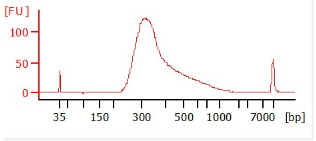

Y3_input

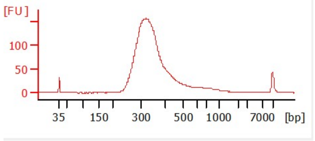

Y4_input

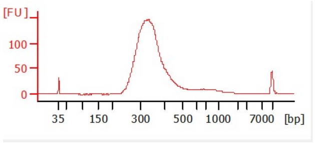

O1_input

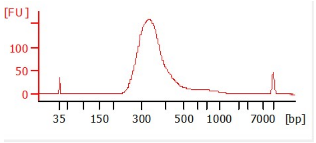

O2_input

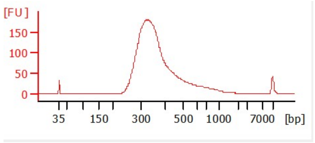

O3_input

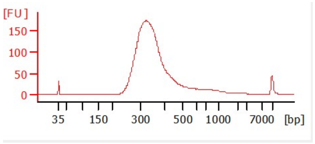

O4_input

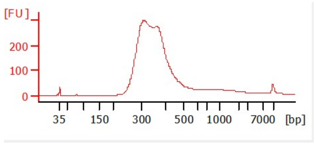

Y1_5mC

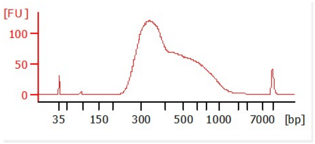

Y2_5mC

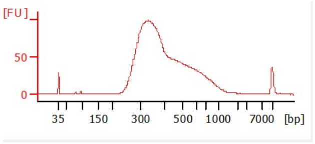

Y3_5mC

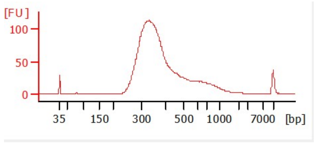

© Copyright 2003 - 2018 Agilent, Inc.

Assay Class: High Sensitivity DNA Assay
Data Path: C:\...gh Sensitivity DNA Assay_DEDAE00921_2022-09-01_12-23-18.xad
Created: 9/1/2022 12:23:17 PM
Modified: 9/1/2022 1:04:38 PM

Electrophoresis File Run Summary (Chip Summary)

Sample Name	Sample Comment	Rest. Digest	Status	Observation	Result Label	Result Color
Y1_input		☐	✓			
Y2_input		☐	✓			
Y3_input		☐	✓			
Y4_input		☐	✓			
O1_input		☐	✓			
O2_input		☐	✓			
O3_input		☐	✓			
O4_input		☐	✓			
Y1_5mC		☐	✓			
Y2_5mC		☐	✓			
Y3_5mC		☐	✓			
Ladder		☐	✓			

Chip Lot # **Reagent Kit Lot #**

Chip Comments :

 © Copyright 2003 - 2018 Agilent, Inc.

Assay Class: High Sensitivity DNA Assay
Data Path: C:\...gh Sensitivity DNA Assay_DEDAE00921_2022-09-01_12-23-18.xad
Created: 9/1/2022 12:23:17 PM
Modified: 9/1/2022 1:04:38 PM

Electropherogram Summary

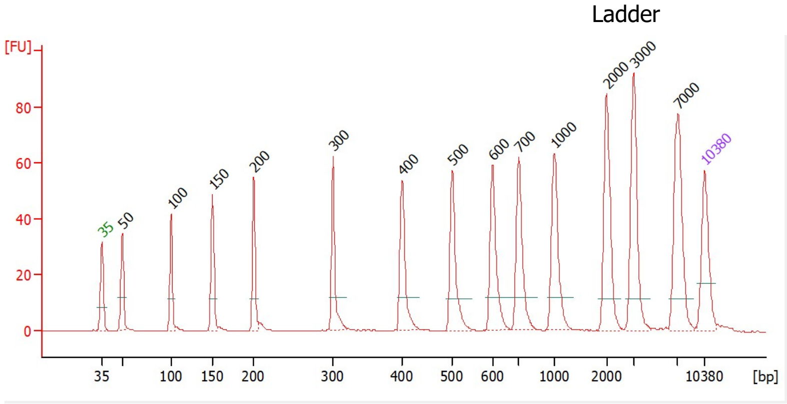

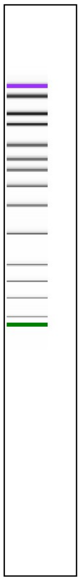

Overall Results for Ladder

Noise: 0.1

Peak table for Ladder

Peak		Size [bp]	Conc. [pg/µl]	Molarity [pmol/l]	Observations
1	◀	35	125.00	5,411.3	Lower Marker
2	∟	50	150.00	4,545.5	Ladder Peak
3	∟	100	150.00	2,272.7	Ladder Peak
4	∟	150	150.00	1,515.2	Ladder Peak
5	∟	200	150.00	1,136.4	Ladder Peak
6	∟	300	150.00	757.6	Ladder Peak
7	∟	400	150.00	568.2	Ladder Peak
8	∟	500	150.00	454.5	Ladder Peak
9	∟	600	150.00	378.8	Ladder Peak
10	∟	700	150.00	324.7	Ladder Peak
11	∟	1,000	150.00	227.3	Ladder Peak
12	∟	2,000	150.00	113.6	Ladder Peak
13	∟	3,000	150.00	75.8	Ladder Peak
14	∟	7,000	150.00	32.5	Ladder Peak
15	▶	10,380	75.00	10.9	Upper Marker

© Copyright 2003 - 2018 Agilent, Inc.

Assay Class: High Sensitivity DNA Assay
Data Path: C:\...gh Sensitivity DNA Assay_DEDAE00921_2022-09-01_12-23-18.xad
Created: 9/1/2022 12:23:17 PM
Modified: 9/1/2022 1:04:38 PM

Electropherogram Summary Continued ...

Y1_input

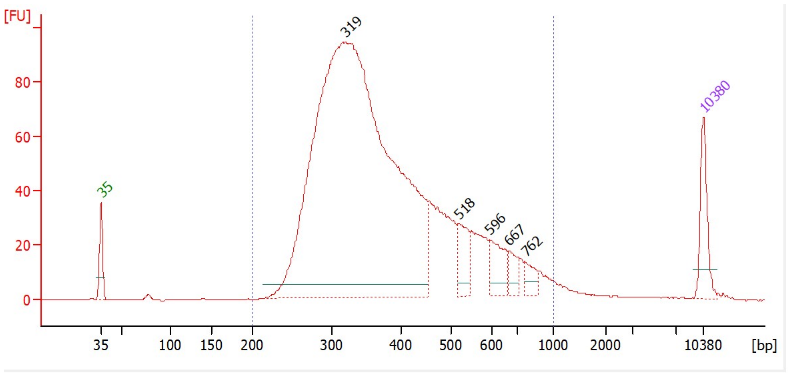

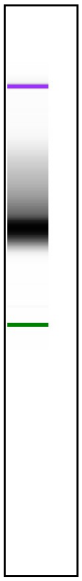

Overall Results for sample 1 : Y1_input

Number of peaks found:	5	Corr. Area 1:	1,750.1
Noise:	0.1		

Peak table for sample 1 : Y1_input

Peak		Size [bp]	Conc. [pg/µl]	Molarity [pmol/l]	Observations
1	◀	35	125.00	5,411.3	Lower Marker
2		319	2,911.83	13,817.6	
3		518	82.00	240.0	
4		596	79.16	201.4	
5		667	37.31	84.8	
6		762	31.84	63.3	
7	▶	10,380	75.00	10.9	Upper Marker

Region table for sample 1 : Y1_input

From [bp]	To [bp]	Corr. Area	% of Total	Average Size [bp]	Size distribution in CV [%]	Conc. [pg/µl]	Molarity [pmol/l]	Co lor
200	1,000	1,750.1	98	396	32.9	3,539.42	15,136.0	

© Copyright 2003 - 2018 Agilent, Inc.

Assay Class: High Sensitivity DNA Assay
Data Path: C:\...gh Sensitivity DNA Assay_DEDAE00921_2022-09-01_12-23-18.xad

Created: 9/1/2022 12:23:17 PM
Modified: 9/1/2022 1:04:38 PM

Electropherogram Summary Continued ...

Y2_input

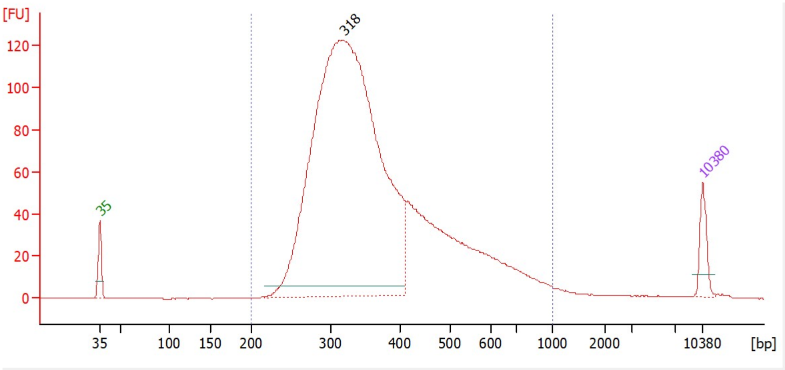

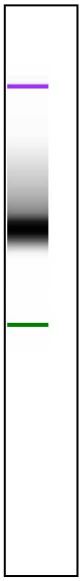

Overall Results for sample 2 : Y2_input

Number of peaks found:	1	Corr. Area 1:	2,039.7
Noise:	0.1		

Peak table for sample 2 : Y2_input

Peak		Size [bp]	Conc. [pg/µl]	Molarity [pmol/l]	Observations
1	◀	35	125.00	5,411.3	Lower Marker
2		318	4,174.17	19,893.2	
3	▶	10,380	75.00	10.9	Upper Marker

Region table for sample 2 : Y2_input

From [bp]	To [bp]	Corr. Area	% of Total	Average Size [bp]	Size distribution in CV [%]	Conc. [pg/µl]	Molarity [pmol/l]	Color
200	1,000	2,039.7	98	381	31.2	5,316.32	23,283.1	■

 © Copyright 2003 - 2018 Agilent, Inc.

Assay Class: High Sensitivity DNA Assay
Data Path: C:\...gh Sensitivity DNA Assay_DEDAE00921_2022-09-01_12-23-18.xad
Created: 9/1/2022 12:23:17 PM
Modified: 9/1/2022 1:04:38 PM

Electropherogram Summary Continued ...

Y3_input

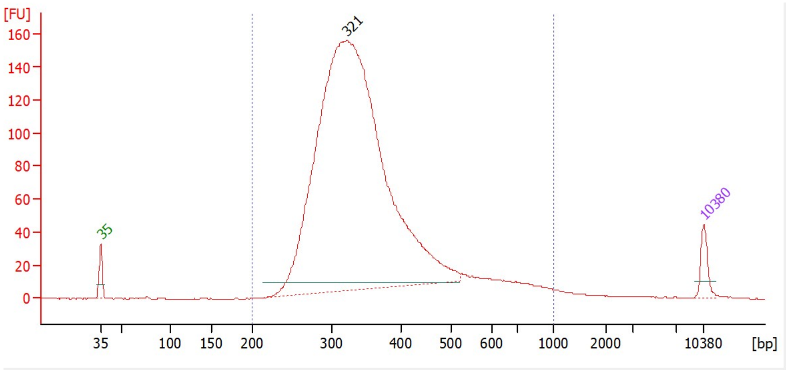

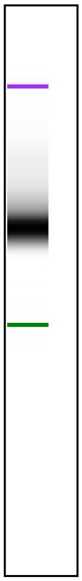

Overall Results for sample 3 : Y3_input

Number of peaks found: 1
Corr. Area 1: 2,201.4
Noise: 0.1

Peak table for sample 3 : Y3_input

Peak		Size [bp]	Conc. [pg/µl]	Molarity [pmol/l]	Observations
1	◀	35	125.00	5,411.3	Lower Marker
2		321	6,219.84	29,395.3	
3	▶	10,380	75.00	10.9	Upper Marker

Region table for sample 3 : Y3_input

From [bp]	To [bp]	Corr. Area	% of Total	Average Size [bp]	Size distribution in CV [%]	Conc. [pg/µl]	Molarity [pmol/l]	Color
200	1,000	2,201.4	98	362	28.7	6,955.45	31,301.6	

© Copyright 2003 - 2018 Agilent, Inc.

Assay Class:	High Sensitivity DNA Assay	Created:	9/1/2022 12:23:17 PM
Data Path:	C:\...gh Sensitivity DNA Assay_DEDAE00921_2022-09-01_12-23-18.xad	Modified:	9/1/2022 1:04:38 PM

Electropherogram Summary Continued ...

Y4_input

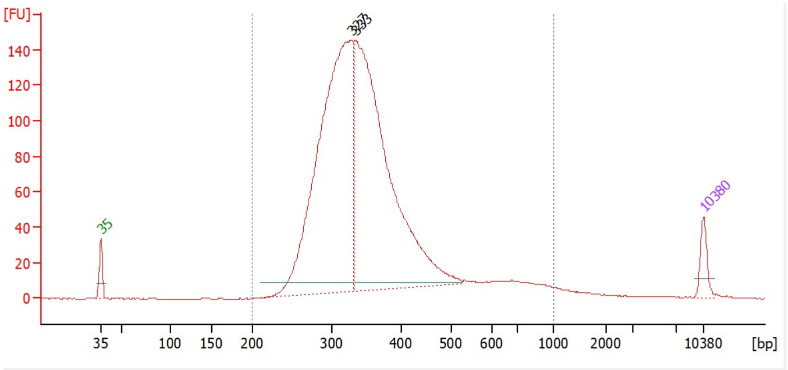

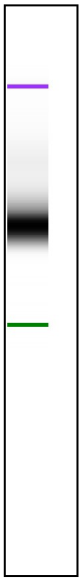

Overall Results for sample 4 : **Y4_input**

Number of peaks found:	2	Corr. Area 1:	2,026.9
Noise:	0.1		

Peak table for sample 4 : **Y4_input**

Peak		Size [bp]	Conc. [pg/µl]	Molarity [pmol/l]	Observations
1	◀	35	125.00	5,411.3	Lower Marker
2		327	2,851.01	13,203.8	
3		333	2,778.93	12,640.0	
4	▶	10,380	75.00	10.9	Upper Marker

Region table for sample 4 : **Y4_input**

From [bp]	To [bp]	Corr. Area	% of Total	Average Size [bp]	Size distribution in CV [%]	Conc. [pg/µl]	Molarity [pmol/l]	Co lor
200	1,000	2,026.9	98	363	28.7	6,366.25	28,483.8	■

 © Copyright 2003 - 2018 Agilent, Inc.

Assay Class: High Sensitivity DNA Assay
Data Path: C:\...gh Sensitivity DNA Assay_DEDAE00921_2022-09-01_12-23-18.xad
Created: 9/1/2022 12:23:17 PM
Modified: 9/1/2022 1:04:38 PM

Electropherogram Summary Continued ...

O1_input

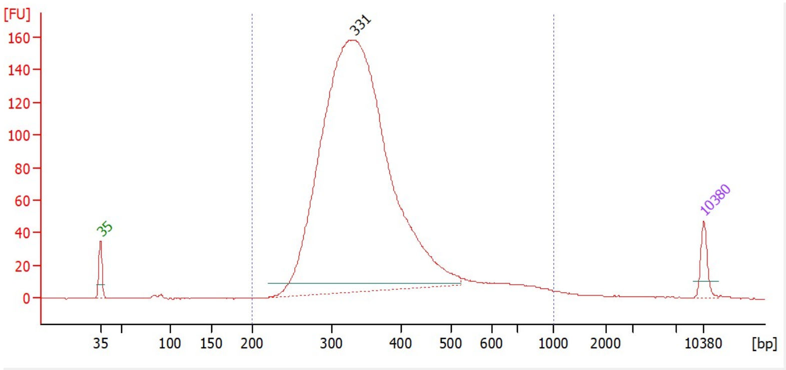

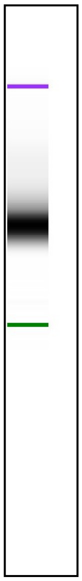

Overall Results for sample 5 : O1_input

Number of peaks found: 1 Corr. Area 1: 2,164.6

Noise: 0.1

Peak table for sample 5 : O1_input

Peak		Size [bp]	Conc. [pg/µl]	Molarity [pmol/l]	Observations
1	◀	35	125.00	5,411.3	Lower Marker
2		331	6,110.18	27,984.7	
3	▶	10,380	75.00	10.9	Upper Marker

Region table for sample 5 : O1_input

From [bp]	To [bp]	Corr. Area	% of Total	Average Size [bp]	Size distribution in CV [%]	Conc. [pg/µl]	Molarity [pmol/l]	Co lor
200	1,000	2,164.6	98	362	26.9	6,729.33	29,961.9	

© Copyright 2003 - 2018 Agilent, Inc.

Assay Class: High Sensitivity DNA Assay
Data Path: C:\...gh Sensitivity DNA Assay_DEDAE00921_2022-09-01_12-23-18.xad

Created: 9/1/2022 12:23:17 PM
Modified: 9/1/2022 1:04:38 PM

Electropherogram Summary Continued ...

O2_input

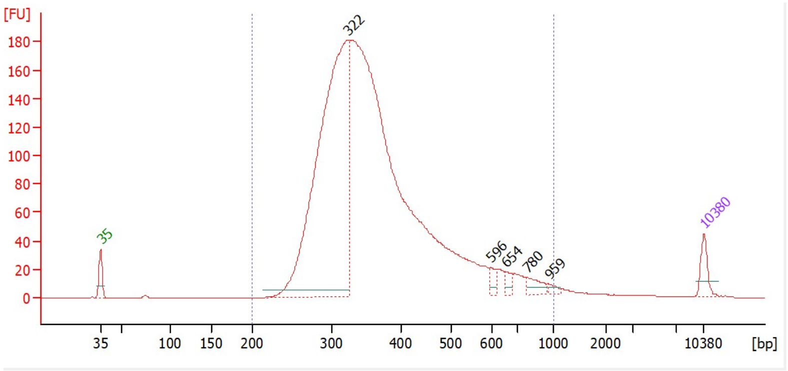

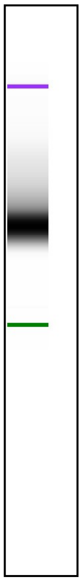

Overall Results for sample 6 : O2_input

Number of peaks found: 5

Corr. Area 1: 2,764.1

Noise: 0.1

Peak table for sample 6 : O2_input

Peak		Size [bp]	Conc. [pg/µl]	Molarity [pmol/l]	Observations
1	◄	35	125.00	5,411.3	Lower Marker
2		322	3,461.16	16,298.7	
3		596	51.31	130.4	
4		654	43.10	99.8	
5		780	71.53	139.0	
6		959	23.22	36.7	
7	►	10,380	75.00	10.9	Upper Marker

Region table for sample 6 : O2_input

From [bp]	To [bp]	Corr. Area	% of Total	Average Size [bp]	Size distribution in CV [%]	Conc. [pg/µl]	Molarity [pmol/l]	Color
200	1,000	2,764.1	98	378	29.7	9,060.20	39,375.2	■

 © Copyright 2003 - 2018 Agilent, Inc.

Assay Class: High Sensitivity DNA Assay
Data Path: C:\...gh Sensitivity DNA Assay_DEDAE00921_2022-09-01_12-23-18.xad

Created: 9/1/2022 12:23:17 PM
Modified: 9/1/2022 1:04:38 PM

Electropherogram Summary Continued ...

O3_input

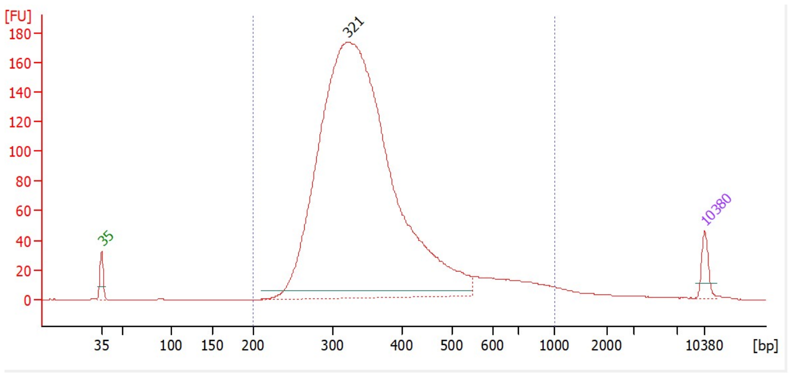

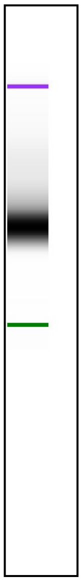

Overall Results for sample 7 : O3_input

Number of peaks found: 1
Corr. Area 1: 2,499.4
Noise: 0.1

Peak table for sample 7 : O3_input

Peak		Size [bp]	Conc. [pg/µl]	Molarity [pmol/l]	Observations
1	◀	35	125.00	5,411.3	Lower Marker
2		321	7,453.98	35,175.0	
3	▶	10,380	75.00	10.9	Upper Marker

Region table for sample 7 : O3_input

From [bp]	To [bp]	Corr. Area	% of Total	Average Size [bp]	Size distribution in CV [%]	Conc. [pg/µl]	Molarity [pmol/l]	Co lor
200	1,000	2,499.4	97	368	29.8	7,719.21	34,306.1	

 © Copyright 2003 - 2018 Agilent, Inc.

Assay Class: High Sensitivity DNA Assay
Data Path: C:\...gh Sensitivity DNA Assay_DEDAE00921_2022-09-01_12-23-18.xad

Created: 9/1/2022 12:23:17 PM
Modified: 9/1/2022 1:04:38 PM

Electropherogram Summary Continued ...

O4_input

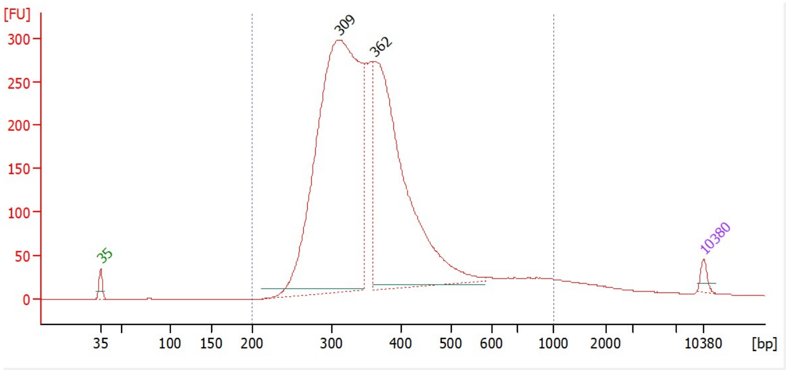

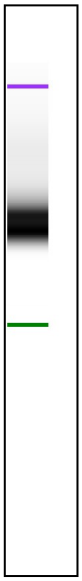

Overall Results for sample 8 : O4_input

Number of peaks found:	2	Corr. Area 1:	4,708.7
Noise:	0.1		

Peak table for sample 8 : O4_input

Peak		Size [bp]	Conc. [pg/µl]	Molarity [pmol/l]	Observations
1	◀	35	125.00	5,411.3	Lower Marker
2		309	9,677.97	47,458.8	
3		362	5,539.81	23,211.8	
4	▶	10,380	75.00	10.9	Upper Marker

Region table for sample 8 : O4_input

From [bp]	To [bp]	Corr. Area	% of Total	Average Size [bp]	Size distribution in CV [%]	Conc. [pg/µl]	Molarity [pmol/l]	Color
200	1,000	4,708.7	95	372	29.8	18,194.77	79,765.9	

 © Copyright 2003 - 2018 Agilent, Inc.

Assay Class:	High Sensitivity DNA Assay	Created:	9/1/2022 12:23:17 PM
Data Path:	C:\...gh Sensitivity DNA Assay_DEDAE00921_2022-09-01_12-23-18.xad	Modified:	9/1/2022 1:04:38 PM

Electropherogram Summary Continued ...

Y1_5mC

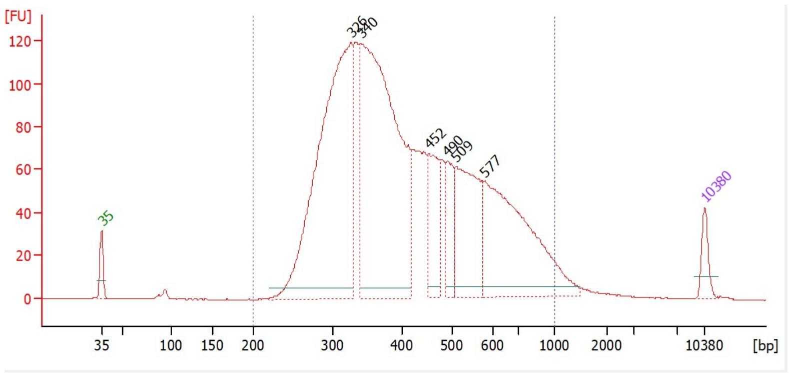

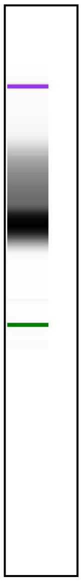

Overall Results for sample 9 : Y1_5mC

Number of peaks found:	6	Corr. Area 1:	2,601.8
Noise:	0.1		

Peak table for sample 9 : Y1_5mC

Peak		Size [bp]	Conc. [pg/µl]	Molarity [pmol/l]	Observations
1	◀	35	125.00	5,411.3	Lower Marker
2		326	2,546.57	11,839.8	
3		340	2,598.86	11,580.7	
4		452	370.06	1,239.3	
5		490	239.83	741.6	
6		509	674.55	2,009.8	
7		577	1,111.85	2,918.9	
8	▶	10,380	75.00	10.9	Upper Marker

Region table for sample 9 : Y1_5mC

From [bp]	To [bp]	Corr. Area	% of Total	Average Size [bp]	Size distribution in CV [%]	Conc. [pg/µl]	Molarity [pmol/l]	Color
200	1,000	2,601.8	98	433	33.7	8,507.69	33,808.5	■

 © Copyright 2003 - 2018 Agilent, Inc.

Assay Class: High Sensitivity DNA Assay
Data Path: C:\...gh Sensitivity DNA Assay_DEDAE00921_2022-09-01_12-23-18.xad

Created: 9/1/2022 12:23:17 PM
Modified: 9/1/2022 1:04:38 PM

Electropherogram Summary Continued ...

Y2_5mC

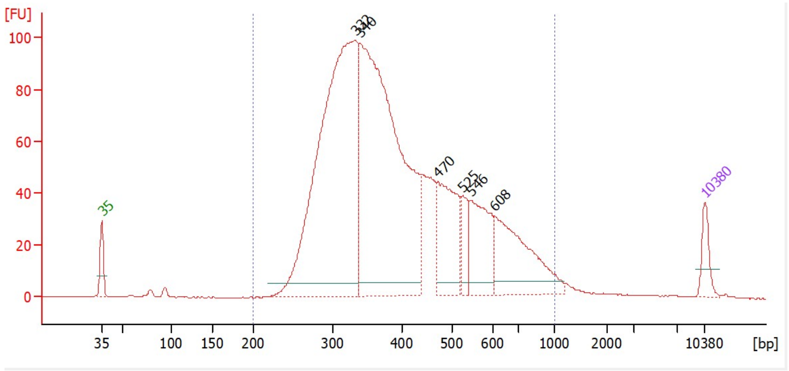

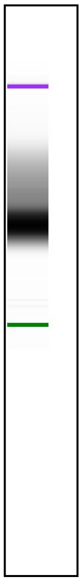

Overall Results for sample 10 : Y2_5mC

Number of peaks found:	6	Corr. Area 1:	1,934.6
Noise:	0.1		

Peak table for sample 10 : Y2_5mC

Peak		Size [bp]	Conc. [pg/µl]	Molarity [pmol/l]	Observations
1	◀	35	125.00	5,411.3	Lower Marker
2		332	2,554.71	11,673.1	
3		340	2,562.56	11,431.9	
4		470	455.13	1,467.4	
5		525	133.28	384.4	
6		546	360.82	1,001.1	
7		608	488.31	1,216.8	
8	▶	10,380	75.00	10.9	Upper Marker

Region table for sample 10 : Y2_5mC

From [bp]	To [bp]	Corr. Area	% of Total	Average Size [bp]	Size distribution in CV [%]	Conc. [pg/µl]	Molarity [pmol/l]	Color
200	1,000	1,934.6	98	415	32.6	6,943.03	28,409.9	

 © Copyright 2003 - 2018 Agilent, Inc.

Assay Class:	High Sensitivity DNA Assay	Created:	9/1/2022 12:23:17 PM
Data Path:	C:\...gh Sensitivity DNA Assay_DEDAE00921_2022-09-01_12-23-18.xad	Modified:	9/1/2022 1:04:38 PM

Electropherogram Summary Continued ...

Y3_5mC

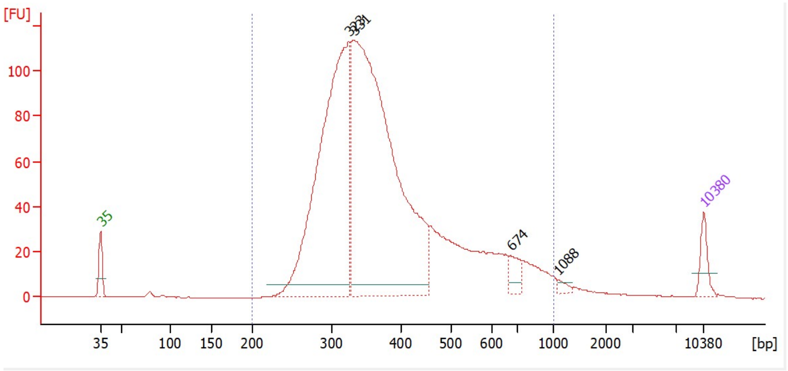

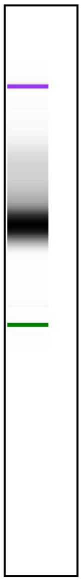

Overall Results for sample 11 : Y3_5mC

Number of peaks found:	4	Corr. Area 1:	1,812.8
Noise:	0.1		

Peak table for sample 11 : Y3_5mC

Peak		Size [bp]	Conc. [pg/µl]	Molarity [pmol/l]	Observations
1	◀	35	125.00	5,411.3	Lower Marker
2		323	2,336.81	10,949.1	
3		331	3,259.82	14,905.6	
4		674	78.77	177.1	
5		1,088	22.12	30.8	
6	▶	10,380	75.00	10.9	Upper Marker

Region table for sample 11 : Y3_5mC

From [bp]	To [bp]	Corr. Area	% of Total	Average Size [bp]	Size distribution in CV [%]	Conc. [pg/µl]	Molarity [pmol/l]	Co lor
200	1,000	1,812.8	98	395	32.8	6,608.65	28,007.1	■

 © Copyright 2003 - 2018 Agilent, Inc.

Assay Class: High Sensitivity DNA Assay
Data Path: C:\...gh Sensitivity DNA Assay_DEDAE00921_2022-09-01_12-23-18.xad

Created: 9/1/2022 12:23:17 PM
Modified: 9/1/2022 1:04:38 PM

Gel Image

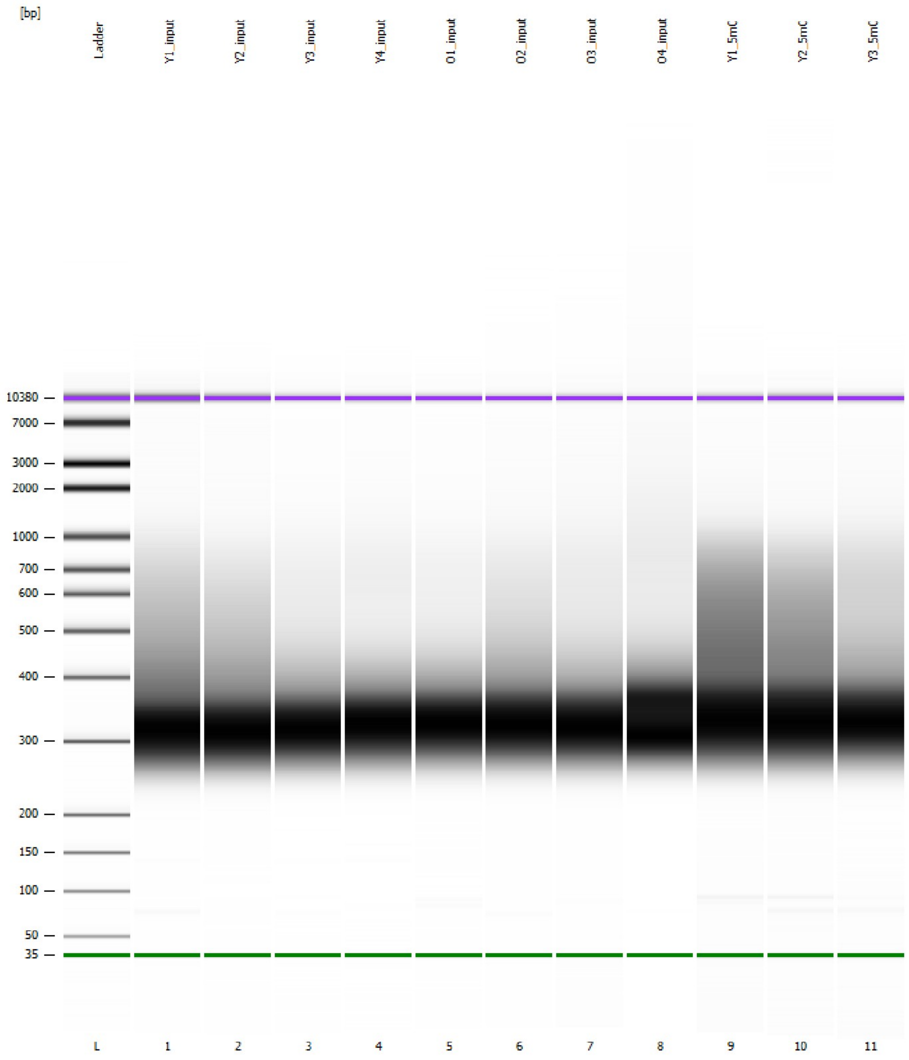

 © Copyright 2003 - 2018 Agilent, Inc.
