# Supplementary material for: Gene body DNA hydroxymethylation restricts the magnitude of transcriptional changes during aging
Source: Nat Commun. 2024 Jul 28;15:6357. doi: 10.1038/s41467-024-50725-y (PMC11284234; doi:10.1038/s41467-024-50725-y)
Supplement: Supplementary file 17 — Source Data [file 41467_2024_50725_MOESM17_ESM.zip › Source data_revisions_v3/Supp. Fig. 2/Fig.S2A_BioA/2100 expert_High Sensitivity DNA Assay_DEDAE00921_2022-09-01_13-20-53.pdf]

Assay Class:	High Sensitivity DNA Assay	Created:	9/1/2022 1:20:53 PM
Data Path:	C:\...gh Sensitivity DNA Assay_DEDAE00921_2022-09-01_13-20-53.xad	Modified:	9/1/2022 1:47:49 PM

Electrophoresis File Run Summary

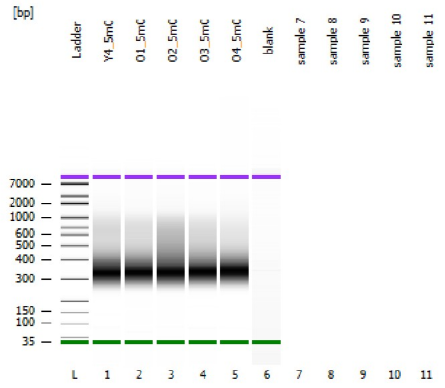

Instrument Information:

Instrument Name:	DEDAE00921	Firmware:	C.01.069
Serial#:	DEDAE00921	Type:	G2939B

Assay Information:

Assay Origin Path:	C:\Program Files (x86)\Agilent\2100 bioanalyzer\2100 expert\assays\dsDNA\High Sensitivity DNA.xsy
Assay Class:	High Sensitivity DNA Assay
Version:	1.03
Assay Comments:	Copyright © 2003-2010 Agilent Technologies

Chip Information:

Chip Lot #:

Reagent Kit Lot #:

Chip Comments:

Y4_5mC

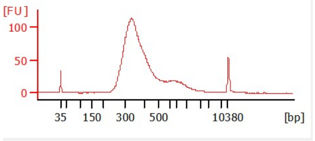

O1_5mC

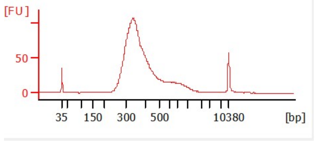

O2_5mC

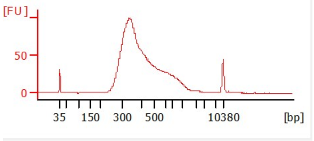

O3_5mC

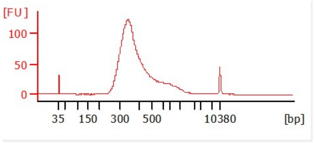

O4_5mC

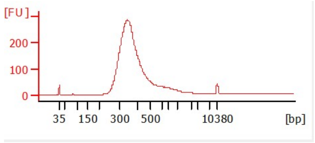

blank

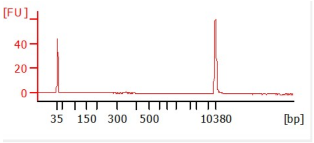

Assay Class: High Sensitivity DNA Assay
Data Path: C:\...gh Sensitivity DNA Assay_DEDAE00921_2022-09-01_13-20-53.xad

Created: 9/1/2022 1:20:53 PM
Modified: 9/1/2022 1:47:49 PM

Electrophoresis File Run Summary (Chip Summary)

Sample Name	Sample Comment	Rest. Digest	Status	Observation	Result Label	Result Color
Y4_5mC		☐	✓			
O1_5mC		☐	✓			
O2_5mC		☐	✓			
O3_5mC		☐	✓			
O4_5mC		☐	✓			
blank		☐	✓			
sample 7		☐				
sample 8		☐				
sample 9		☐				
sample 10		☐				
sample 11		☐				
Ladder		☐	✓			

Chip Lot #

Reagent Kit Lot #

Chip Comments :

© Copyright 2003 - 2018 Agilent, Inc.

Assay Class:	High Sensitivity DNA Assay	Created:	9/1/2022 1:20:53 PM
Data Path:	C:\...gh Sensitivity DNA Assay_DEDAE00921_2022-09-01_13-20-53.xad	Modified:	9/1/2022 1:47:49 PM

Electropherogram Summary

Ladder

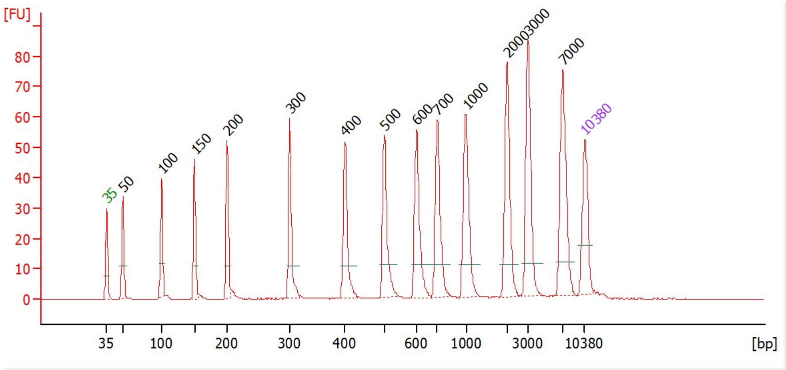

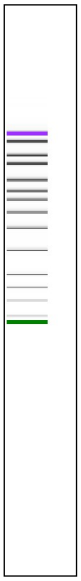

Overall Results for Ladder

Noise: 0.1

Peak table for Ladder

Peak		Size [bp]	Conc. [pg/µl]	Molarity [pmol/l]	Observations
1		35	125.00	5,411.3	Lower Marker
2		50	150.00	4,545.5	Ladder Peak
3		100	150.00	2,272.7	Ladder Peak
4		150	150.00	1,515.2	Ladder Peak
5		200	150.00	1,136.4	Ladder Peak
6		300	150.00	757.6	Ladder Peak
7		400	150.00	568.2	Ladder Peak
8		500	150.00	454.5	Ladder Peak
9		600	150.00	378.8	Ladder Peak
10		700	150.00	324.7	Ladder Peak
11		1,000	150.00	227.3	Ladder Peak
12		2,000	150.00	113.6	Ladder Peak
13		3,000	150.00	75.8	Ladder Peak
14		7,000	150.00	32.5	Ladder Peak
15		10,380	75.00	10.9	Upper Marker

 © Copyright 2003 - 2018 Agilent, Inc.

Assay Class: High Sensitivity DNA Assay
Data Path: C:\...gh Sensitivity DNA Assay_DEDAE00921_2022-09-01_13-20-53.xad

Created: 9/1/2022 1:20:53 PM
Modified: 9/1/2022 1:47:49 PM

Electropherogram Summary Continued ...

Y4_5mC

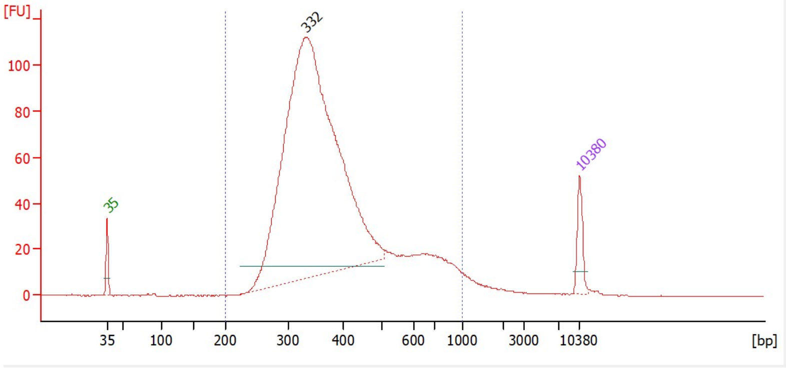

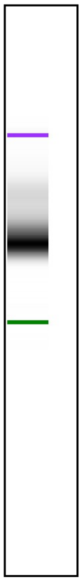

Overall Results for sample 1 : Y4_5mC

Number of peaks found: 1

Corr. Area 1: 1,749.9

Noise: 0.1

Peak table for sample 1 : Y4_5mC

Peak		Size [bp]	Conc. [pg/µl]	Molarity [pmol/l]	Observations
1	◀	35	125.00	5,411.3	Lower Marker
2		332	3,662.31	16,717.6	
3	▶	10,380	75.00	10.9	Upper Marker

Region table for sample 1 : Y4_5mC

From [bp]	To [bp]	Corr. Area	% of Total	Average Size [bp]	Size distribution in CV [%]	Conc. [pg/µl]	Molarity [pmol/l]	Co lor
200	1,000	1,749.9	97	400	33.1	4,700.81	19,670.0	■

© Copyright 2003 - 2018 Agilent, Inc.

Assay Class:	High Sensitivity DNA Assay	Created:	9/1/2022 1:20:53 PM
Data Path:	C:\...gh Sensitivity DNA Assay_DEDAE00921_2022-09-01_13-20-53.xad	Modified:	9/1/2022 1:47:49 PM

Electropherogram Summary Continued ...

O1_5mC

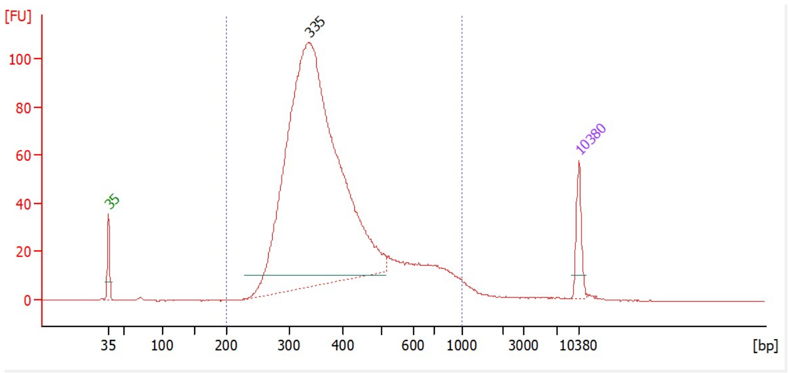

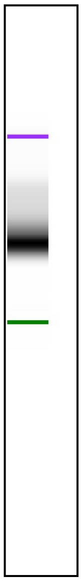

Overall Results for sample 2 : O1_5mC

Number of peaks found:	1	Corr. Area 1:	1,586.8
Noise:	0.1		

Peak table for sample 2 : O1_5mC

Peak		Size [bp]	Conc. [pg/µl]	Molarity [pmol/l]	Observations
1	◀	35	125.00	5,411.3	Lower Marker
2		335	3,254.25	14,701.6	
3	▶	10,380	75.00	10.9	Upper Marker

Region table for sample 2 : O1_5mC

From [bp]	To [bp]	Corr. Area	% of Total	Average Size [bp]	Size distribution in CV [%]	Conc. [pg/µl]	Molarity [pmol/l]	Color
200	1,000	1,586.8	97	398	32.0	4,001.83	16,716.9	■

 © Copyright 2003 - 2018 Agilent, Inc.

Assay Class: High Sensitivity DNA Assay
Data Path: C:\...gh Sensitivity DNA Assay_DEDAE00921_2022-09-01_13-20-53.xad
Created: 9/1/2022 1:20:53 PM
Modified: 9/1/2022 1:47:49 PM

Electropherogram Summary Continued ...

O2_5mC

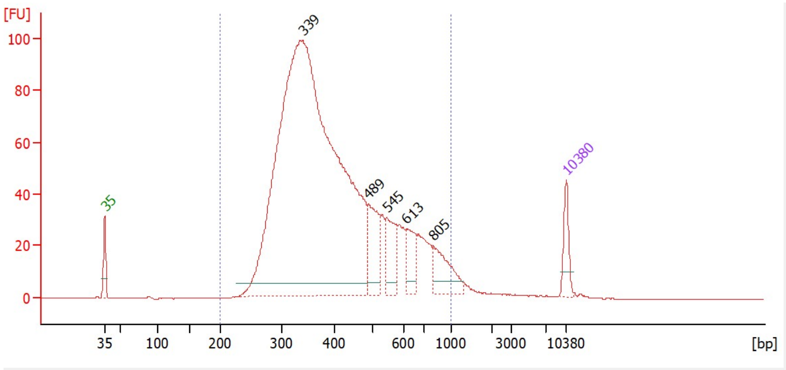

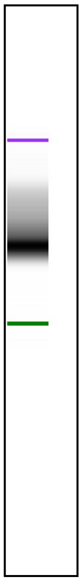

Overall Results for sample 3 : O2_5mC

Number of peaks found:	5	Corr. Area 1:	1,772.6
Noise:	0.1		

Peak table for sample 3 : O2_5mC

Peak		Size [bp]	Conc. [pg/µl]	Molarity [pmol/l]	Observations
1	◀	35	125.00	5,411.3	Lower Marker
2		339	4,577.90	20,463.3	
3		489	228.42	708.4	
4		545	175.81	488.8	
5		613	125.64	310.6	
6		805	162.48	305.7	
7	▶	10,380	75.00	10.9	Upper Marker

Region table for sample 3 : O2_5mC

From [bp]	To [bp]	Corr. Area	% of Total	Average Size [bp]	Size distribution in CV [%]	Conc. [pg/µl]	Molarity [pmol/l]	Color
200	1,000	1,772.6	97	423	33.5	5,575.84	22,427.3	■

 © Copyright 2003 - 2018 Agilent, Inc.

Assay Class: High Sensitivity DNA Assay
Data Path: C:\...gh Sensitivity DNA Assay_DEDAE00921_2022-09-01_13-20-53.xad

Created: 9/1/2022 1:20:53 PM
Modified: 9/1/2022 1:47:49 PM

Electropherogram Summary Continued ...

O3_5mC

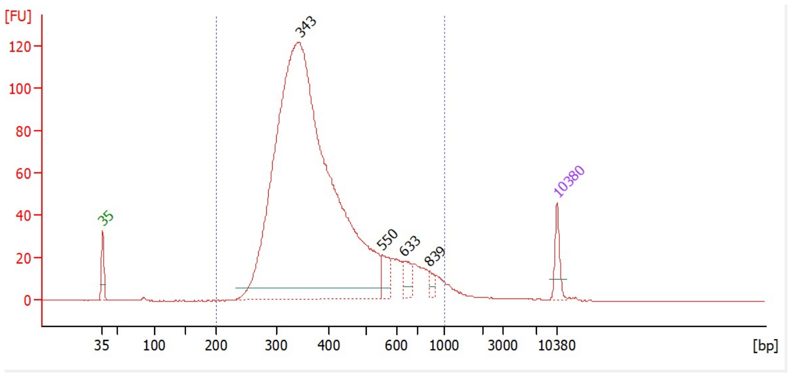

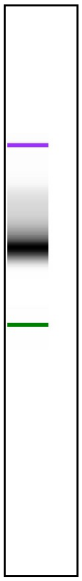

Overall Results for sample 4 : O3_5mC

Number of peaks found: 4
Corr. Area 1: 1,816.7
Noise: 0.1

Peak table for sample 4 : O3_5mC

Peak		Size [bp]	Conc. [pg/µl]	Molarity [pmol/l]	Observations
1	◄	35	125.00	5,411.3	Lower Marker
2		343	5,197.79	22,927.6	
3		550	90.43	249.0	
4		633	67.73	162.0	
5		839	31.21	56.4	
6	►	10,380	75.00	10.9	Upper Marker

Region table for sample 4 : O3_5mC

From [bp]	To [bp]	Corr. Area	% of Total	Average Size [bp]	Size distribution in CV [%]	Conc. [pg/µl]	Molarity [pmol/l]	Color
200	1,000	1,816.7	98	402	31.5	5,635.37	23,311.3	■

© Copyright 2003 - 2018 Agilent, Inc.

Assay Class: High Sensitivity DNA Assay
Data Path: C:\...gh Sensitivity DNA Assay_DEDAE00921_2022-09-01_13-20-53.xad
Created: 9/1/2022 1:20:53 PM
Modified: 9/1/2022 1:47:49 PM

Electropherogram Summary Continued ...

O4_5mC

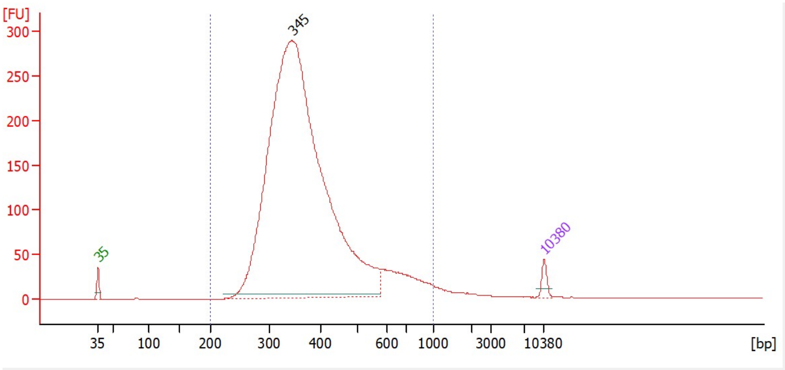

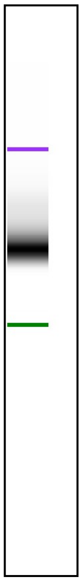

Overall Results for sample 5 : O4_5mC

Number of peaks found: 1
Corr. Area 1: 4,043.3
Noise: 0.1

Peak table for sample 5 : O4_5mC

Peak		Size [bp]	Conc. [pg/µl]	Molarity [pmol/l]	Observations
1	◀	35	125.00	5,411.3	Lower Marker
2		345	12,901.18	56,707.7	
3	▶	10,380	75.00	10.9	Upper Marker

Region table for sample 5 : O4_5mC

From [bp]	To [bp]	Corr. Area	% of Total	Average Size [bp]	Size distribution in CV [%]	Conc. [pg/µl]	Molarity [pmol/l]	Color
200	1,000	4,043.3	98	389	28.5	13,360.58	56,004.1	

© Copyright 2003 - 2018 Agilent, Inc.

Assay Class: High Sensitivity DNA Assay
Data Path: C:\...gh Sensitivity DNA Assay_DEDAE00921_2022-09-01_13-20-53.xad
Created: 9/1/2022 1:20:53 PM
Modified: 9/1/2022 1:47:49 PM

Electropherogram Summary Continued ...

blank

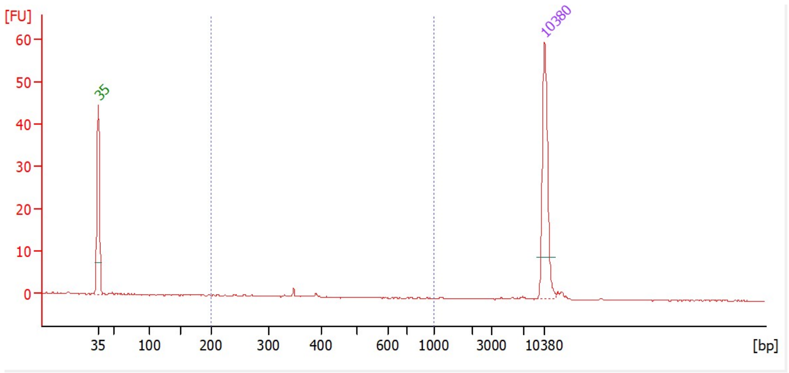

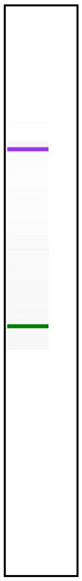

Overall Results for sample 6 : **blank**

Number of peaks found: 0
Corr. Area 1: 0.8
Noise: 0.1

Peak table for sample 6 : **blank**

Peak		Size [bp]	Conc. [pg/µl]	Molarity [pmol/l]	Observations
1	◀	35	125.00	5,411.3	Lower Marker
2	▶	10,380	75.00	10.9	Upper Marker

Region table for sample 6 : **blank**

From [bp]	To [bp]	Corr. Area	% of Total	Average Size [bp]	Size distribution in CV [%]	Conc. [pg/µl]	Molarity [pmol/l]	Co lor
200	1,000	0.8	23	357	7.4	1.72	7.3	■

© Copyright 2003 - 2018 Agilent, Inc.

Assay Class: High Sensitivity DNA Assay
Data Path: C:\...gh Sensitivity DNA Assay_DEDAE00921_2022-09-01_13-20-53.xad

Created: 9/1/2022 1:20:53 PM
Modified: 9/1/2022 1:47:49 PM

Gel Image

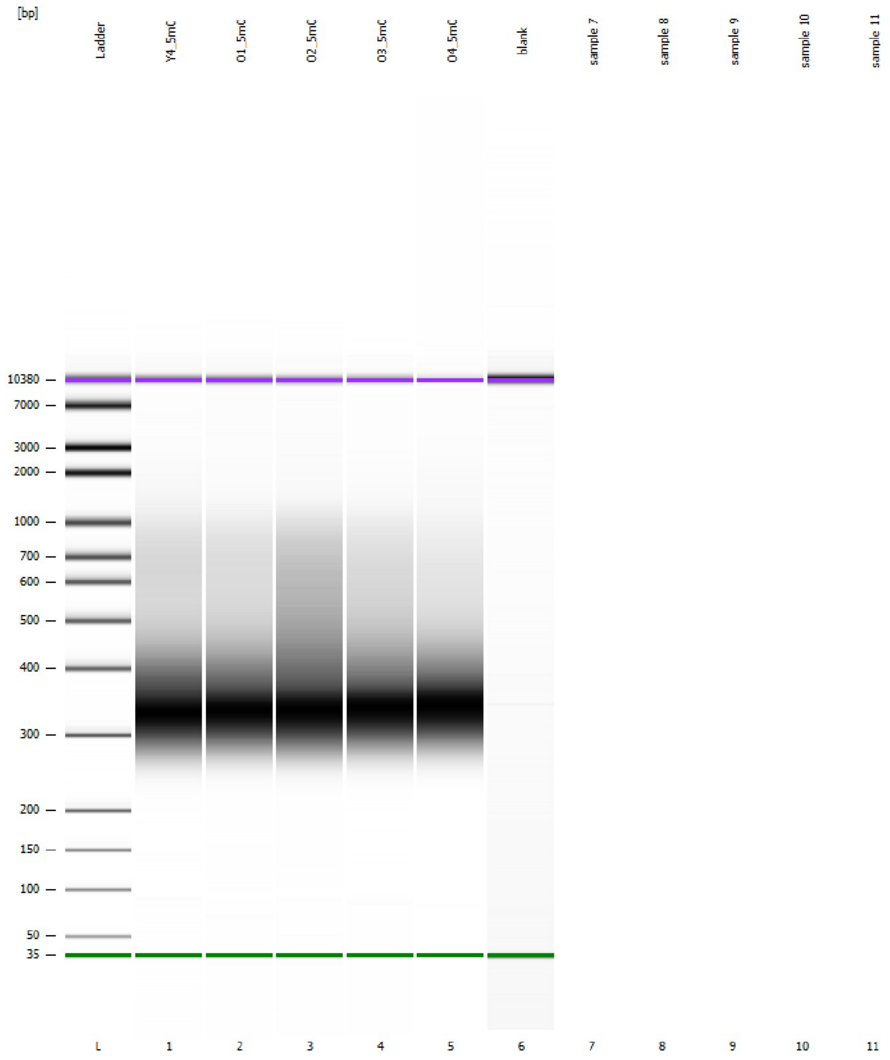

 © Copyright 2003 - 2018 Agilent, Inc.

Assay Class:	High Sensitivity DNA Assay	Created:	9/1/2022 1:20:53 PM
Data Path:	C:\...gh Sensitivity DNA Assay_DEDAE00921_2022-09-01_13-20-53.xad	Modified:	9/1/2022 1:47:49 PM

Invalid Samples

Sample 7 has not been run, no results available.

Sample 8 has not been run, no results available.

Sample 9 has not been run, no results available.

Sample 10 has not been run, no results available.

Sample 11 has not been run, no results available.

 © Copyright 2003 - 2018 Agilent, Inc.
