# Supplementary material for: Gene body DNA hydroxymethylation restricts the magnitude of transcriptional changes during aging
Source: Nat Commun. 2024 Jul 28;15:6357. doi: 10.1038/s41467-024-50725-y (PMC11284234; doi:10.1038/s41467-024-50725-y)
Supplement: Supplementary file 17 — Source Data [file 41467_2024_50725_MOESM17_ESM.zip › Source data_revisions_v3/Supp. Fig. 6/Fig.S6A_BioA/2100 expert_High Sensitivity DNA Assay_DEDAE00921_2022-02-25_18-27-18_hMeDIP_cerebellum_input.pdf]

Assay Class:	High Sensitivity DNA Assay	Created:	2/25/2022 6:27:18 PM
Data Path:	C:\..._DEDAE00921_2022-02-25_18-27-18_hMeDIP_cerebellum_input.xad	Modified:	2/25/2022 7:32:56 PM

Electrophoresis File Run Summary

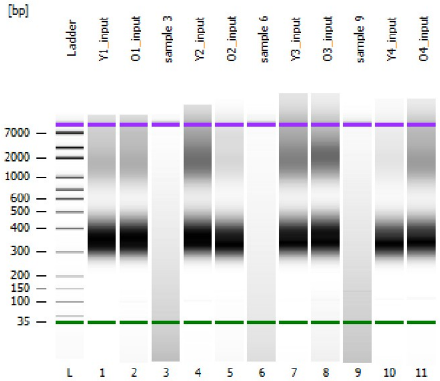

Instrument Information:

Instrument Name:	DEDAE00921	Firmware:	C.01.069
Serial#:	DEDAE00921	Type:	G2939B

Assay Information:

Assay Origin Path:	C:\Program Files (x86)\Agilent\2100 bioanalyzer\2100 expert\assays\dsDNA\High Sensitivity DNA.xsy
Assay Class:	High Sensitivity DNA Assay
Version:	1.03
Assay Comments:	Copyright © 2003-2010 Agilent Technologies

Chip Information:

Chip Lot #:

Reagent Kit Lot #:

Chip Comments:

Y1_input

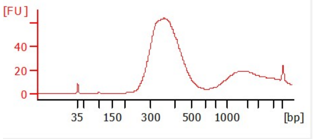

O1_input

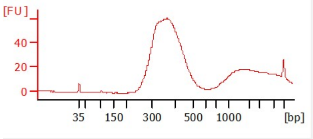

sample 3

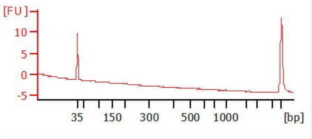

Y2_input

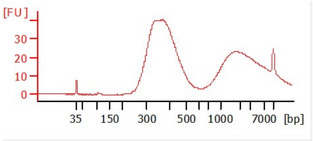

O2_input

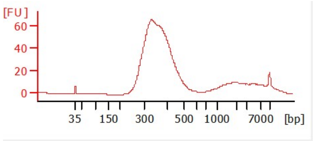

sample 6

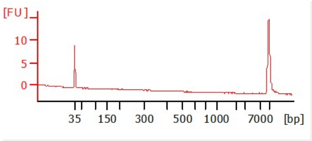

Y3_input

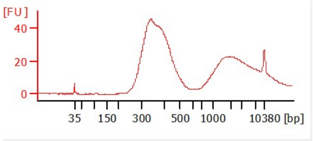

O3_input

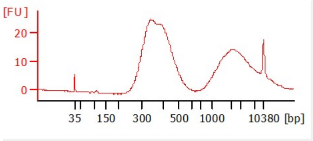

sample 9

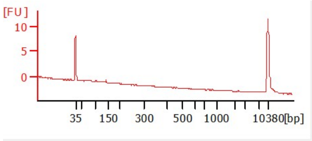

Y4_input

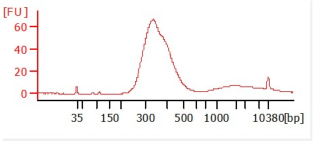

O4_input

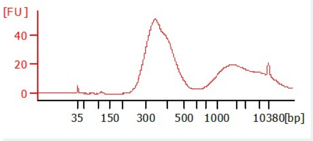

 © Copyright 2003 - 2018 Agilent, Inc.

Assay Class: High Sensitivity DNA Assay
Data Path: C:\..._DEDAE00921_2022-02-25_18-27-18_hMeDIP_cerebellum_input.xad
Created: 2/25/2022 6:27:18 PM
Modified: 2/25/2022 7:32:56 PM

Electrophoresis File Run Summary (Chip Summary)

Sample Name	Sample Comment	Rest. Digest	Status	Observation	Result Label	Result Color
Y1_input		☐	✓			
O1_input		☐	✓			
sample 3		☐	✓			
Y2_input		☐	✓			
O2_input		☐	✓			
sample 6		☐	✓			
Y3_input		☐	✓			
O3_input		☐	✓			
sample 9		☐	✓			
Y4_input		☐	✓			
O4_input		☐	✓			
Ladder		☐	✓			

Chip Lot # **Reagent Kit Lot #**

Chip Comments :

© Copyright 2003 - 2018 Agilent, Inc.

Assay Class: High Sensitivity DNA Assay
Data Path: C:\..._DEDAE00921_2022-02-25_18-27-18_hMeDIP_cerebellum_input.xad

Created: 2/25/2022 6:27:18 PM
Modified: 2/25/2022 7:32:56 PM

Electropherogram Summary

Ladder

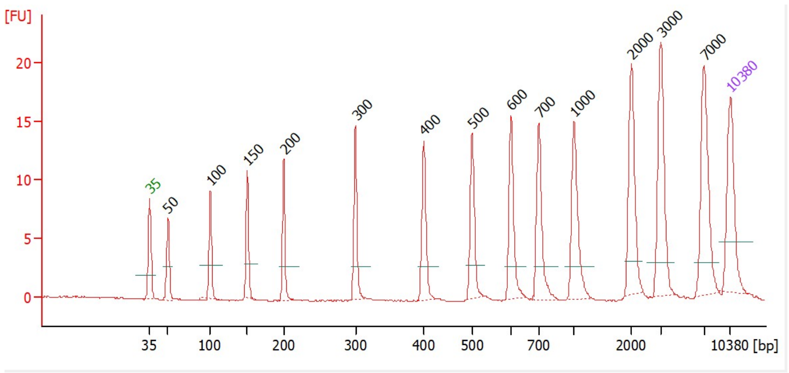

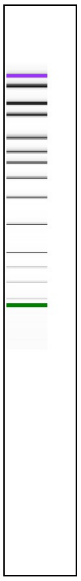

Overall Results for Ladder

Noise: 0.0

Peak table for Ladder

Peak		Size [bp]	Conc. [pg/µl]	Molarity [pmol/l]	Observations
1		35	125.00	5,411.3	Lower Marker
2		50	150.00	4,545.5	Ladder Peak
3		100	150.00	2,272.7	Ladder Peak
4		150	150.00	1,515.2	Ladder Peak
5		200	150.00	1,136.4	Ladder Peak
6		300	150.00	757.6	Ladder Peak
7		400	150.00	568.2	Ladder Peak
8		500	150.00	454.5	Ladder Peak
9		600	150.00	378.8	Ladder Peak
10		700	150.00	324.7	Ladder Peak
11		1,000	150.00	227.3	Ladder Peak
12		2,000	150.00	113.6	Ladder Peak
13		3,000	150.00	75.8	Ladder Peak
14		7,000	150.00	32.5	Ladder Peak
15		10,380	75.00	10.9	Upper Marker

© Copyright 2003 - 2018 Agilent, Inc.

Assay Class: High Sensitivity DNA Assay
Data Path: C:\..._DEDAE00921_2022-02-25_18-27-18_hMeDIP_cerebellum_input.xad

Created: 2/25/2022 6:27:18 PM
Modified: 2/25/2022 7:32:56 PM

Electropherogram Summary Continued ...

Y1_input

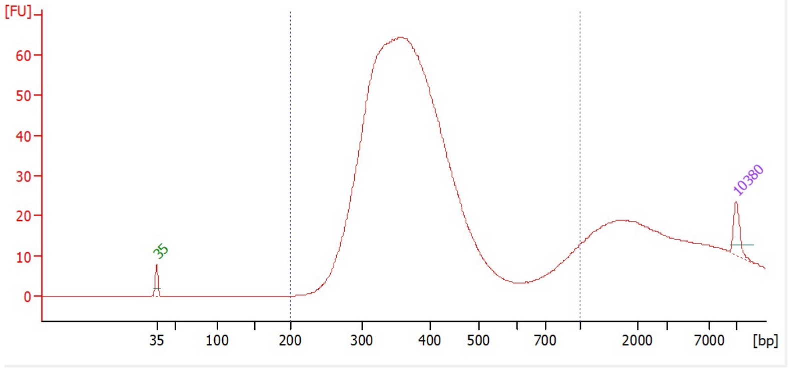

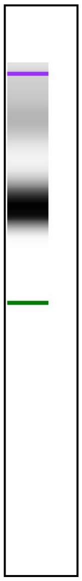

Overall Results for sample 1 : Y1_input

Number of peaks found: 0

Corr. Area 1: 985.3

Noise: 0.0

Peak table for sample 1 : Y1_input

Peak	Size [bp]	Conc. [pg/µl]	Molarity [pmol/l]	Observations
1	35	125.00	5,411.3	Lower Marker
2	10,380	75.00	10.9	Upper Marker

Region table for sample 1 : Y1_input

From [bp]	To [bp]	Corr. Area	% of Total	Average Size [bp]	Size distribution in CV [%]	Conc. [pg/µl]	Molarity [pmol/l]	Color
200	1,000	985.3	86	376	24.8	9,974.60	42,199.1	

© Copyright 2003 - 2018 Agilent, Inc.

Assay Class: High Sensitivity DNA Assay
Data Path: C:\..._DEDAE00921_2022-02-25_18-27-18_hMeDIP_cerebellum_input.xad
Created: 2/25/2022 6:27:18 PM
Modified: 2/25/2022 7:32:56 PM

Electropherogram Summary Continued ...

O1_input

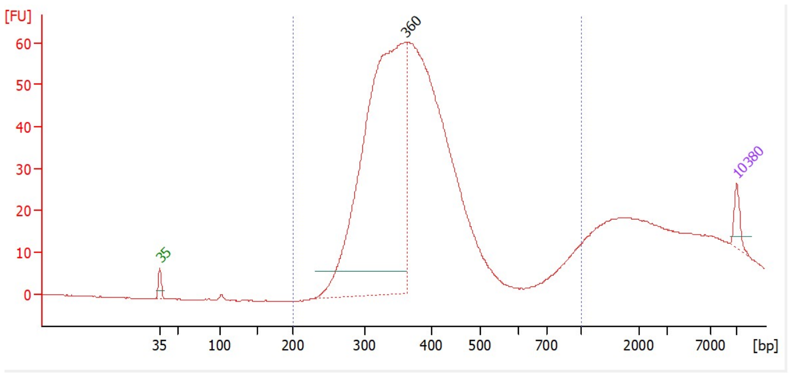

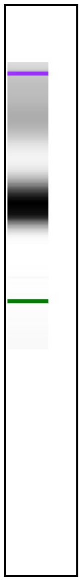

Overall Results for sample 2 : O1_input

Number of peaks found:	1	Corr. Area 1:	911.5
Noise:	0.1		

Peak table for sample 2 : O1_input

Peak	Size [bp]	Conc. [pg/µl]	Molarity [pmol/l]	Observations
1	35	125.00	5,411.3	Lower Marker
2	360	4,978.38	20,933.1	
3	10,380	75.00	10.9	Upper Marker

Region table for sample 2 : O1_input

From [bp]	To [bp]	Corr. Area	% of Total	Average Size [bp]	Size distribution in CV [%]	Conc. [pg/µl]	Molarity [pmol/l]	Color
200	1,000	911.5	84	376	23.9	9,030.56	38,048.3	

© Copyright 2003 - 2018 Agilent, Inc.

Assay Class: High Sensitivity DNA Assay
Data Path: C:\..._DEDAE00921_2022-02-25_18-27-18_hMeDIP_cerebellum_input.xad
Created: 2/25/2022 6:27:18 PM
Modified: 2/25/2022 7:32:56 PM

Electropherogram Summary Continued ...

sample 3

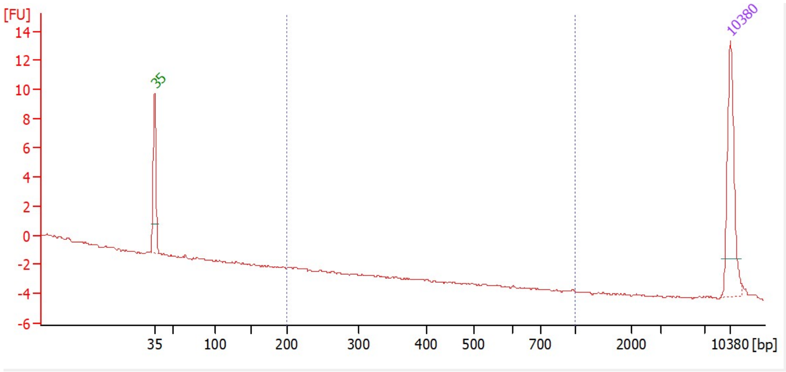

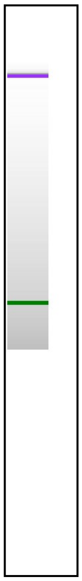

Overall Results for sample 3 : sample 3

Number of peaks found: 0
Corr. Area 1: 0.0
Noise: 0.0

Peak table for sample 3 : sample 3

Peak	Size [bp]	Conc. [pg/µl]	Molarity [pmol/l]	Observations
1	35	125.00	5,411.3	Lower Marker
2	10,380	75.00	10.9	Upper Marker

Region table for sample 3 : sample 3

From [bp]	To [bp]	Corr. Area	% of Total	Average Size [bp]	Size distribution in CV [%]	Conc. [pg/µl]	Molarity [pmol/l]	Co lor
200	1,000	0.0	0	0	0.0	0.00	0.0	

© Copyright 2003 - 2018 Agilent, Inc.

Assay Class: High Sensitivity DNA Assay
Data Path: C:\..._DEDAE00921_2022-02-25_18-27-18_hMeDIP_cerebellum_input.xad

Created: 2/25/2022 6:27:18 PM
Modified: 2/25/2022 7:32:56 PM

Electropherogram Summary Continued ...

Y2_input

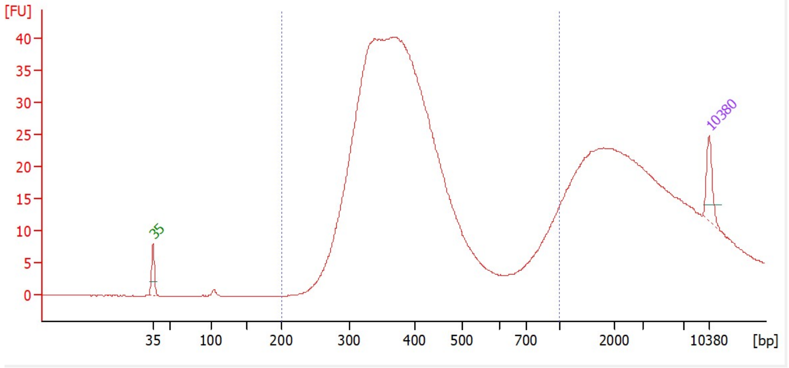

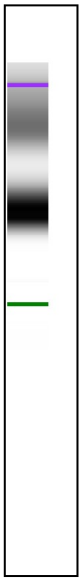

Overall Results for sample 4 : Y2 input

Number of peaks found: 0
Corr. Area 1: 630.3
Noise: 0.0

Peak table for sample 4 : Y2 input

Peak	Size [bp]	Conc. [pg/µl]	Molarity [pmol/l]	Observations
1	35	125.00	5,411.3	Lower Marker
2	10,380	75.00	10.9	Upper Marker

Region table for sample 4 : Y2 input

From [bp]	To [bp]	Corr. Area	% of Total	Average Size [bp]	Size distribution in CV [%]	Conc. [pg/µl]	Molarity [pmol/l]	Co lor
200	1,000	630.3	70	398	31.1	6,915.96	28,321.6	

 © Copyright 2003 - 2018 Agilent, Inc.

Assay Class:	High Sensitivity DNA Assay	Created:	2/25/2022 6:27:18 PM
Data Path:	C:\..._DEDAE00921_2022-02-25_18-27-18_hMeDIP_cerebellum_input.xad	Modified:	2/25/2022 7:32:56 PM

Electropherogram Summary Continued ...

O2_input

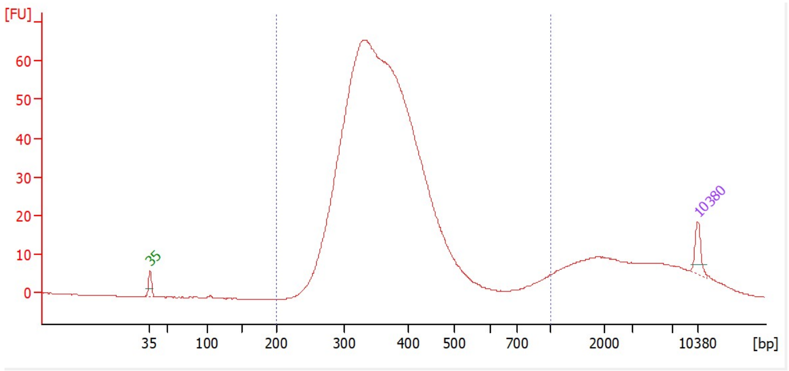

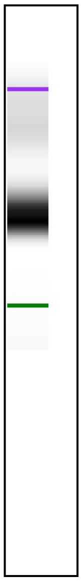

Overall Results for sample 5 : **O2_input**

Number of peaks found:	0	Corr. Area 1:	1,065.3
Noise:	0.1		

Peak table for sample 5 : **O2_input**

Peak		Size [bp]	Conc. [pg/µl]	Molarity [pmol/l]	Observations
1	◀	35	125.00	5,411.3	Lower Marker
2	▶	10,380	75.00	10.9	Upper Marker

Region table for sample 5 : **O2_input**

From [bp]	To [bp]	Corr. Area	% of Total	Average Size [bp]	Size distribution in CV [%]	Conc. [pg/µl]	Molarity [pmol/l]	Color
200	1,000	1,065.3	87	374	24.7	11,347.87	48,442.3	■

 © Copyright 2003 - 2018 Agilent, Inc.

Assay Class: High Sensitivity DNA Assay
Data Path: C:\..._DEDAE00921_2022-02-25_18-27-18_hMeDIP_cerebellum_input.xad

Created: 2/25/2022 6:27:18 PM
Modified: 2/25/2022 7:32:56 PM

Electropherogram Summary Continued ...

sample 6

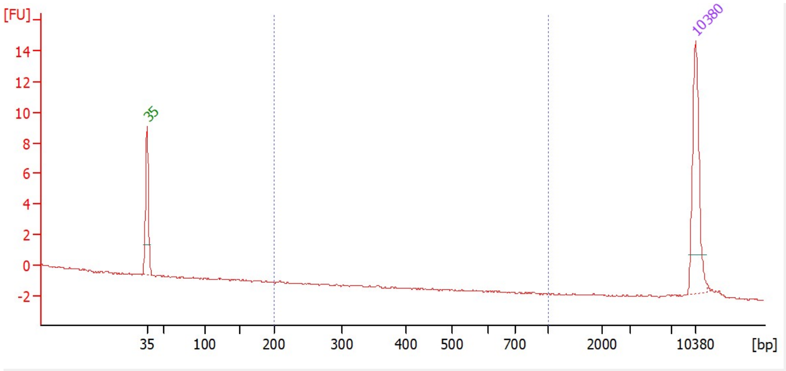

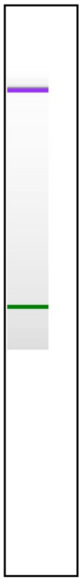

Overall Results for sample 6 : sample 6

Number of peaks found: 0

Corr. Area 1: 0.0

Noise: 0.0

Peak table for sample 6 : sample 6

Peak		Size [bp]	Conc. [pg/µl]	Molarity [pmol/l]	Observations
1	◀	35	125.00	5,411.3	Lower Marker
2	▶	10,380	75.00	10.9	Upper Marker

Region table for sample 6 : sample 6

From [bp]	To [bp]	Corr. Area	% of Total	Average Size [bp]	Size distribution in CV [%]	Conc. [pg/µl]	Molarity [pmol/l]	Co lor
200	1,000	0.0	0	0	0.0	0.00	0.0	■

© Copyright 2003 - 2018 Agilent, Inc.

Assay Class:	High Sensitivity DNA Assay	Created:	2/25/2022 6:27:18 PM
Data Path:	C:\..._DEDAE00921_2022-02-25_18-27-18_hMeDIP_cerebellum_input.xad	Modified:	2/25/2022 7:32:56 PM

Electropherogram Summary Continued ...

Y3_input

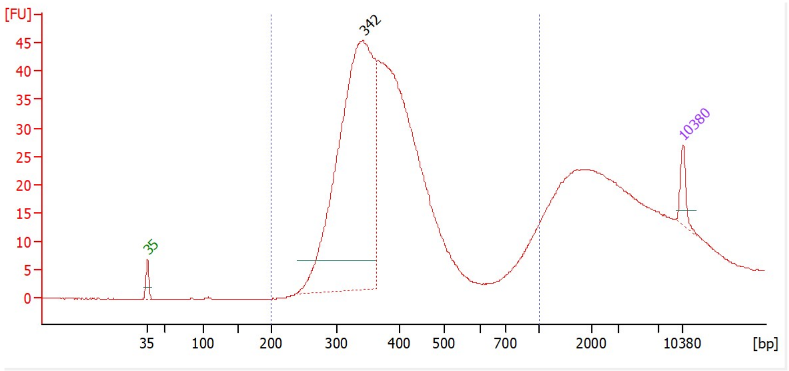

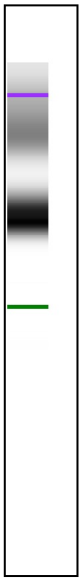

Overall Results for sample 7 : Y3_input

Number of peaks found:	1	Corr. Area 1:	677.5
Noise:	0.1		

Peak table for sample 7 : Y3_input

Peak		Size [bp]	Conc. [pg/µl]	Molarity [pmol/l]	Observations
1	◀	35	125.00	5,411.3	Lower Marker
2		342	3,563.87	15,811.7	
3	▶	10,380	75.00	10.9	Upper Marker

Region table for sample 7 : Y3_input

From [bp]	To [bp]	Corr. Area	% of Total	Average Size [bp]	Size distribution in CV [%]	Conc. [pg/µl]	Molarity [pmol/l]	Color
200	1,000	677.5	71	391	29.9	6,899.95	28,553.5	■

 © Copyright 2003 - 2018 Agilent, Inc.

Assay Class:	High Sensitivity DNA Assay	Created:	2/25/2022 6:27:18 PM
Data Path:	C:\..._DEDAE00921_2022-02-25_18-27-18_hMeDIP_cerebellum_input.xad	Modified:	2/25/2022 7:32:56 PM

Electropherogram Summary Continued ...

O3_input

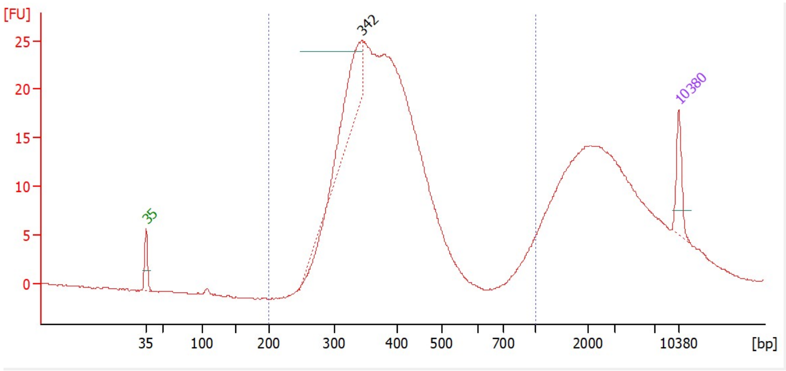

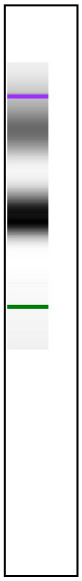

Overall Results for sample 8 : O3_input

Number of peaks found:	1	Corr. Area 1:	403.4
Noise:	0.1		

Peak table for sample 8 : O3_input

Peak		Size [bp]	Conc. [pg/µl]	Molarity [pmol/l]	Observations
1	◀	35	125.00	5,411.3	Lower Marker
2		342	381.75	1,688.8	
3	▶	10,380	75.00	10.9	Upper Marker

Region table for sample 8 : O3_input

From [bp]	To [bp]	Corr. Area	% of Total	Average Size [bp]	Size distribution in CV [%]	Conc. [pg/µl]	Molarity [pmol/l]	Color
200	1,000	403.4	68	389	26.5	4,808.57	19,764.9	

© Copyright 2003 - 2018 Agilent, Inc.

Assay Class: High Sensitivity DNA Assay
Data Path: C:\..._DEDAE00921_2022-02-25_18-27-18_hMeDIP_cerebellum_input.xad

Created: 2/25/2022 6:27:18 PM
Modified: 2/25/2022 7:32:56 PM

Electropherogram Summary Continued ...

sample 9

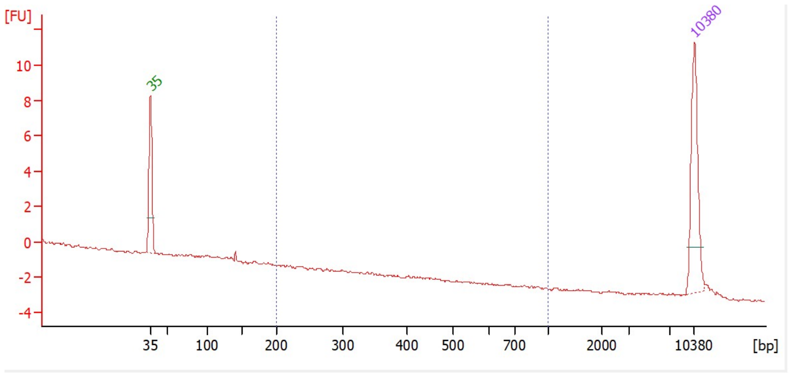

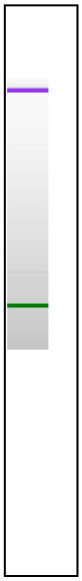

Overall Results for sample 9 : sample 9

Number of peaks found: 0 Corr. Area 1: 0.0

Noise: 0.1

Peak table for sample 9 : sample 9

Peak		Size [bp]	Conc. [pg/µl]	Molarity [pmol/l]	Observations
1	◀	35	125.00	5,411.3	Lower Marker
2	▶	10,380	75.00	10.9	Upper Marker

Region table for sample 9 : sample 9

From [bp]	To [bp]	Corr. Area	% of Total	Average Size [bp]	Size distribution in CV [%]	Conc. [pg/µl]	Molarity [pmol/l]	Color
200	1,000	0.0	0	0	0.0	0.00	0.0	

 © Copyright 2003 - 2018 Agilent, Inc.

Assay Class: High Sensitivity DNA Assay
Data Path: C:\..._DEDAE00921_2022-02-25_18-27-18_hMeDIP_cerebellum_input.xad

Created: 2/25/2022 6:27:18 PM
Modified: 2/25/2022 7:32:56 PM

Electropherogram Summary Continued ...

Y4_input

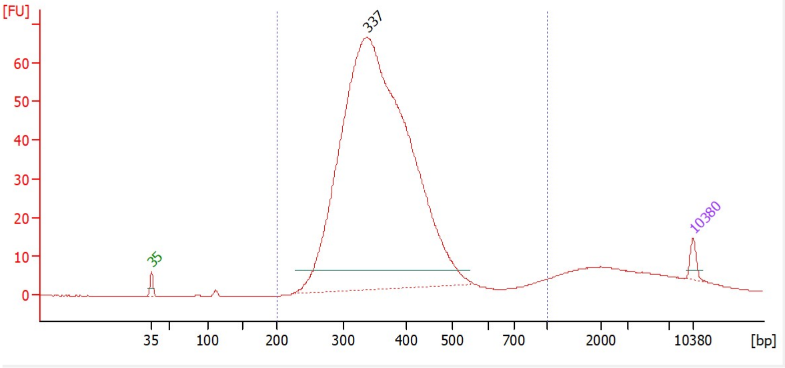

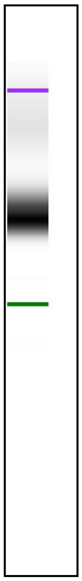

Overall Results for sample 10 : Y4_input

Number of peaks found: 1
Corr. Area 1: 990.8
Noise: 0.1

Peak table for sample 10 : Y4_input

Peak		Size [bp]	Conc. [pg/µl]	Molarity [pmol/l]	Observations
1	◀	35	125.00	5,411.3	Lower Marker
2		337	12,375.21	55,626.4	
3	▶	10,380	75.00	10.9	Upper Marker

Region table for sample 10 : Y4_input

From [bp]	To [bp]	Corr. Area	% of Total	Average Size [bp]	Size distribution in CV [%]	Conc. [pg/µl]	Molarity [pmol/l]	Co lor
200	1,000	990.8	91	368	23.0	12,651.03	54,590.8	

© Copyright 2003 - 2018 Agilent, Inc.

Assay Class: High Sensitivity DNA Assay
Data Path: C:\..._DEDAE00921_2022-02-25_18-27-18_hMeDIP_cerebellum_input.xad

Created: 2/25/2022 6:27:18 PM
Modified: 2/25/2022 7:32:56 PM

Electropherogram Summary Continued ...

O4_input

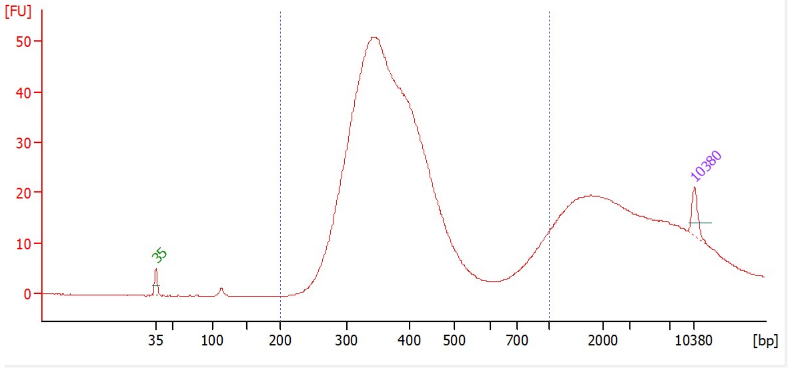

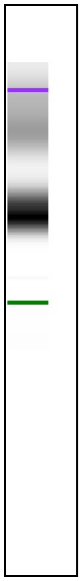

Overall Results for sample 11 : O4_input

Number of peaks found:	0	Corr. Area 1:	742.7
Noise:	0.1		

Peak table for sample 11 : O4_input

Peak		Size [bp]	Conc. [pg/µl]	Molarity [pmol/l]	Observations
1		35	125.00	5,411.3	Lower Marker
2		10,380	75.00	10.9	Upper Marker

Region table for sample 11 : O4_input

From [bp]	To [bp]	Corr. Area	% of Total	Average Size [bp]	Size distribution in CV [%]	Conc. [pg/µl]	Molarity [pmol/l]	Color
200	1,000	742.7	75	390	31.0	10,724.96	44,736.2	

© Copyright 2003 - 2018 Agilent, Inc.

Assay Class: High Sensitivity DNA Assay
Data Path: C:\..._DEDAE00921_2022-02-25_18-27-18_hMeDIP_cerebellum_input.xad

Created: 2/25/2022 6:27:18 PM
Modified: 2/25/2022 7:32:56 PM

Gel Image

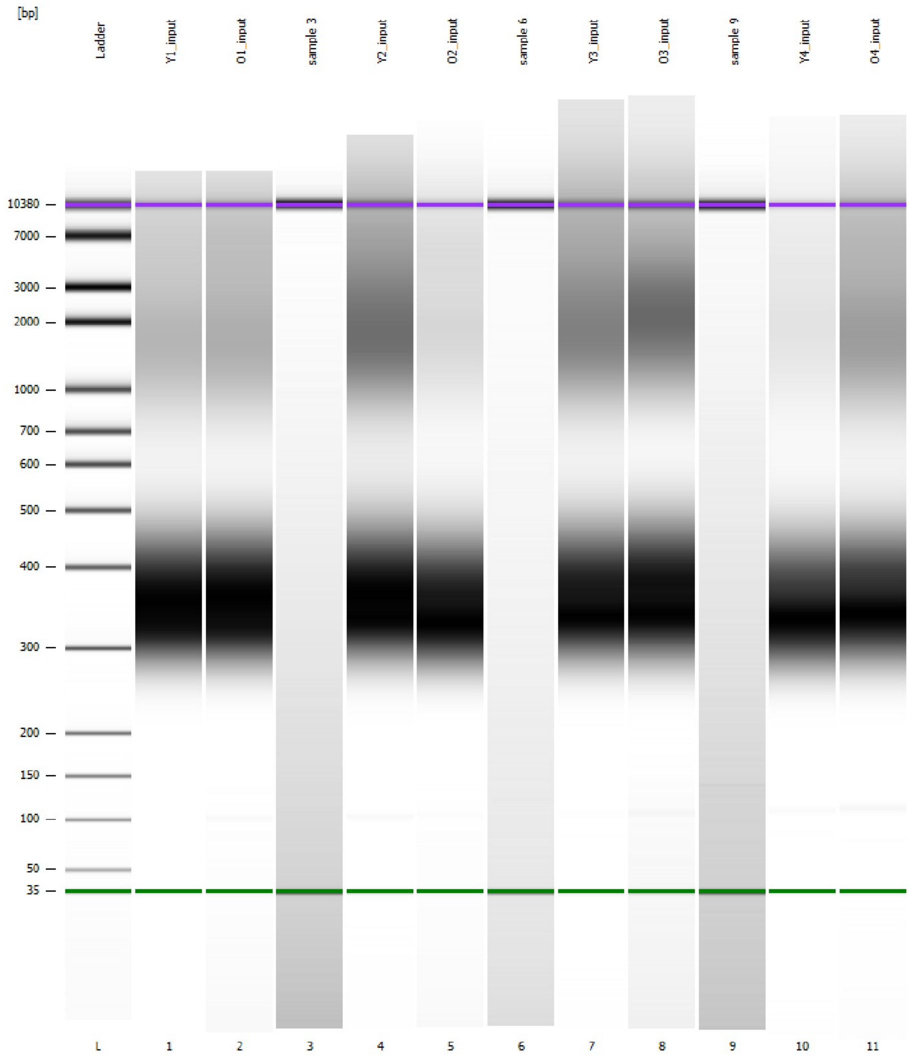

 © Copyright 2003 - 2018 Agilent, Inc.
