# Supplementary material for: Gene body DNA hydroxymethylation restricts the magnitude of transcriptional changes during aging
Source: Nat Commun. 2024 Jul 28;15:6357. doi: 10.1038/s41467-024-50725-y (PMC11284234; doi:10.1038/s41467-024-50725-y)
Supplement: Supplementary file 17 — Source Data [file 41467_2024_50725_MOESM17_ESM.zip › Source data_revisions_v3/Supp. Fig. 6/Fig.S6A_BioA/2100 expert_High Sensitivity DNA Assay_DEDAE00921_2022-02-25_19-55-41_hMeDIP_cerebellum_IP.pdf]

Assay Class: High Sensitivity DNA Assay
Data Path: C:\...say_DEDAE00921_2022-02-25_19-55-41_hMeDIP_cerebellum_IP.xad

Created: 2/25/2022 7:55:40 PM
Modified: 2/25/2022 8:44:45 PM

Electrophoresis File Run Summary

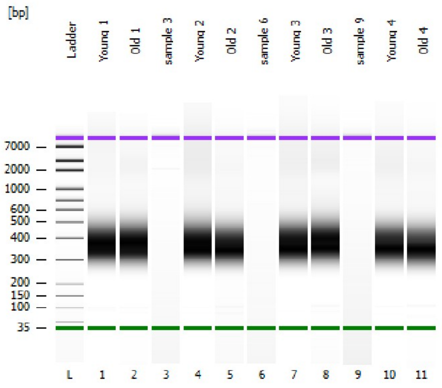

Instrument Information:

Instrument Name:	DEDAE00921	Firmware:	C.01.069
Serial#:	DEDAE00921	Type:	G2939B

Assay Information:

Assay Origin Path: C:\Program Files (x86)\Agilent\2100 bioanalyzer\2100 expert\assays\dsDNA\High Sensitivity DNA.xsy

Assay Class: High Sensitivity DNA Assay

Version: 1.03

Assay Comments: Copyright © 2003-2010 Agilent Technologies

Chip Information:

Chip Lot #:

Reagent Kit Lot #:

Chip Comments:

Young 1

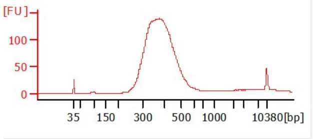

Old 1

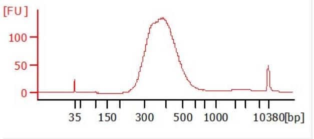

sample 3

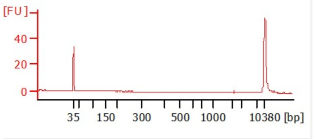

Young 2

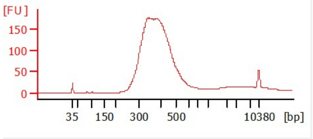

Old 2

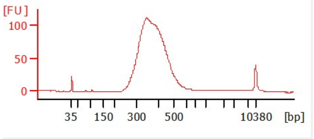

sample 6

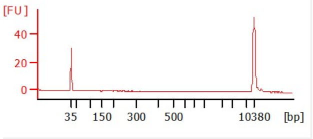

Young 3

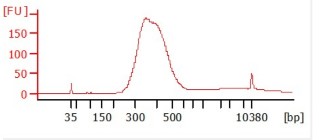

Old 3

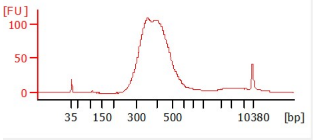

sample 9

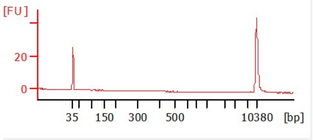

Young 4

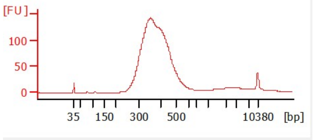

Old 4

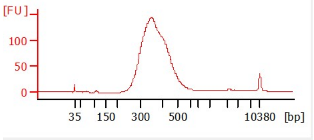

 © Copyright 2003 - 2018 Agilent, Inc.

Assay Class: High Sensitivity DNA Assay
Data Path: C:\...say_DEDAE00921_2022-02-25_19-55-41_hMeDIP_cerebellum_IP.xad
Created: 2/25/2022 7:55:40 PM
Modified: 2/25/2022 8:44:45 PM

Electrophoresis File Run Summary (Chip Summary)

Sample Name	Sample Comment	Rest. Digest	Status	Observation	Result Label	Result Color
Young 1		☐	✓			
Old 1		☐	✓			
sample 3		☐	✓			
Young 2		☐	✓			
Old 2		☐	✓			
sample 6		☐	✓			
Young 3		☐	✓			
Old 3		☐	✓			
sample 9		☐	✓			
Young 4		☐	✓			
Old 4		☐	✓			
Ladder		☐	✓			

Chip Lot # **Reagent Kit Lot #**

Chip Comments :

 © Copyright 2003 - 2018 Agilent, Inc.

Assay Class: High Sensitivity DNA Assay
Data Path: C:\...say_DEDAE00921_2022-02-25_19-55-41_hMeDIP_cerebellum_IP.xad

Created: 2/25/2022 7:55:40 PM
Modified: 2/25/2022 8:44:45 PM

Electropherogram Summary

Ladder

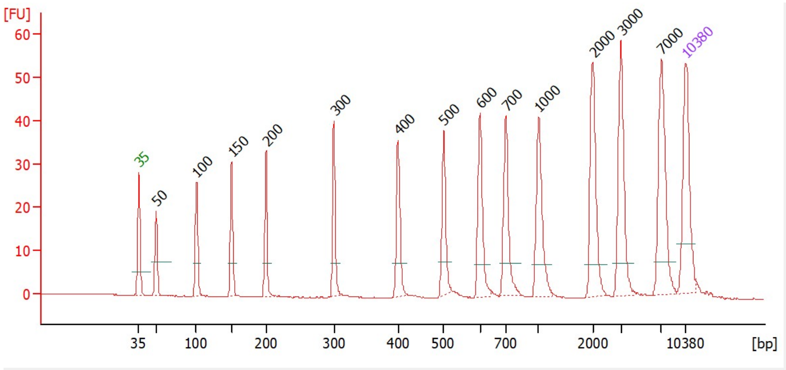

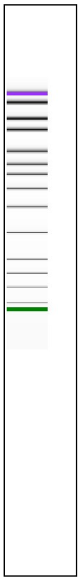

Overall Results for Ladder

Noise: 0.1

Peak table for Ladder

Peak		Size [bp]	Conc. [pg/µl]	Molarity [pmol/l]	Observations
1	◄	35	125.00	5,411.3	Lower Marker
2		50	150.00	4,545.5	Ladder Peak
3		100	150.00	2,272.7	Ladder Peak
4		150	150.00	1,515.2	Ladder Peak
5		200	150.00	1,136.4	Ladder Peak
6		300	150.00	757.6	Ladder Peak
7		400	150.00	568.2	Ladder Peak
8		500	150.00	454.5	Ladder Peak
9		600	150.00	378.8	Ladder Peak
10		700	150.00	324.7	Ladder Peak
11		1,000	150.00	227.3	Ladder Peak
12		2,000	150.00	113.6	Ladder Peak
13		3,000	150.00	75.8	Ladder Peak
14		7,000	150.00	32.5	Ladder Peak
15	►	10,380	75.00	10.9	Upper Marker

 © Copyright 2003 - 2018 Agilent, Inc.

Assay Class: High Sensitivity DNA Assay
Data Path: C:\...say_DEDAE00921_2022-02-25_19-55-41_hMeDIP_cerebellum_IP.xad

Created: 2/25/2022 7:55:40 PM
Modified: 2/25/2022 8:44:45 PM

Electropherogram Summary Continued ...

Young 1

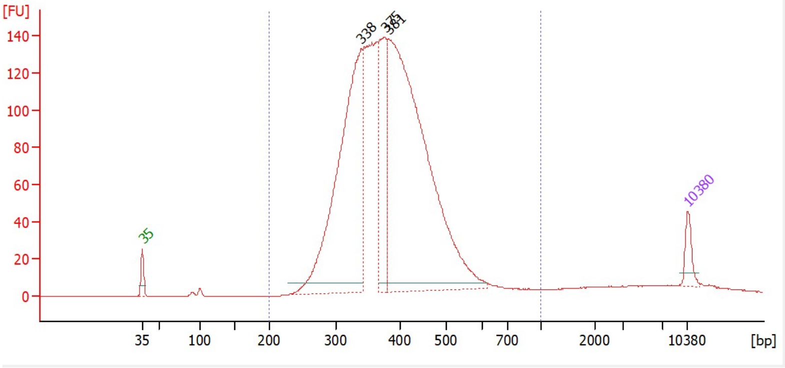

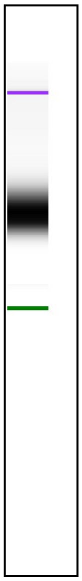

Overall Results for sample 1 : Young 1

Number of peaks found: 3

Corr. Area 1: 2,368.3

Noise: 0.1

Peak table for sample 1 : Young 1

Peak		Size [bp]	Conc. [pg/µl]	Molarity [pmol/l]	Observations
1	◀	35	125.00	5,411.3	Lower Marker
2		338	2,186.50	9,801.9	
3		375	669.40	2,705.9	
4		381	3,322.39	13,223.0	
5	▶	10,380	75.00	10.9	Upper Marker

Region table for sample 1 : Young 1

From [bp]	To [bp]	Corr. Area	% of Total	Average Size [bp]	Size distribution in CV [%]	Conc. [pg/µl]	Molarity [pmol/l]	Co lor
200	1,000	2,368.3	97	387	19.2	7,482.54	30,507.1	■

© Copyright 2003 - 2018 Agilent, Inc.

Assay Class: High Sensitivity DNA Assay
Data Path: C:\...say_DEDAE00921_2022-02-25_19-55-41_hMeDIP_cerebellum_IP.xad

Created: 2/25/2022 7:55:40 PM
Modified: 2/25/2022 8:44:45 PM

Electropherogram Summary Continued ...

Old 1

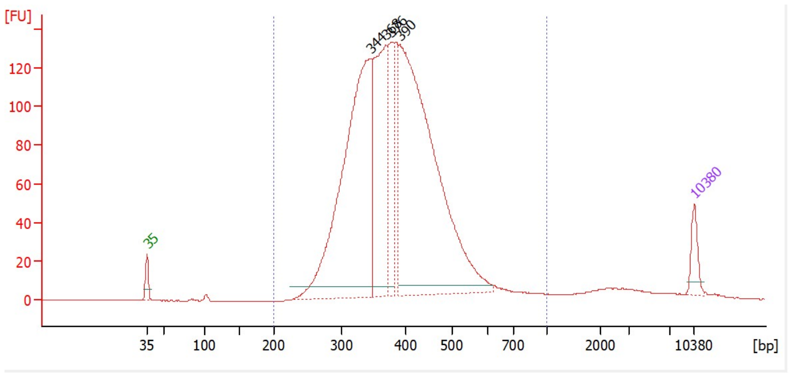

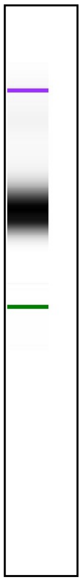

Overall Results for sample 2 : Old 1

Number of peaks found: 4

Corr. Area 1: 2,378.8

Noise: 0.1

Peak table for sample 2 : Old 1

Peak		Size [bp]	Conc. [pg/µl]	Molarity [pmol/l]	Observations
1	◀	35	125.00	5,411.3	Lower Marker
2		344	2,174.56	9,573.7	
3		368	939.91	3,869.0	
4		376	391.46	1,577.2	
5		390	2,561.75	9,955.1	
6	▶	10,380	75.00	10.9	Upper Marker

Region table for sample 2 : Old 1

From [bp]	To [bp]	Corr. Area	% of Total	Average Size [bp]	Size distribution in CV [%]	Conc. [pg/µl]	Molarity [pmol/l]	Co lor
200	1,000	2,378.8	97	391	20.9	6,639.68	26,983.9	

 © Copyright 2003 - 2018 Agilent, Inc.

Assay Class: High Sensitivity DNA Assay
Data Path: C:\...say_DEDAE00921_2022-02-25_19-55-41_hMeDIP_cerebellum_IP.xad

Created: 2/25/2022 7:55:40 PM
Modified: 2/25/2022 8:44:45 PM

Electropherogram Summary Continued ...

sample 3

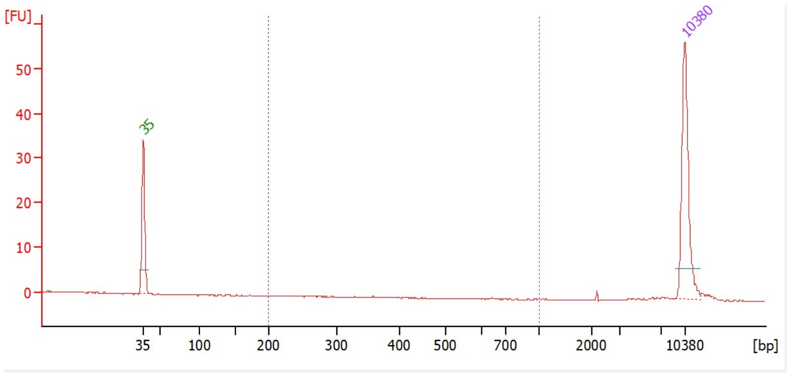

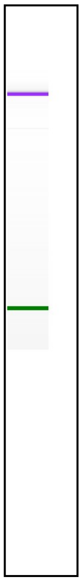

Overall Results for sample 3 : sample 3

Number of peaks found: 0
Corr. Area 1: 0.0
Noise: 0.1

Peak table for sample 3 : sample 3

Peak		Size [bp]	Conc. [pg/µl]	Molarity [pmol/l]	Observations
1	◀	35	125.00	5,411.3	Lower Marker
2	▶	10,380	75.00	10.9	Upper Marker

Region table for sample 3 : sample 3

From [bp]	To [bp]	Corr. Area	% of Total	Average Size [bp]	Size distribution in CV [%]	Conc. [pg/µl]	Molarity [pmol/l]	Co lor
200	1,000	0.0	0	0	0.0	0.00	0.0	

 © Copyright 2003 - 2018 Agilent, Inc.

Assay Class: High Sensitivity DNA Assay
Data Path: C:\...say_DEDAE00921_2022-02-25_19-55-41_hMeDIP_cerebellum_IP.xad

Created: 2/25/2022 7:55:40 PM
Modified: 2/25/2022 8:44:45 PM

Electropherogram Summary Continued ...

Young 2

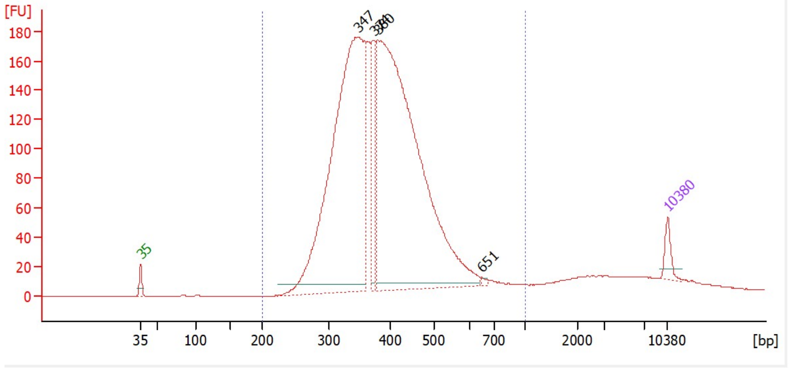

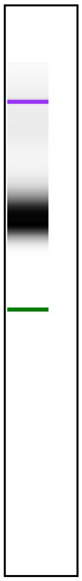

Overall Results for sample 4 : **Young 2**

Number of peaks found: 4

Corr. Area 1: 3,111.5

Noise: 0.1

Peak table for sample 4 : **Young 2**

Peak		Size [bp]	Conc. [pg/µl]	Molarity [pmol/l]	Observations
1	◀	35	125.00	5,411.3	Lower Marker
2		347	3,977.63	17,370.6	
3		374	448.56	1,817.2	
4		380	4,552.27	18,173.4	
5		651	14.86	34.6	
6	▶	10,380	75.00	10.9	Upper Marker

Region table for sample 4 : **Young 2**

From [bp]	To [bp]	Corr. Area	% of Total	Average Size [bp]	Size distribution in CV [%]	Conc. [pg/µl]	Molarity [pmol/l]	Co lor
200	1,000	3,111.5	95	395	21.7	9,539.72	38,465.1	

© Copyright 2003 - 2018 Agilent, Inc.

Assay Class: High Sensitivity DNA Assay
Data Path: C:\...say_DEDAE00921_2022-02-25_19-55-41_hMeDIP_cerebellum_IP.xad

Created: 2/25/2022 7:55:40 PM
Modified: 2/25/2022 8:44:45 PM

Electropherogram Summary Continued ...

Old 2

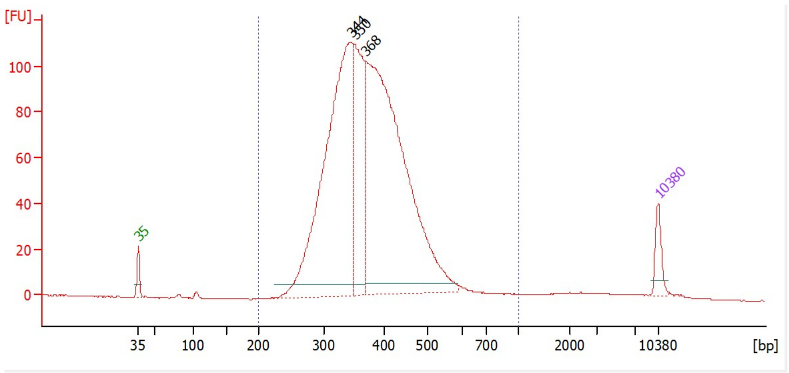

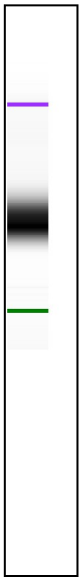

Overall Results for sample 5 : Old 2

Number of peaks found: 3

Corr. Area 1: 1,809.6

Noise: 0.2

Peak table for sample 5 : Old 2

Peak		Size [bp]	Conc. [pg/µl]	Molarity [pmol/l]	Observations
1	◀	35	125.00	5,411.3	Lower Marker
2		344	2,234.02	9,847.8	
3		350	715.92	3,095.4	
4		368	2,765.45	11,375.7	
5	▶	10,380	75.00	10.9	Upper Marker

Region table for sample 5 : Old 2

From [bp]	To [bp]	Corr. Area	% of Total	Average Size [bp]	Size distribution in CV [%]	Conc. [pg/µl]	Molarity [pmol/l]	Co lor
200	1,000	1,809.6	97	382	20.3	5,872.95	24,340.4	

 © Copyright 2003 - 2018 Agilent, Inc.

Assay Class: High Sensitivity DNA Assay
Data Path: C:\...say_DEDAE00921_2022-02-25_19-55-41_hMeDIP_cerebellum_IP.xad

Created: 2/25/2022 7:55:40 PM
Modified: 2/25/2022 8:44:45 PM

Electropherogram Summary Continued ...

sample 6

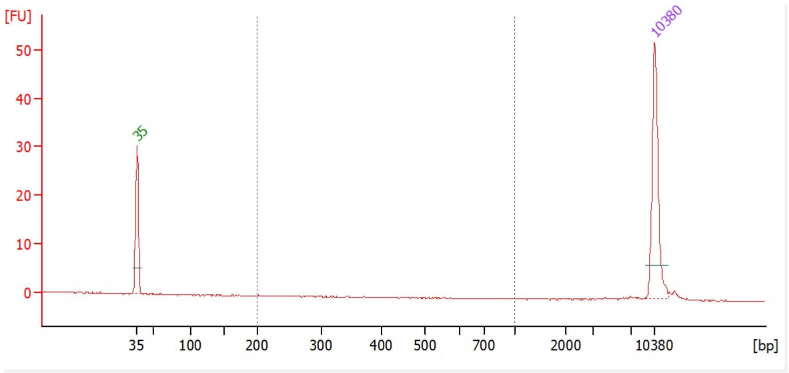

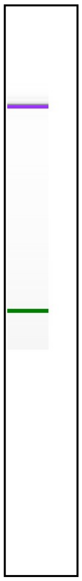

Overall Results for sample 6 : sample 6

Number of peaks found: 0

Corr. Area 1: 0.0

Noise: 0.1

Peak table for sample 6 : sample 6

Peak		Size [bp]	Conc. [pg/µl]	Molarity [pmol/l]	Observations
1	◀	35	125.00	5,411.3	Lower Marker
2	▶	10,380	75.00	10.9	Upper Marker

Region table for sample 6 : sample 6

From [bp]	To [bp]	Corr. Area	% of Total	Average Size [bp]	Size distribution in CV [%]	Conc. [pg/µl]	Molarity [pmol/l]	Color
200	1,000	0.0	0	0	0.0	0.00	0.0	

 © Copyright 2003 - 2018 Agilent, Inc.

Assay Class: High Sensitivity DNA Assay
Data Path: C:\...say_DEDAE00921_2022-02-25_19-55-41_hMeDIP_cerebellum_IP.xad

Created: 2/25/2022 7:55:40 PM
Modified: 2/25/2022 8:44:45 PM

Electropherogram Summary Continued ...

Young 3

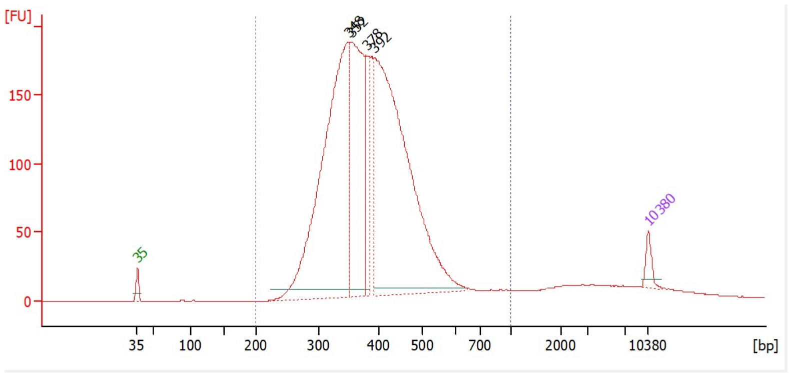

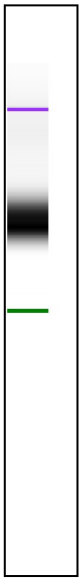

Overall Results for sample 7 : Young 3

Number of peaks found: 4
Corr. Area 1: 3,242.1
Noise: 0.1

Peak table for sample 7 : Young 3

Peak		Size [bp]	Conc. [pg/µl]	Molarity [pmol/l]	Observations
1	◀	35	125.00	5,411.3	Lower Marker
2		348	3,508.72	15,289.4	
3		352	1,662.44	7,162.0	
4		378	481.37	1,927.1	
5		392	3,867.58	14,943.8	
6	▶	10,380	75.00	10.9	Upper Marker

Region table for sample 7 : Young 3

From [bp]	To [bp]	Corr. Area	% of Total	Average Size [bp]	Size distribution in CV [%]	Conc. [pg/µl]	Molarity [pmol/l]	Co lor
200	1,000	3,242.1	95	394	21.6	10,305.69	41,658.4	

 © Copyright 2003 - 2018 Agilent, Inc.

Assay Class: High Sensitivity DNA Assay
Data Path: C:\...say_DEDAE00921_2022-02-25_19-55-41_hMeDIP_cerebellum_IP.xad

Created: 2/25/2022 7:55:40 PM
Modified: 2/25/2022 8:44:45 PM

Electropherogram Summary Continued ...

Old 3

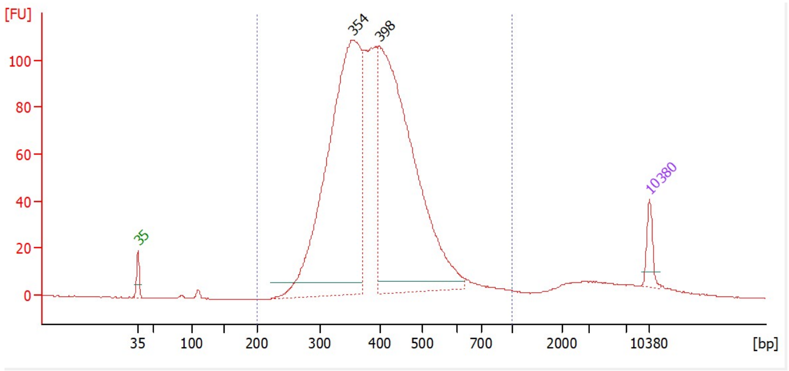

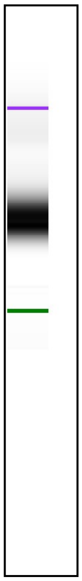

Overall Results for sample 8 : **Old 3**

Number of peaks found:	2	Corr. Area 1:	1,928.2
Noise:	0.1		

Peak table for sample 8 : **Old 3**

Peak		Size [bp]	Conc. [pg/µl]	Molarity [pmol/l]	Observations
1	◀	35	125.00	5,411.3	Lower Marker
2		354	2,813.31	12,039.0	
3		398	2,610.17	9,925.9	
4	▶	10,380	75.00	10.9	Upper Marker

Region table for sample 8 : **Old 3**

From [bp]	To [bp]	Corr. Area	% of Total	Average Size [bp]	Size distribution in CV [%]	Conc. [pg/µl]	Molarity [pmol/l]	Co lor
200	1,000	1,928.2	95	403	21.7	6,693.80	26,455.5	■

 © Copyright 2003 - 2018 Agilent, Inc.

Assay Class: High Sensitivity DNA Assay
Data Path: C:\...say_DEDAE00921_2022-02-25_19-55-41_hMeDIP_cerebellum_IP.xad

Created: 2/25/2022 7:55:40 PM
Modified: 2/25/2022 8:44:45 PM

Electropherogram Summary Continued ...

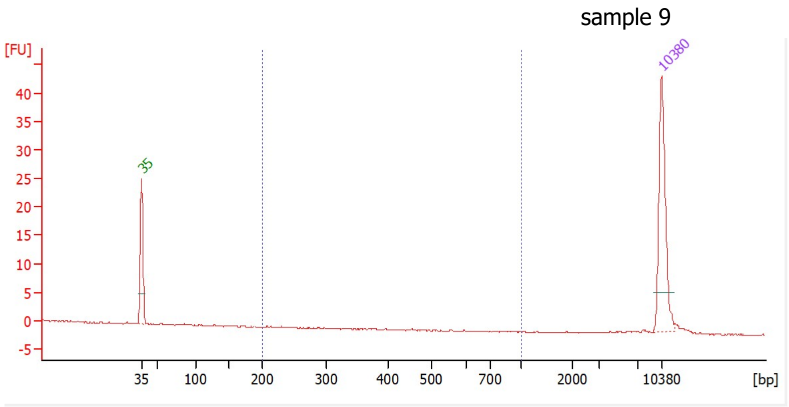

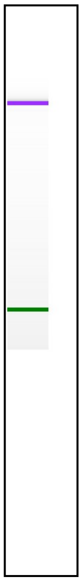

Overall Results for sample 9 : sample 9

Number of peaks found:	0	Corr. Area 1:	0.0
Noise:	0.1		

Peak table for sample 9 : sample 9

Peak		Size [bp]	Conc. [pg/µl]	Molarity [pmol/l]	Observations
1	◀	35	125.00	5,411.3	Lower Marker
2	▶	10,380	75.00	10.9	Upper Marker

Region table for sample 9 : sample 9

From [bp]	To [bp]	Corr. Area	% of Total	Average Size [bp]	Size distribution in CV [%]	Conc. [pg/µl]	Molarity [pmol/l]	Co lor
200	1,000	0.0	0	0	0.0	0.00	0.0	

© Copyright 2003 - 2018 Agilent, Inc.

Assay Class: High Sensitivity DNA Assay
Data Path: C:\...say_DEDAE00921_2022-02-25_19-55-41_hMeDIP_cerebellum_IP.xad

Created: 2/25/2022 7:55:40 PM
Modified: 2/25/2022 8:44:45 PM

Electropherogram Summary Continued ...

Young 4

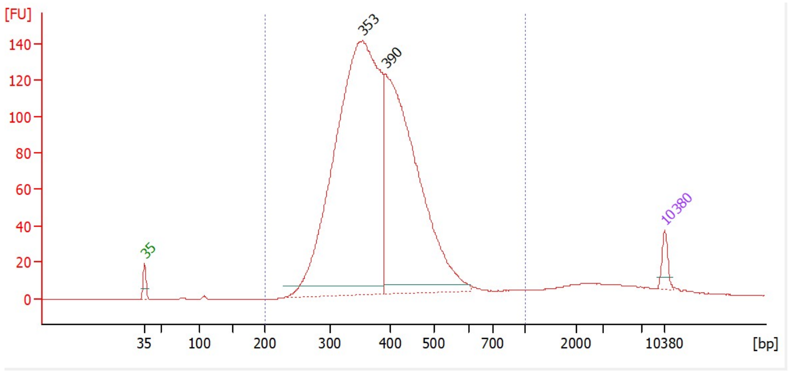

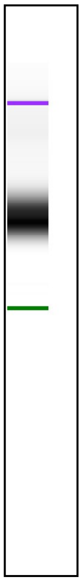

Overall Results for sample 10 : Young 4

Number of peaks found: 2

Corr. Area 1: 2,262.9

Noise: 0.1

Peak table for sample 10 : Young 4

Peak		Size [bp]	Conc. [pg/µl]	Molarity [pmol/l]	Observations
1	◀	35	125.00	5,411.3	Lower Marker
2		353	5,816.39	24,932.4	
3		390	3,431.69	13,318.0	
4	▶	10,380	75.00	10.9	Upper Marker

Region table for sample 10 : Young 4

From [bp]	To [bp]	Corr. Area	% of Total	Average Size [bp]	Size distribution in CV [%]	Conc. [pg/µl]	Molarity [pmol/l]	Co lor
200	1,000	2,262.9	96	387	20.7	9,551.81	39,121.4	

© Copyright 2003 - 2018 Agilent, Inc.

Assay Class: High Sensitivity DNA Assay
Data Path: C:\...say_DEDAE00921_2022-02-25_19-55-41_hMeDIP_cerebellum_IP.xad

Created: 2/25/2022 7:55:40 PM
Modified: 2/25/2022 8:44:45 PM

Electropherogram Summary Continued ...

Old 4

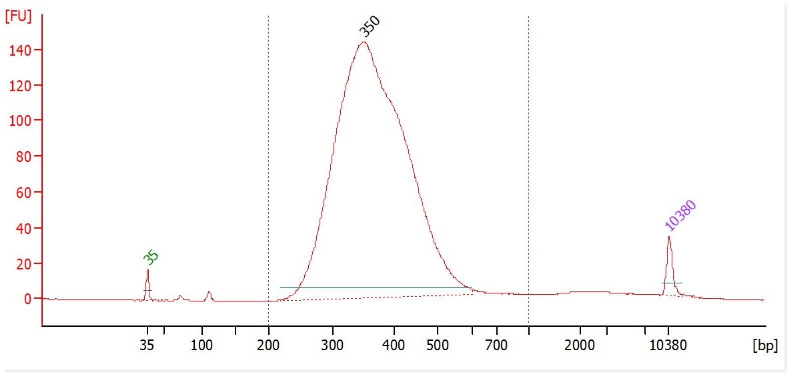

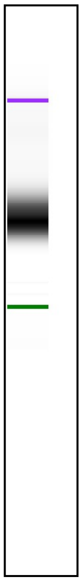

Overall Results for sample 11 : Old 4

Number of peaks found: 1   Corr. Area 1: 2,260.7

Noise: 0.1

Peak table for sample 11 : Old 4

Peak		Size [bp]	Conc. [pg/µl]	Molarity [pmol/l]	Observations
1	◄	35	125.00	5,411.3	Lower Marker
2		350	8,831.81	38,287.0	
3	►	10,380	75.00	10.9	Upper Marker

Region table for sample 11 : Old 4

From [bp]	To [bp]	Corr. Area	% of Total	Average Size [bp]	Size distribution in CV [%]	Conc. [pg/µl]	Molarity [pmol/l]	Co lor
200	1,000	2,260.7	97	377	21.1	8,917.54	37,503.1	■

 © Copyright 2003 - 2018 Agilent, Inc.

Assay Class: High Sensitivity DNA Assay
Data Path: C:\...say_DEDAE00921_2022-02-25_19-55-41_hMeDIP_cerebellum_IP.xad
Created: 2/25/2022 7:55:40 PM
Modified: 2/25/2022 8:44:45 PM

Gel Image

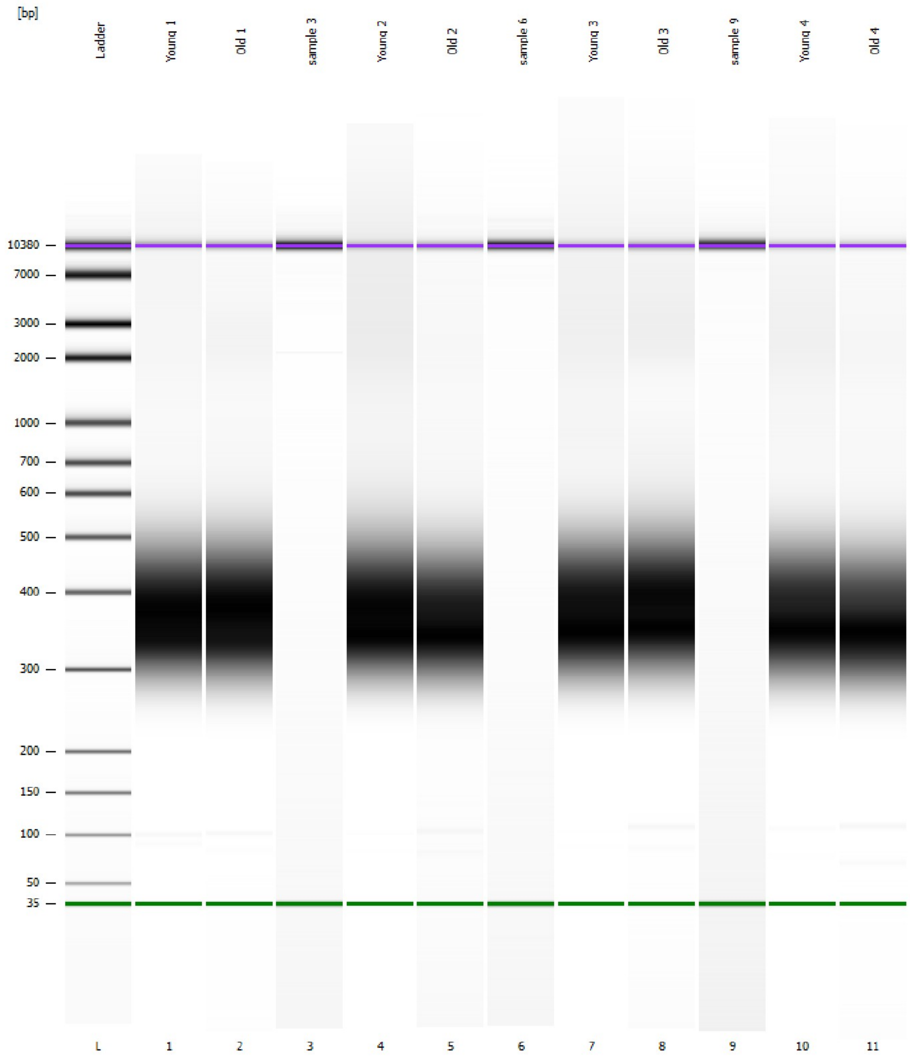

© Copyright 2003 - 2018 Agilent, Inc.
